# B7-H3 promotes skin photodamage through ITGA2-NRF2-TFAM-mediated mitochondrial oxidative stress

**DOI:** 10.1038/s42003-026-10387-6

**Published:** 2026-05-28

**Authors:** Yuanyuan Jia, Qiange Zhang, Fangying Su, Yuan Zhu, Siji Chen, Jingyi Yang, Qiuyu Mao, Yiwen Zhang, Yuting Yang, Sisi Ding, Cuiping Liu, Xueguang Zhang, Dan Luo, Wei Min, Lei Cao

**Affiliations:** 1 Jiangsu Institute of Clinical Immunology, The First Affiliated Hospital of Soochow University, Suzhou, Jiangsu Province China; 2 Department of Dermatology, JiangSu Province Hospital, The First Affiliated Hospital With Nanjing Medical University, Nanjing, Jiangsu Province China; 3 Jiangsu Key Laboratory of Clinical Immunology, Soochow University, Suzhou, Jiangsu Province China; 4 Jiangsu Key Laboratory of Gastrointestinal Tumor Immunology, The First Affiliated Hospital of Soochow University, Suzhou, Jiangsu Province China; 5 Department of Dermatology, People’s Hospital of Guangxi Zhuang Autonomous Region, Nanning, Guangxi Zhuang Autonomous Region China; 6 Department of Dermatology, First People’s Hospital of Changshu City, Changshu Hospital Affiliated of Soochow University, Changshu, Jiangsu Province China; 7 NanTong Tong Zhou Hospital of Chinese Traditional Medicine, Nantong, Jiangsu China; 8 Department of Dermatology, Kunshan First People’s Hospital, Suzhou, Jiangsu Province China; 9 Department of Dermatology, Minhang Hospital, Fudan University, Shanghai, China

**Keywords:** Skin diseases, Cell biology

## Abstract

Ultraviolet radiation (UVR) exposure drives skin photodamage, which constitutes the fundamental basis of both photoaging and photocarcinogenesis, by disrupting mitochondrial homeostasis and inducing reactive oxygen species (ROS) accumulation. Although B7-H3 (CD276) is recognized as an immune-checkpoint molecule involved in tumor metabolism, its role in UVR-induced cutaneous photodamage and the associated redox imbalance remains undefined. Here we show that B7-H3 is elevated in acute and chronic photodamaged human skin as well as in UVR-induced mouse (female) and cell models. Genetic deletion or antibody blockade of B7-H3 attenuates ROS accumulation, preserves mitochondrial function, and reduces photodamage. Mechanistically, B7-H3 interacts with integrin α2 (ITGA2) to impair antioxidant signaling by inhibiting the nuclear translocation of nuclear factor erythroid 2-related factor 2 (NRF2) and disrupting the nuclear respiratory factor 1 (NRF1)-transcription factor A, mitochondrial (TFAM) pathway, ultimately disrupting mitochondrial biogenesis and redox balance. Furthermore, treatment with an anti-B7-H3 blocking monoclonal antibody alleviates photodamage and preserves mitochondrial function. Collectively, these findings indicate that the skin-resident B7-H3/ITGA2 interaction contributes to photodamage via disrupting TFAM expression, suggesting that targeting B7-H3 may represent a promising therapeutic strategy for skin photodamage and related disorders.

**Figure:**
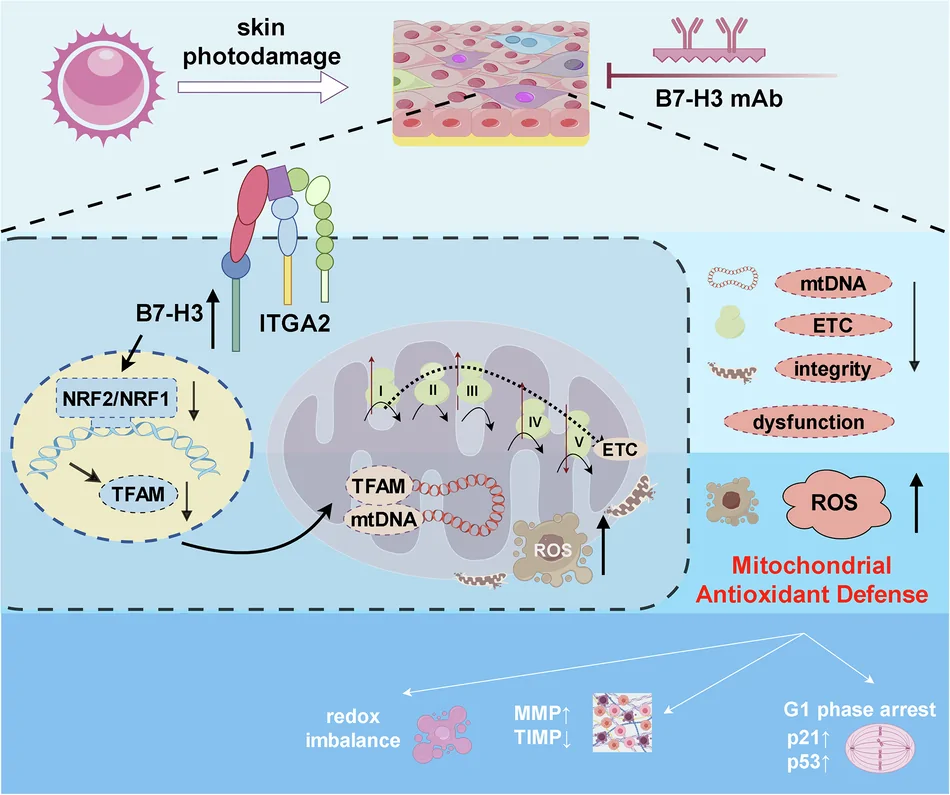

## Abstract (web summary)

B7-H3 promotes skin photodamage by disrupting mitochondrial oxidative stress via the ITGA2- NRF2-TFAM axis, and its blockade attenuates photodamage, suggesting a potential therapeutic strategy.

## Introduction

Skin is the largest organ of the human body and the first line of defense against external environmental damage. Indeed, skin maintains tissue homeostasis against ultraviolet radiation (UVR), which can trigger inflammation, oxidative stress, and senescence. UVR can be classified into three categories according to the wavelength: UVA (320–400 nm), UVB (280–320 nm), and UVC (100–280 nm). UVA can penetrate deeply into the skin and exert a direct effect on the dermal fibroblasts that produce collagen and elastin. UVB impacts epidermal keratinocytes, a major contributor to photodamage, including photoaging and malignancies such as cutaneous squamous cell carcinoma (CSCC) and basal cell carcinoma (BCC) of the skin^1,2^. Skin cancers arising from chronic photodamage impose a mounting global health burden^3^. Beyond oncogenesis, photodamage has systemic implications. Emerging evidence also suggests photodamage accelerates systemic aging beyond oncogenesis^4^. Therefore, elucidating the molecular mechanisms governing UVR-induced skin damage holds medical, social, and economic significance.

Our previous studies have shown that UVR can induce photodamage and photoaging in epidermis and dermis through multiple pathways, including the production of reactive oxygen species (ROS), activation of the p53/p21 axis, DNA damage, and upregulation of matrix metalloproteinases (MMPs)^5,6^. Moreover, mitochondrial dysfunction and redox imbalance establish a feed-forward loop that amplifies oxidative injury to cellular proteins, lipids, and mitochondrial DNA (mtDNA), culminating in tissue degeneration and disease^7–10^.

Mitochondria sustain cellular energy production via oxidative phosphorylation (OXPHOS), a paradoxical process that generates ATP while inevitably producing ROS as electron transport chain (ETC) by-products. To counteract this inherent redox burden, mitochondria employ integrated antioxidant systems and stress-response pathways, with transcription factor A, mitochondrial (TFAM) serving as a linchpin: it compacts mtDNA into nucleoid-like structures to shield against oxidative lesions while facilitating respiratory complex assembly^11^. Concurrently, nuclear factor erythroid 2-related factor 2 (NRF2)**-**nuclear respiratory factor 1 (NRF1) pathway orchestrates TFAM-dependent mitochondrial biogenesis while amplifying antioxidant defenses—a dual mechanism critical for balancing energy demands with ROS neutralization^12^. Mounting evidence implicates ROS and mitochondrial functional dynamics as central drivers of UVR-induced skin pathologies, including keratinocyte apoptosis, photoaging, and photocarcinogenesis.

B7-H3 (also known as CD276), a co-stimulatory immune molecule, has been implicated in diverse biological processes, including immune modulation, oncogenesis, metabolism, and oxidative stress regulation^13–16^. While B7-H3 is well characterized in tumor immunity and autoimmune pathologies, its role in skin regulation and mitochondrial redox balance under phototoxic stress remains unexplored. Our previous work revealed elevated B7-H3 expression in malignant skin lesions—severe manifestations of chronic photodamage, and demonstrated that B7-H3 downregulation suppresses the proliferation and invasiveness of skin tumor cells, underscoring B7-H3 as a key driver of UVR-promoted skin carcinogenesis. However, the mechanistic contribution of B7-H3 to UVR-induced photodamage and its regulatory pathways remain poorly understood.

In this report, we proposed a hypothesis that B7-H3 contributes to photodamage by disrupting mitochondrial redox homeostasis. To comprehensively examine it, we employed B7-H3 knockout (KO) mice, along with human skin fibroblasts (HSF) and immortalized keratinocytes (HaCaT), to explore the role and mechanisms of B7-H3 in mediating UVR-induced mitochondrial damage. Our findings reveal a role not previously described for B7-H3 in controlling mitochondrial oxidative stress, providing mechanistic insight into the regulation of cutaneous photodamage and identifying a potential therapeutic target for preventing UVR-induced skin injury.

## Results

### B7-H3 expression is upregulated in acute and chronic photodamaged skin

**Fig. 1:**
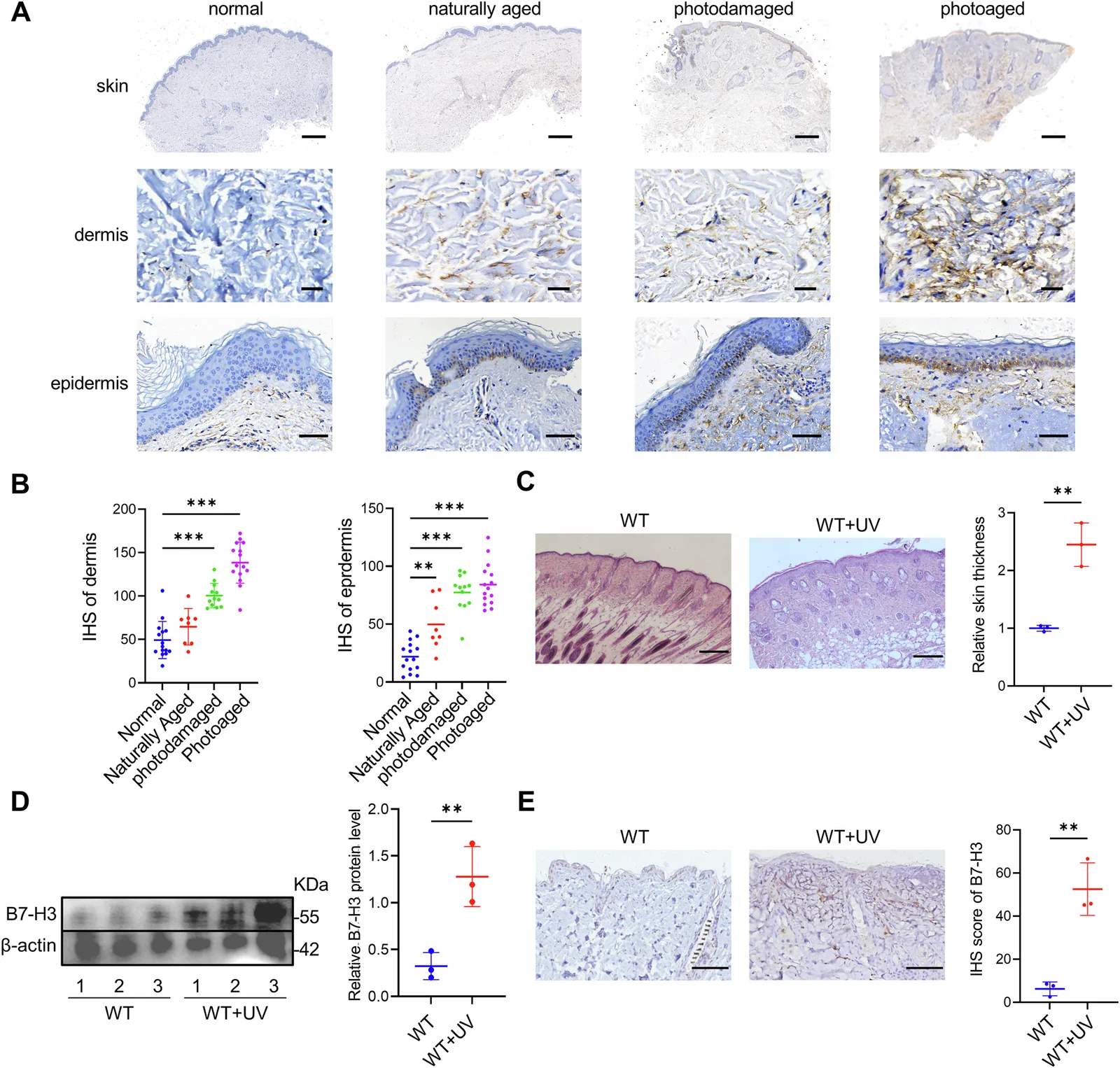
B7-H3 is upregulated in photodamaged skin. **A**, **B** Expression of B7-H3 in normal (*n* = 15), naturally aged (*n* = 8), photodamaged (*n* = 12), and photoaged (*n* = 15) skin tissues, including dermis and epidermis. **C**–**E** Mice were exposed to UVB irradiation (300 mJ/cm^2^) daily for three consecutive days and skin samples were collected 24 h after the final exposure. Scale bars: 200 μm (skin), 20 μm (dermis), and 100 μm (epidermis) as indicated in the figure. **C** H&E staining images and relative skin thickness of UVB-irradiated mice. Scale bars: 200 μm as indicated in the figure. Western blot (**D**) and IHC (**E**) analysis of B7-H3 in photodamaged skin of mice. Scale bars: 100 μm as indicated in the figure. *n* = 3 female mice or *n* = 3 independent experiments. * represents *P* < 0.05; ** represents *P* < 0.01; and *** represents *P* < 0.001. Error bars represent mean ± SD.

Our immunohistochemistry (IHC) results of tissue microarrays (63 cases of CSCC, 28 cases of BCC, 7 cases of melanoma, and 14 normal tissues) indicated that B7-H3 was highly expressed in CSCC and BCC tissues—UV-associated non-melanoma skin cancers commonly linked to chronic sun exposure (Supplementary Fig. 1A–C). Building on these findings, we assessed B7-H3 expression in naturally aged, sun-exposed (photodamaged and photoaged), and normal human skin tissues. Our results showed that both acute and chronic UVR exposures triggered marked B7-H3 upregulation, with elevated expression observed throughout the epidermal and dermal compartments (Fig. 1A, B).

To validate these findings in vivo, we established a photodamage mouse model using C57BL/6 J mice subjected to 300 mJ/cm^2^ UVB irradiation for 3 consecutive days. This regimen induced clear histological features of photodamage, including skin erythema, edema, scaling, and structural disorganization (Fig. 1C). Western blot and IHC confirmed upregulation of B7-H3 protein in UVB-exposed skin compared to non-irradiated controls (Fig. 1D, E).

Given the enhanced B7-H3 expression in keratinocytes and fibroblasts within acute and chronic photodamaged tissues, we next evaluated B7-H3 induction in vitro using HSFs and HaCaT cells exposed to graded UVA or UVB doses (Supplementary Fig. 1D, E). In both cell types, B7-H3 expression increased in a dose-dependent manner, peaking at 10 J/cm^2^ UVA (HSF) and 9 mJ/cm^2^ UVB (HaCaT) (Supplementary Fig. 1F, G). At higher UVR doses, B7-H3 levels declined, likely due to severe cellular damage surpassing reparative capacity.

### Reduction of B7-H3 mitigates UVR-induced ROS accumulation

**Fig. 2:**
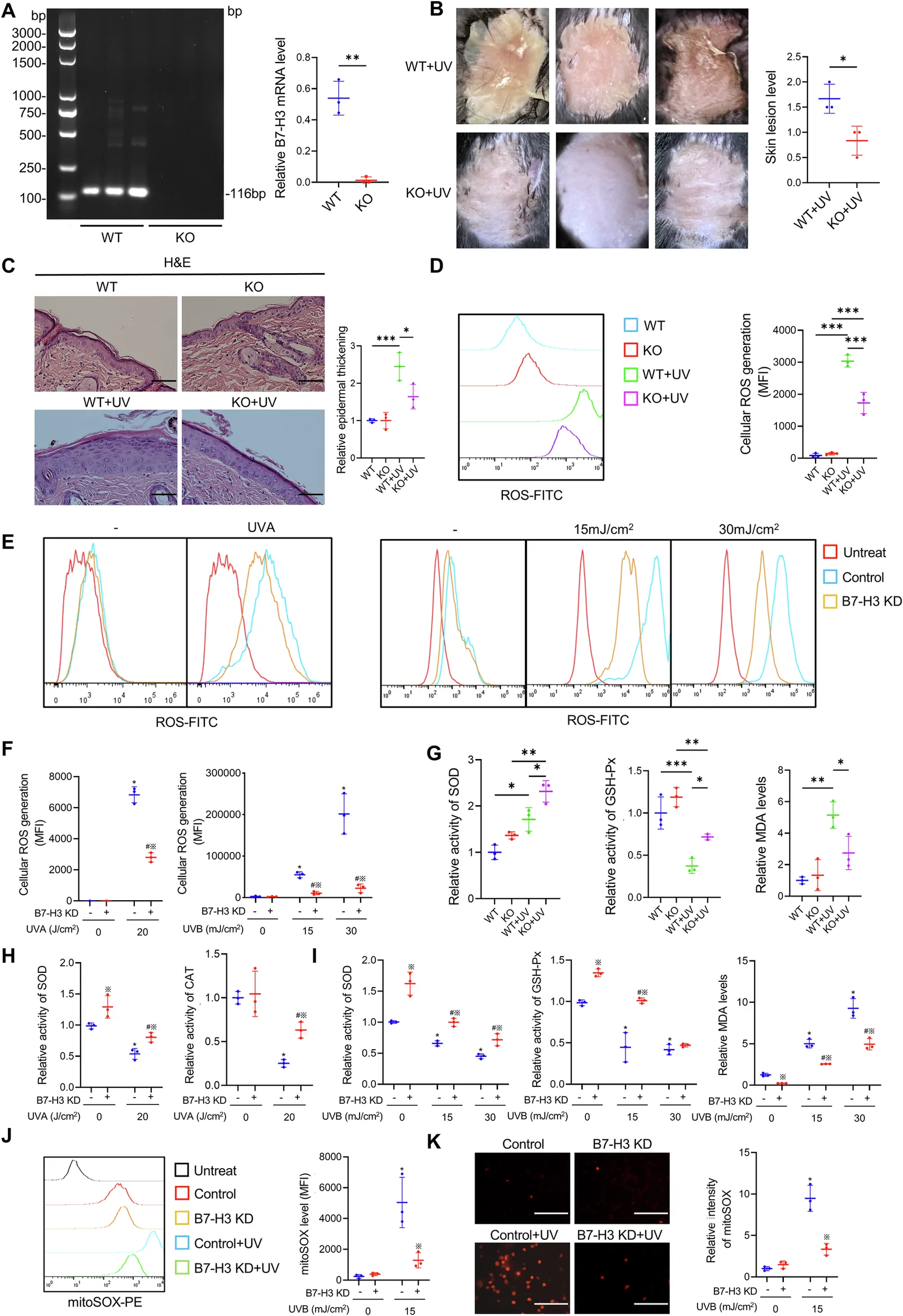
B7-H3 induces skin acute photodamage. Irradiation conditions: For in vivo experiments, mice were exposed to UVB irradiation (300 mJ/cm^2^) daily for three consecutive days and skin samples were collected 24 h after the final exposure. For in vitro experiments, HSF and HaCaT cells were irradiated once with indicated doses and harvested 24 h post-irradiation. UVB doses (15 and 30 mJ/cm^2^ for HaCaT) and UVA dose (20 J/cm^2^ for HSF) were selected to ensure robust and reproducible oxidative stress responses. **A**RT-PCR and qRT-PCR analysis of B7-H3 in B7-H3 KO mice. **B** The optical images and photodamage score of skin with UVB. **C** H&E staining images and relative epidermal thickening score of mouse skin. Scale bars: 50 μm as indicated in the figure. **D** ROS levels in mouse skin tissues were detected by FACS analysis. **E**, **F** FACS analysis of ROS in photodamaged HSF (UVA 20 J/cm^2^) and HaCaT (UVB 15 or 30 mJ/cm^2^) cells. **G** SOD, GSH-Px, and lipid peroxidation as MDA levels of mouse skin tissues. SOD, CAT, GSH-Px activity, and MDA levels of HSF (**H**, UVA 20 J/cm^2^) and HaCaT (**I**, UVB 15 or 30 mJ/cm^2^) cells. FACS analysis (**J**) and the fluorescent pictures (**K**) representing the fluorescence of mitoSOX in HaCaT cells irradiated once with UVB (15 mJ/cm^2^). Scale bars: 200 μm as indicated in the figure. *n* = 3 female mice or independent experiments. **A**–**D**, **G**: * represents *P* < 0.05; ** represents *P* < 0.01; *** represents *P* < 0.001. **F**, **H**–**K** * *P* < 0.05 (Control + UVR vs Control); # *P* < 0.05 (B7-H3 KD + UVR vs B7-H3 KD); ※ *P* < 0.05 (B7-H3 KD + UVR vs Control + UVR, at the same dose). Error bars represent mean ± SD.

To further explore whether B7-H3 inhibition can protect skin from photodamage, we established stable B7-H3 KO mice and B7-H3 knockdown (KD) cell models (Supplementary Fig. 2A–D). The effective deletion of B7-H3 expression was confirmed (Fig. 2A, Supplementary Fig. 2B–D). Following UVR exposure, the severity of acute photodamage, including erythema, scaling, dryness, and epidermal thickening, was markedly reduced in KO mice compared to wild-type (WT) controls, as assessed by both macroscopic scoring and histological analysis (Fig. 2B, C). ROS cause oxidative damage to many biological molecules including DNA, promote p21 generation and cell cycle arrest, induce the expression of MMPs, accelerate the rate of telomere shortening, and contribute to the initiation and progression of photodamage^17,18^. Consistent with this, UVR significantly increased ROS levels in both in vivo and in vitro photodamage models compared to non-irradiated controls. Importantly, this UVR-induced ROS accumulation was markedly attenuated in B7-H3 KO mice, as well as in B7-H3 KD HaCaT and HSF cells, when compared to their respective WT or control groups (Fig. 2D–F). Consistent with the elevated ROS levels, UVR induced significant alterations in antioxidant enzyme activities and oxidative damage markers. In vivo, UVB-exposed WT mouse skin exhibited a compensatory increase in superoxide dismutase (SOD) activity, alongside a significant reduction in glutathione peroxidase (GSH-Px) activity and elevated malondialdehyde (MDA) levels, indicating an imbalance in antioxidant defense and subsequent lipid peroxidation. Compared to UVB-exposed WT mice, B7-H3 KO mice displayed further enhanced SOD activity, restored GSH-Px activity, and markedly reduced MDA accumulation (Fig. 2G). In UVB-irradiated HaCaT and UVA-irradiated HSF cells, knockdown of B7-H3 significantly attenuated the suppression of SOD, catalase (CAT), and GSH-Px activities and reduced MDA accumulation compared to control cells (Fig. 2H, I). Furthermore, B7-H3 knockdown also diminished mitochondrial-specific ROS production, as evidenced by reduced mitoSOX staining, further supporting a role for B7-H3 as a regulator of oxidative stress and mitochondrial redox imbalance (Fig. 2J, K).

### Decline of B7-H3 attenuates UVR-induced early stress responses associated with photoaging

**Fig. 3:**
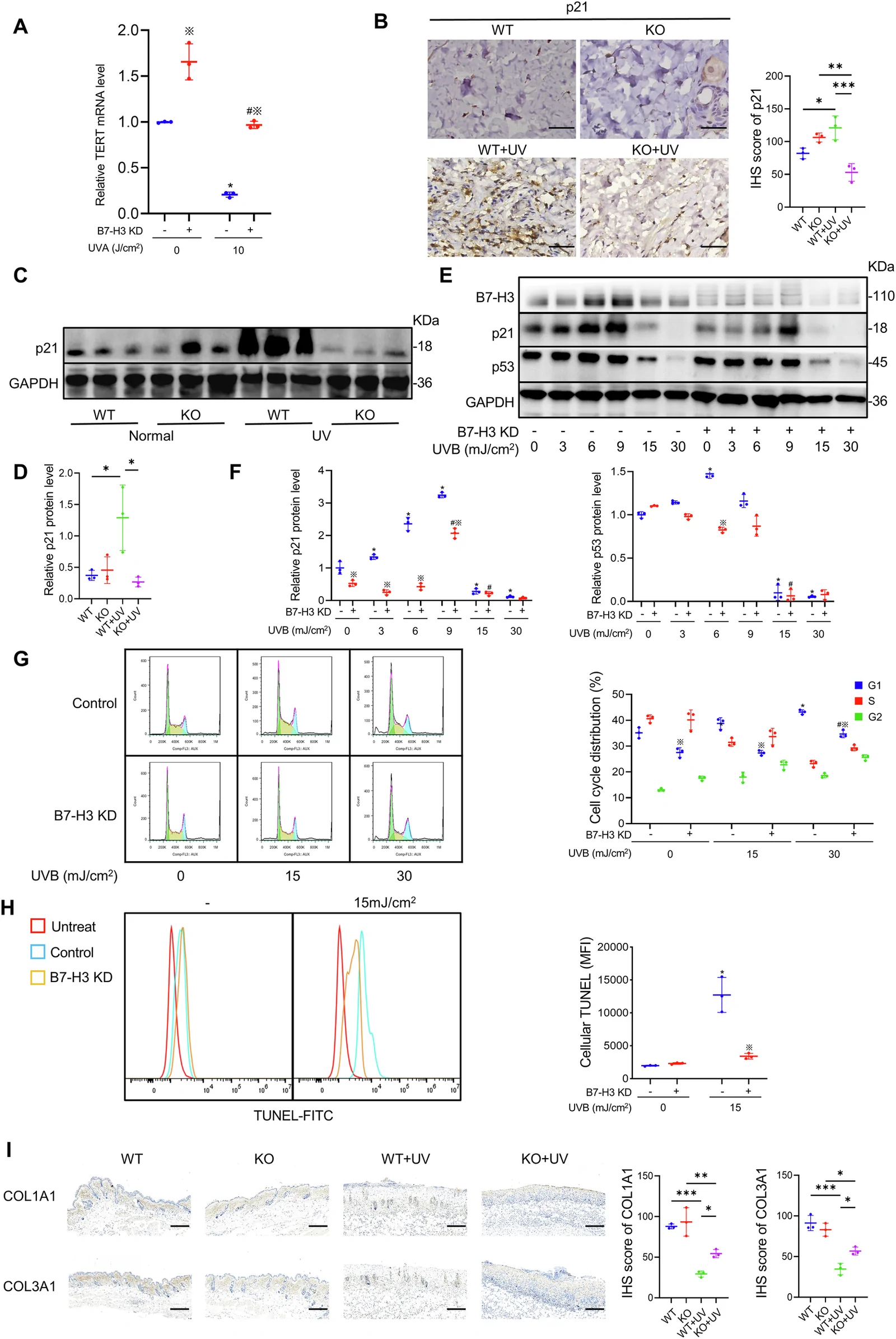
B7-H3 promotes photoaging-associated molecular changes in acute UVR-exposed skin. Irradiation conditions: For in vivo experiments, mice were exposed to UVB irradiation (300 mJ/cm^2^) daily for three consecutive days and skin samples were collected 24 h after the final exposure (**B**–**D**, **I**, **J**). For in vitro experiments, HSF cells were irradiated once with UVA (10 J/cm^2^) and harvested 24 h post-irradiation (**A**). HaCaT cells were irradiated once with UVB at the indicated doses (0, 3, 6, 9, 15, 30 mJ/cm^2^ in E, F; 15 and 30 mJ/cm^2^ in G; 15 mJ/cm^2^ in H) and harvested 24 h post-irradiation. **A** mRNA level of *TERT* in HSF cells. Protein levels of p21 in mouse skin detected by IHC (**B**) and western blot (**C**), with corresponding grayscale analysis (**D**). Scale bars: 50 μm as indicated in the figure. **E**, **F** Western blot analysis of p21 and p53 with corresponding grayscale analysis in HaCaT cells. **G** FACS analysis of the cell cycle distribution in HaCaT cells treated with UVB. **H** FACS analysis of TUNEL fluorescence intensity in HaCaT cells. **I**, **J** IHC of COL1A1 and COL3A1 in mouse skin sections. Scale bars: 100 μm as indicated in the figure. *n* = 3 female mice or *n* = 3 independent experiments. **B**, **D**, **I**, **J**: * represents *P* < 0.05; ** represents *P* < 0.01; *** represents *P* < 0.001. **A**, **F**, **G**: * *P* < 0.05 (Control + UVR vs Control); # *P* < 0.05 (B7-H3 KD + UVR vs B7-H3 KD); ※ *P* < 0.05 (B7-H3 KD + UVR vs Control + UVR, at the same dose). Error bars represent mean ± SD.

In addition to amplifying UVR-induced ROS generation—a hallmark of acute photodamage, we further identified that B7-H3 critically drove early stress responses that are associated with the development of photoaging. B7-H3 inhibition protected against UVA-induced reduction in telomerase reverse transcriptase (*TERT*) mRNA levels in fibroblasts (Fig. 3A). B7-H3 deficiency also reversed the UVR-induced upregulation of p21 at both mRNA and protein levels in mouse skin tissues and HSFs (Fig. 3B–D, Supplementary Fig. 3A, B). In HaCaT cells, p21 and p53 were increased at moderate UVB doses (9 mJ/cm^2^) and decreased at higher doses—patterns that were significantly blunted in B7-H3 KD cells (Fig. 3E, F, Supplementary Fig. 3C). Given the central role of the p53/p21 pathway in cell cycle arrest and senescence through G1 phase blockade, we next examined UVR-induced cell cycle alterations. Flow cytometry revealed that UVR promoted G1 arrest in photodamaged cells, which was alleviated upon B7-H3 knockdown (Fig. 3G, Supplementary Fig. 3D). We further investigated the ultimate fate of these damaged cells by assessing apoptosis. TUNEL assay demonstrated that UVR exposure significantly increased the rate of apoptotic cell death, an effect that was markedly suppressed by B7-H3 knockdown (Fig. 3H). Additionally, B7-H3 regulated extracellular matrix (ECM) homeostasis by upregulating *MMPs* and downregulating tissue inhibitors of metalloproteinases (*TIMPs*) and transforming growth factor-β (*TGF-β*) at the mRNA level, thereby promoting ECM degradation and suppressing collagen synthesis (Supplementary Fig. 3E–G). Consistently, IHC analysis revealed that UVB-induced downregulation of collagen I (COL1A1) and collagen III (COL3A1) in mouse skin was significantly rescued upon B7-H3 knockout (Fig. 3I, J), further supporting a role for B7-H3 in promoting ECM degradation and suppressing collagen synthesis at the protein level. Together, these findings identify B7-H3 as a key regulator of multiple cellular changes induced by acute UVR. These changes are associated with the development of photoaging, suggesting that targeting B7-H3 may mitigate these early stress responses.

### B7-H3 negatively regulates TFAM and leads to mitochondrial dysfunction

**Fig. 4:**
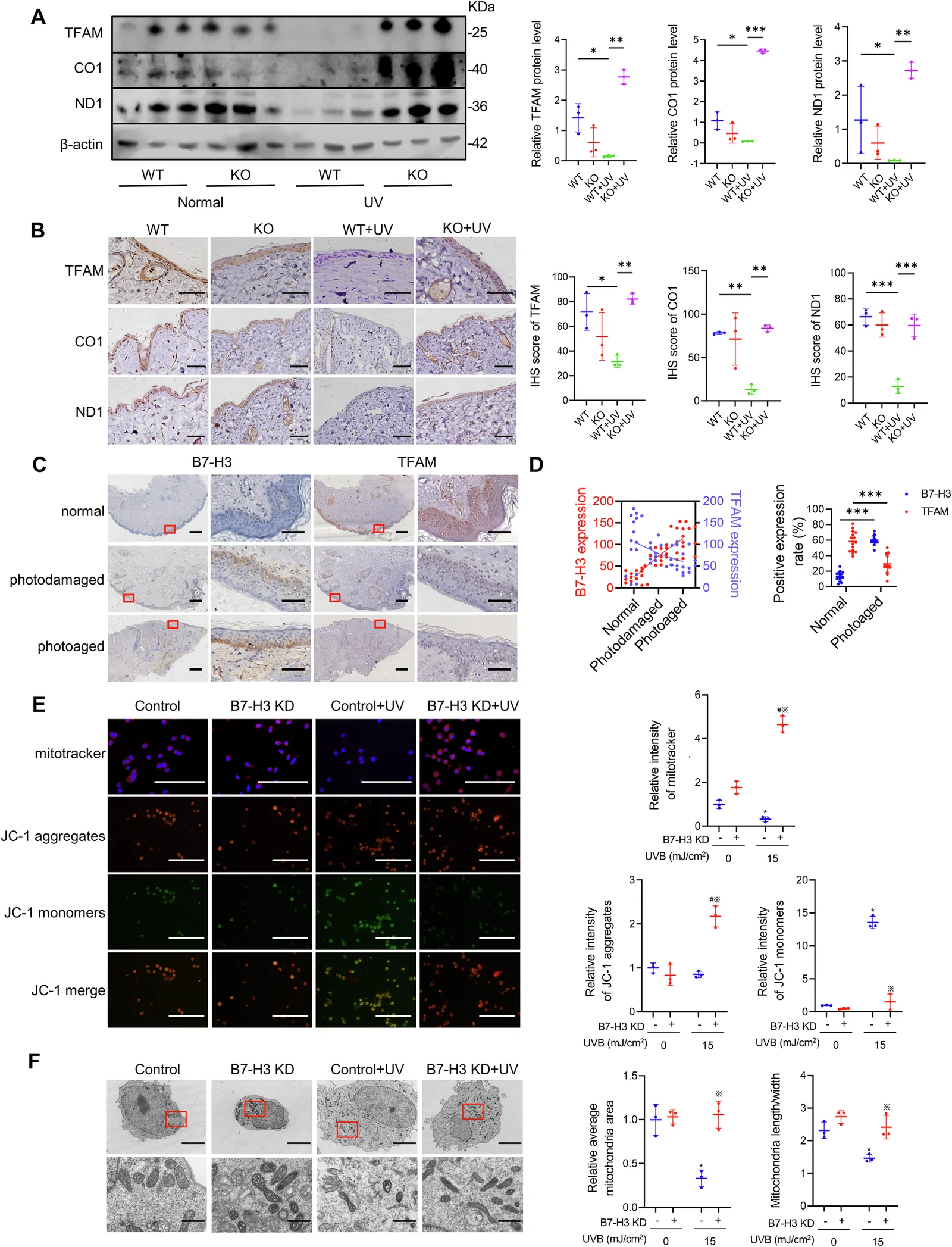
B7-H3 negatively regulates TFAM and mitochondrial dysfunction. Irradiation conditions: For in vivo experiments, mice were exposed to UVB irradiation (300 mJ/cm^2^) daily for three consecutive days and skin samples were collected 24 h after the final exposure (**A**, **B**). For in vitro experiments, HaCaT cells were irradiated once with UVB (15 mJ/cm^2^) and harvested 24 h post-irradiation (**E**, **F**). Western blot (**A**) and IHC staining (**B**) of TFAM and mtDNA-related genes in mouse skin tissues. Scale bars: 50 μm as indicated in the figure. **C**, **D** IHC staining of TFAM and B7-H3 in normal (*n* = 15), photodamaged (*n* = 12), and photoaged (*n* = 15) human skin tissues. Scale bars: 200 μm (main images) and 50 μm (enlarged views) as indicated in the figure. **E** Mitotracker, JC-1 aggregates, and JC-1 monomers in photodamaged HaCaT cells. Scale bars: 200 μm as indicated in the figure. **F** TEM of mitochondrial ultrastructure in HaCaT cells, and quantification of mitochondrial area and aspect ratio. Scale bars: 5 μm (main images) and 1 μm (enlarged views) as indicated in the figure. *n* = 3 female mice or *n* = 3 independent experiments. **A**–**C**: * represents *P* < 0.05; ** represents *P* < 0.01; *** represents *P* < 0.001. **E**, **F**: * *P* < 0.05 (Control + UVR vs Control); # *P* < 0.05 (B7-H3 KD + UVR vs B7-H3 KD); ※ *P* < 0.05 (B7-H3 KD + UVR vs Control + UVR, at the same dose). Error bars represent mean ± SD.

TFAM is a master regulator of mtDNA transcription and stability, essential for the expression of mitochondrial genes encoding components of the ETC. Notably, TFAM also limits intracellular ROS generation^19^. To elucidate the molecular mechanisms by which B7-H3 deletion attenuates ROS accumulation, we analyzed both mRNA and protein expression profiles of TFAM. Ablation of B7-H3 induced higher TFAM levels, compared to the control groups in vivo (Fig. 4A, B, Supplementary Fig. 4A). In line with the alterations in TFAM expression, UVB irradiation impaired mitochondrial respiratory function, as evidenced by reduced mtDNA content and decreased mRNA expression of mtDNA-encoded genes, cytochrome c oxidase subunit 1 (*CO1*) and NADH dehydrogenase subunit 1 (*ND1*), which were restored by B7-H3 deletion (Fig. 4A, B, Supplementary Fig. 4B). In vitro, low-dose UVB (3 and 6 mJ/cm^2^) did not significantly decrease TFAM levels in HaCaT cells. However, UVB irradiation at 9 mJ/cm^2^ markedly suppressed both TFAM mRNA and protein expression (Supplementary Fig. 4C-F). IHC further confirmed that B7-H3 expression was elevated and TFAM was reduced in human photodamaged skin (Fig. 4C, D).

Functionally, knockdown of B7-H3 preserved mitochondrial integrity, as indicated by sustained mitotracker fluorescence and decreased JC-1 monomers, reflecting intact mitochondrial membrane potential (Fig. 4E). These findings suggest that B7-H3 impairs ETC function and redox balance by downregulating TFAM and compromising mitochondrial health.

Morphologically, transmission electron microscopy (TEM) revealed that UVB irradiation caused reduced area, and disrupted aspect ratio (length/width) of mitochondria (Fig. 4F). In contrast, B7-H3 deficiency preserved mitochondrial morphology. Given that mitochondrial elongation and cristae structure are vital for compartmentalized OXPHOS, these ultrastructural findings support the biochemical data showing restored mtDNA content and respiratory gene expression in B7-H3-deficient models.

### TFAM is essential for B7-H3-mediated mitochondrial dysfunction, ROS accumulation and photodamage

**Fig. 5:**
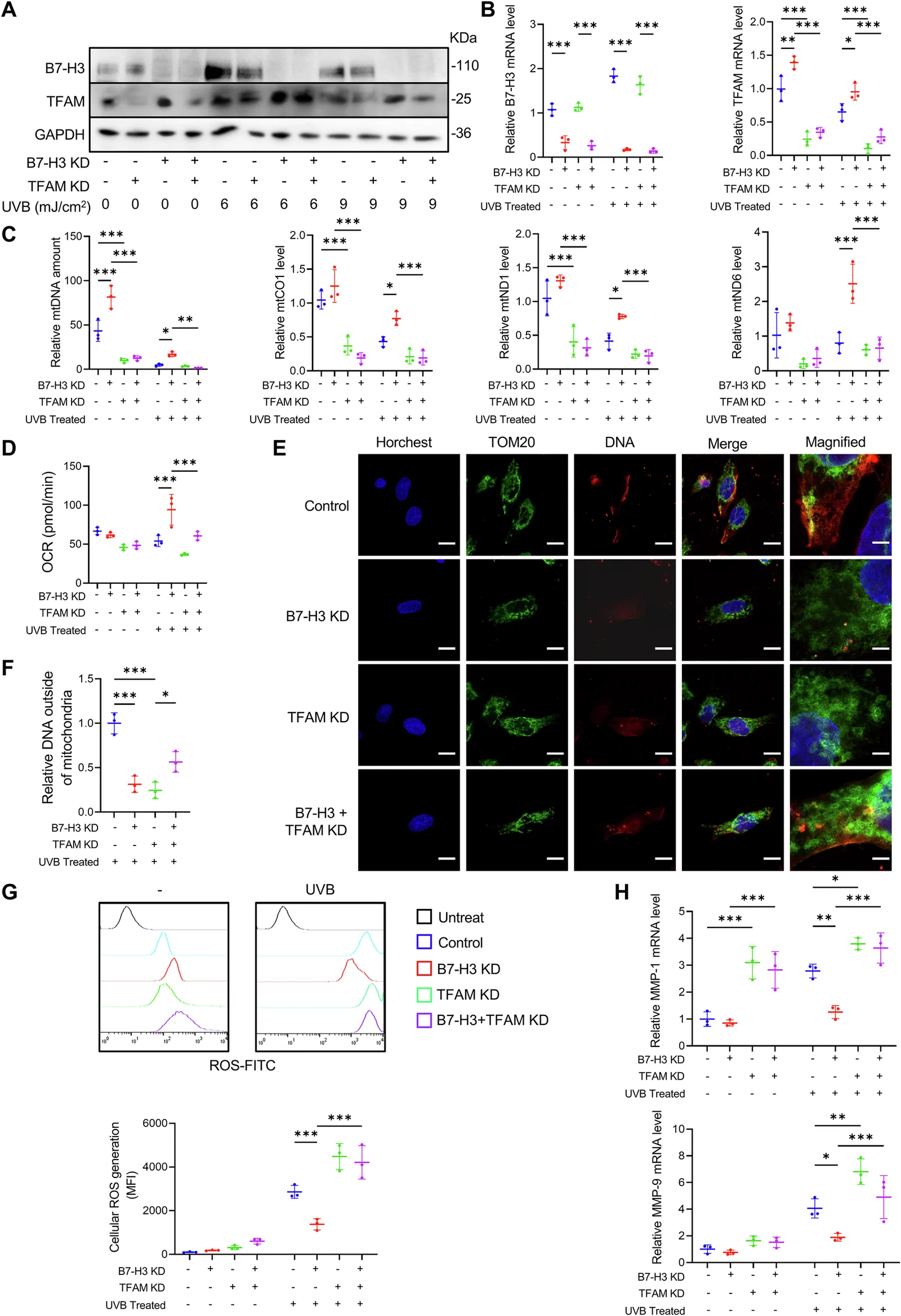
TFAM reduction reverses the protective effects that B7-H3 played in photodamage. Irradiation conditions: HaCaT cells were irradiated once with UVB at indicated doses and harvested 24 h post-irradiation. For dose-response experiments, cells were exposed to 0, 6, or 9 mJ/cm^2^ UVB (**A**). For all functional assays, a moderate dose of 9 mJ/cm^2^ UVB was used to induce stable photodamage responses while maintaining sufficient cell viability for mitochondrial function assessment (**B**–**H**). **A** Western blot analysis of B7-H3 and TFAM in HaCaT cells treated with UVB. **B** Relative mRNA levels of *B7-H3* and *TFAM* in HaCaT cells treated with 9 mJ/cm^2^ UVB. **C** Relative mtDNA, and mRNA levels of mtDNA-encoded complexes subunits in HaCaT cells. **D** OCR measured with phosphorescence-based detection. **E**, **F** IF detection of relative cytosolic mtDNA leakage in HaCaT cells. Scale bars: 10 μm (Horchest, TOM20, DNA, Merge) and 2 μm (Magnified) as indicated in the figure. **G** FACS analysis of intracellular ROS using DCFH-DA probe in HaCaT cells. **H** Relative mRNA levels of *MMP-1* and *MMP-9* in HaCaT cells. *n* = 3 independent experiments. * represents *P* < 0.05; ** represents *P* < 0.01; *** represents *P* < 0.001. Error bars represent mean ± SD.

To determine whether TFAM mediates B7-H3-dependent mitochondrial dysfunction under UVR stress, we conducted TFAM knockdown via small interfering RNA (siRNA) in B7-H3 KD cells followed by UVB irradiation. Successful knockdown of B7-H3 and TFAM was confirmed at both the mRNA and protein levels via qRT-PCR and western blot (Fig. 5A, B). TFAM inhibition abolished the mitochondrial protective effects conferred by B7-H3 knockdown. Specifically, UVB-induced repression of mtDNA-encoded respiratory genes *CO1*, *ND1*, and *ND6* was reversed by B7-H3 depletion but subsequently counteracted when TFAM was also inhibited. These effects were consistent with reduction in mtDNA copy number and transcript abundance, underscoring the central role of TFAM in maintaining mitochondrial gene expression (Fig. 5C). Functionally, B7-H3 knockdown restored OXPHOS capacity under UVB stress, as evidenced by enhanced oxygen consumption rate (OCR). However, this improvement was completely abolished upon TFAM inhibition, confirming its essential role downstream of B7-H3 (Fig. 5D). B7-H3 knockdown also reduced UVB-induced cytoplasmic extrusion of mtDNA—a hallmark of mitochondrial membrane rupture. This protective effect remained intact even with TFAM knockdown (Fig. 5E, F). This apparent paradox may reflect TFAM’s dual role: while B7-H3 inhibition stabilizes mitochondrial membranes and prevents mtDNA leakage, TFAM is responsible for mtDNA replication and compaction. Hence, its silencing may block mitochondrial genome recovery without disrupting membrane stabilization.

At the level of redox regulation, ROS levels were significantly reduced in B7-H3 KD cells but were partially restored by concomitant TFAM knockdown (Fig. 5G). Similarly, B7-H3 knockdown attenuated UVB-induced expression of photodamage markers *MMP-1* and *MMP-9*, while TFAM inhibition reversed this effect (Fig. 5H). Collectively, these findings demonstrate that B7-H3 promotes mitochondrial dysfunction, oxidative stress, and photodamage by suppressing TFAM. Restoring TFAM function is therefore essential for mitigating the deleterious effects of UVB in a B7-H3-dependent manner.

### NRF1 and NRF2 levels are negatively correlated with B7-H3 expression in photodamaged skin

**Fig. 6:**
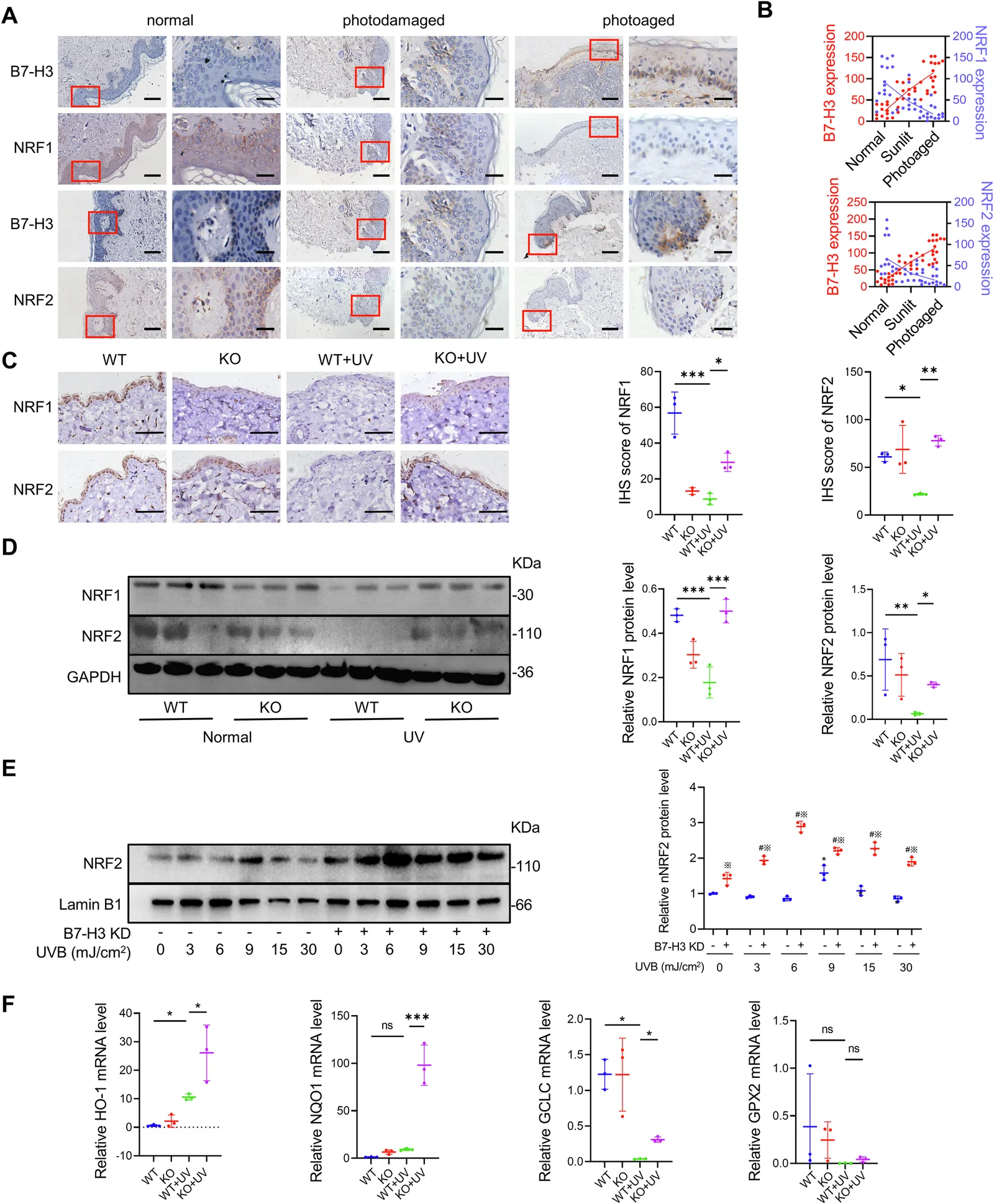
NRF1, NRF2 levels are negatively correlated with B7-H3 expression in photodamaged skin. Irradiation conditions: For in vivo experiments, mice were exposed to UVB irradiation (300 mJ/cm^2^) daily for three consecutive days and skin samples were collected 24 h after the final exposure (**C**, **D**, **F**). For in vitro experiments, HaCaT cells were irradiated once with UVB at indicated doses (0, 3, 6, 9, 15, 30 mJ/cm^2^) and nuclear fractions were prepared 24 h post-irradiation (**E**). **A**, **B** IHC staining of NRF1, NRF2, and B7-H3 in normal, photodamaged and photoaged human skin. Scale bars: 100 μm (main images) and 50 μm (enlarged views) as indicated in the figure. IHC staining (**C**) and western blot analysis (**D**) of NRF1 and NRF2 in mouse skin tissues under UVR. Scale bars: 100 μm as indicated in the figure. **E** Western blot analysis to detect NRF2 levels in the nucleus of HaCaT cells. **F** qRT-PCR analysis of *HO-1*, *NQO1*, *GCLC*, and *GPX2* mRNA levels in mouse skin tissues. *n* = 3 female mice or *n* = 3 independent experiments. C, D, F: * represents *P* < 0.05; ** represents *P* < 0.01; *** represents *P* < 0. 001. **E** * *P* < 0.05 (Control + UVR vs Control); # *P* < 0.05 (B7-H3 KD + UVR vs B7-H3 KD); ※ *P* < 0.05 (B7-H3 KD + UVR vs Control + UVR, at the same dose). Error bars represent mean ± SD.

Previous studies indicate that nuclear translocation of NRF2 is crucial for the activation of antioxidant enzymes in cancer cells^20^. NRF1 promotes mitochondrial biogenesis by positively regulating TFAM expression and is regulated by NRF2 to maintain redox homeostasis^12,21,22^. Given the established roles of the NRF1 and NRF2 in regulating mitochondrial function and oxidative stress, we next investigated their expression in human photodamaged skin. We observed a marked reduction in NRF1 and NRF2 expression in photodamaged skin tissues, which inversely correlated with elevated B7-H3 levels at the same tissue sites (Fig. 6A, B). In UVB-irradiated HaCaT cells, NRF1 and NRF2 initially exhibited transient upregulation, likely as part of a compensatory antioxidant response, but were subsequently suppressed with prolonged UVR exposure (Supplementary Fig. 5A, B). Critically, knockdown of B7-H3 significantly restored NRF1 and NRF2 expression in both UVR-exposed skin tissues and HaCaT cells (Fig. 6C, D, Supplementary Fig. 5A, B), indicating that B7-H3 exerts a repressive effect on these key antioxidant regulators. To further determine the mechanistic relationship, we performed nuclear protein isolation followed by western blot analysis in HaCaT cells. Results confirmed that B7-H3 inhibited UVR-induced nuclear translocation of NRF2, suggesting that B7-H3 may inhibit NRF2 activation under oxidative stress (Fig. 6E). Given that NRF1 is a known positive regulator of TFAM and that NRF2 can indirectly influence this pathway, our data suggest that B7-H3 may disrupt the NRF2/NRF1-TFAM pathway, providing a potential mechanism for the observed mitochondrial dysfunction and oxidative damage in photodamaged skin. To further assess whether the reduced NRF2 nuclear translocation translates into impaired transcriptional activity, we examined the mRNA levels of canonical NRF2 target genes in UVB-irradiated mouse skin samples. We observed two distinct patterns of regulation (Fig. 6F). *Ho-1* (heme oxygenase-1) and *Nqo1* (NAD(P)H: quinone oxidoreductase 1), which are classic rapid-response NRF2 targets, were induced upon UVB exposure; this induction was further enhanced in B7-H3 KO mice. In contrast, *Gclc* (glutamate-cysteine ligase catalytic subunit) and *Gpx2* (glutathione peroxidase 2), which encode key enzymes for glutathione synthesis and utilization, showed decreased expression following UVB irradiation. For *Gclc*, this reduction was significantly rescued in B7-H3 KO mice. For *Gpx2*, a similar trend toward restoration was observed, though it did not reach statistical significance. Despite the distinct regulatory patterns among these target genes, a difference that likely reflects differential promoter sensitivity, feedback adaptation, or the balance between stress induction and metabolic exhaustion, the consistent finding that B7-H3 depletion either increased or restored their expression collectively indicates that B7-H3 suppresses NRF2-dependent antioxidant gene expression. Together with the observed inhibition of NRF2 nuclear translocation, these data provide functional evidence that B7-H3 impairs NRF2 transcriptional activity under UVR-induced oxidative stress.

### B7-H3/ITGA2-NRF2 axis disrupts mitochondrial homeostasis in photodamage

**Fig. 7:**
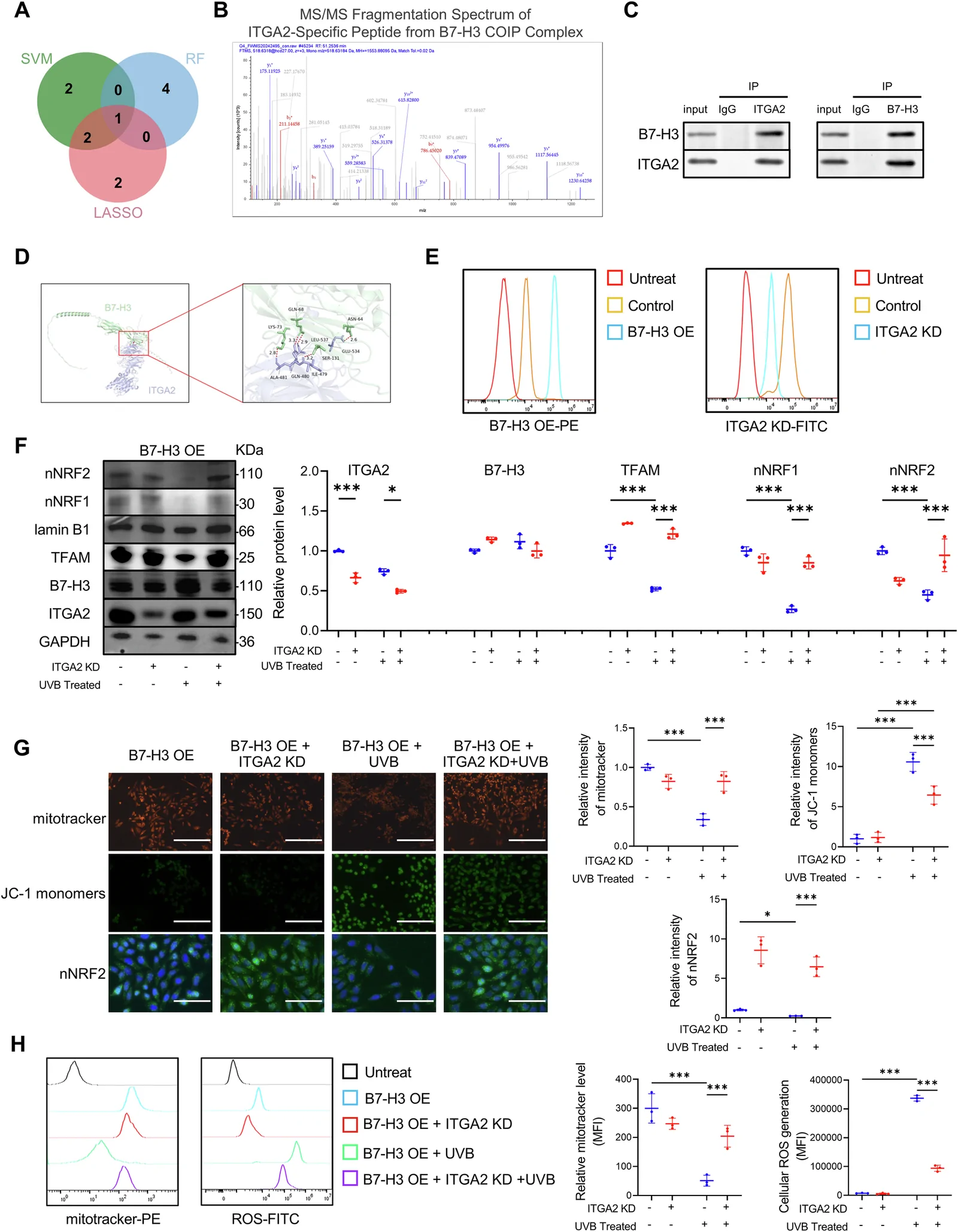
B7-H3 interacts with ITGA2 to disrupt mitochondrial homeostasis in photodamage. Irradiation conditions: For functional assays in HaCaT cells (**F**–**H**), cells were irradiated once with UVB (9 mJ/cm^2^) and harvested 24 h post-irradiation. **A** Venn diagram of B7-H3 interactors prioritized by machine learning for skin aging association. **B** Tandem MS profile of characteristic peaks from B7-H3/ITGA2 Co-IP complex (Sequence: IPLLYDAEIHLTR, Charge: +3, m/z: 518.63, XCorr: 2.58, PEP: 1.6 × 10^–8^). **C** Experimental validation of B7-H3/ITGA2 interaction by Co-IP in HaCaT cells. **D** Predicted B7-H3 /ITGA2 interaction interface via AlphaFold structural modeling and PyMOL-based docking simulations. **E** FACS analysis of ITGA2 KD in B7-H3 OE HaCaT cells. **F** The protein levels of total TFAM and nuclear-localized NRF1/NRF2 in ITGA2 KD and B7-H3 OE HaCaT cells. **G**, **H** ITGA2 knockdown restored mitochondrial homeostasis under UVB stress: enhanced mitotracker fluorescence, reduced JC-1 monomer formation, and attenuated ROS accumulation. Scale bars: 200 μm (mitotracker, JC-1 monomers) and 100 μm (nNRF2) as indicated in the figure. *n* = 3 independent experiments. * represents *P* < 0.05; ** represents *P* < 0.01; *** represents *P* < 0.001. Error bars represent mean ± SD.

To elucidate the molecular mechanisms underlying B7-H3’s role in photodamage, we performed immunoprecipitation coupled with mass spectrometry (IP/MS) in HaCaT cells, and identified 2232 putative B7-H3-interacting proteins including several integrin family members (Supplementary Fig. 6A). Given B7-H3’s transmembrane nature, we prioritized membrane-associated effectors. To narrow down candidates with relevance to skin aging, we integrated our interactome data with the GSE75192 aging transcriptomic dataset^23^. By applying machine learning-based feature selection algorithms—Support Vector Machine-Recursive Feature Elimination (SVM-RFE), Random Forest (RF), and Least Absolute Shrinkage and Selection Operator (LASSO), we identified aging-associated interactors within skin tissues (Supplementary Fig. 6B–D). Cross-method intersection analysis pinpointed ITGA2 as the top-ranked candidate, with other integrins like ITGB1 also enriched but ranking lower (Fig. 7A, Supplementary Fig. 6E). ITGA2 was identified as a potential B7-H3-associated interactor supported by IP/MS and Co-IP (Fig. 7B, C). Protein docking simulations using AlphaFold and PyMOL further predicted a plausible binding interface between B7-H3 and ITGA2 (Fig. 7D).

Functionally, ITGA2 knockdown in B7-H3-overexpressing (OE) HaCaT cells restored mitochondrial homeostasis. Specifically, depletion of ITGA2 enhanced nuclear protein levels of both NRF1 and NRF2, as well as increased total TFAM protein expression (Fig. 7E, F). ITGA2 silencing also mitigated UVB-induced mitochondrial dysfunction, as evidenced by restored mitotracker fluorescence, reduced JC-1 monomer formation (indicative of preserved mitochondrial membrane potential), and decreased ROS levels (Fig. 7G, H). Collectively, these results suggest that the B7-H3/ITGA2 interaction acts upstream of the NRF2/NRF1-TFAM pathway and contributes to mitochondrial dysfunction during photodamage. This signaling mechanism provides mechanistic insight into how B7-H3 disrupts redox and metabolic homeostasis in the skin under UVR stress.

### Therapeutic efficacy of anti-B7-H3 monoclonal antibody (mAb) in skin photodamage

**Fig. 8:**
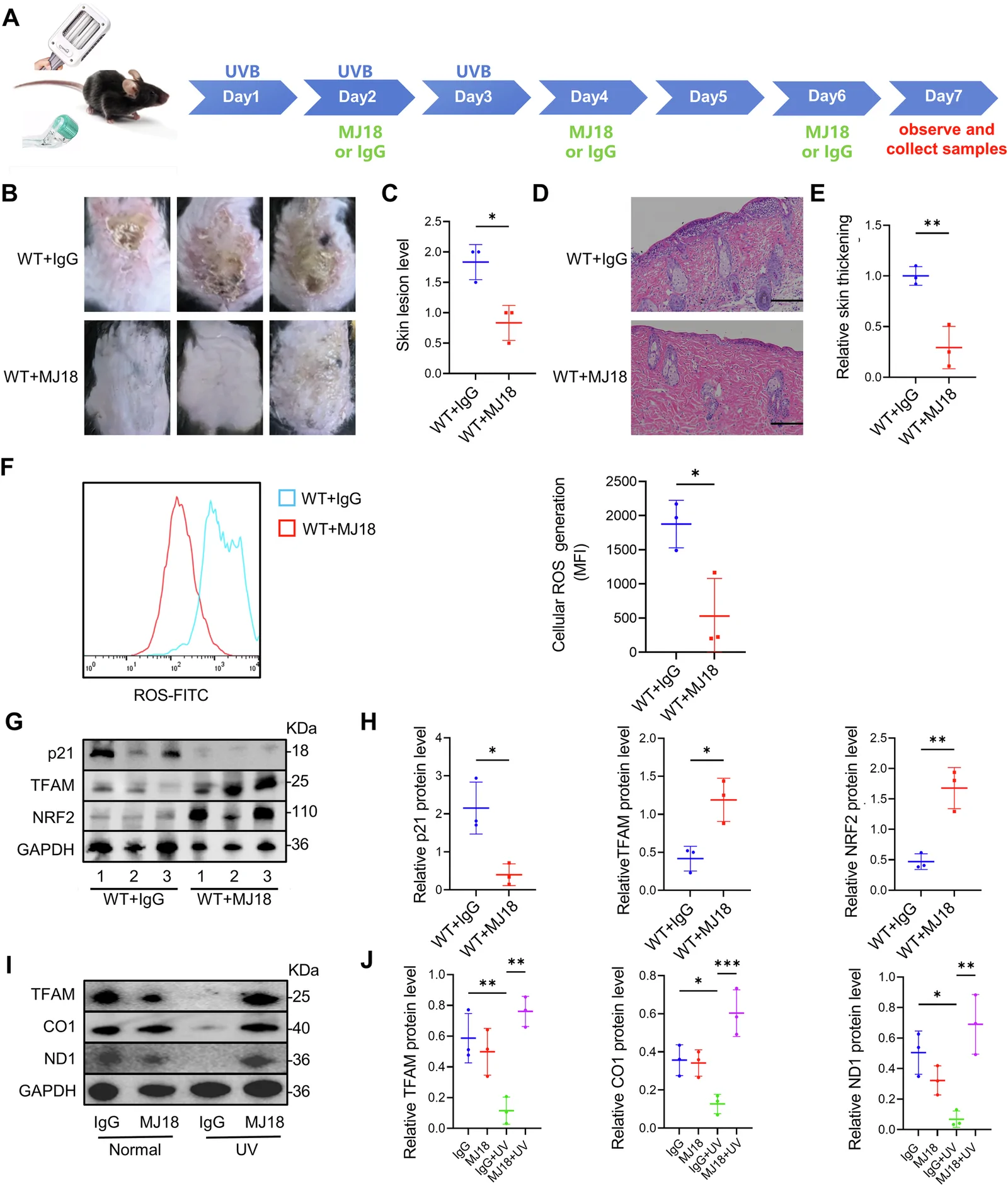
Efficacy of B7-H3 blockade in skin photodamage. **A** Schematic diagram of UVB irradiation and MJ18/control IgG injection protocol. Mice received UVB irradiation (300 mJ/cm^2^) on days 1-3, MJ18 or control IgG administration on days 2, 4, and 6, and were sacrificed on day 7 for sample collection. The optical images (**B**) and photodamage scores (**C**) of mouse skin treated with MJ18. H&E staining images (**D**) and relative skin thickening (**E**) of mouse skin with different treatments. Scale bars: 100 μm as indicated in the figure. **F** FACS analysis of ROS in mouse skin tissues treated with MJ18. Western blot analysis (**G**) and quantitative results (**H**) of p21 and mitochondria-related proteins in mouse skin tissues. **I**, **J** Primary mouse skin fibroblasts were irradiated with UVA (10 J/cm^2^) and harvested 24 h post-irradiation. Protein levels of B7-H3 and ETC-related genes in primary mouse skin fibroblasts treated with MJ18 or IgG. *n* = 3 female mice or *n* = 3 independent experiments. * represents *P* < 0.05; ** represents *P* < 0.01; *** represents *P* < 0.001. Error bars represent mean ± SD.

To evaluate the potential clinical applications of targeting the B7-H3 pathway, we assessed the therapeutic efficacy of an anti-B7-H3 blocking mAb (MJ18) against skin photodamage compared to control rat IgG (Fig. 8A). Administration of MJ18 via needle rolling markedly reduced skin erythema, edema, and other hallmarks of photodamage, while enhancing antioxidant capacity in vivo (Fig. 8B–F). MJ18 treatment downregulated p21 expression and restored TFAM and NRF2 levels in skin tissues (Fig. 8G, H). Mechanistically, MJ18 enhanced TFAM and ETC-related protein expression in mouse primary fibroblasts (Fig. 8I, J). Upon treatment with MJ18, the expression levels of CD8^+^ cells were found to be exceedingly low and remained virtually unchanged (Supplementary Fig. 7). Collectively, these results demonstrate that targeting B7-H3 with a blocking mAb alleviates skin photodamage by restoring mitochondrial and antioxidant functions, highlighting its promising therapeutic potential.

## Discussion

Our investigation identified that B7-H3 was aberrantly overexpressed in photodamaged skin and revealed a role not previously reported for the B7-H3/ITGA2 axis in promoting mitochondrial dysfunction and oxidative stress. Specifically, UVR induced cooperative inhibition of both TFAM expression and NRF2 signaling, and our data indicated that the interaction between B7-H3 and ITGA2 contributed to these effects, accompanied by a cascade of redox imbalance characterized by ROS accumulation and mitochondrial impairment. This cascade is associated with the development of cutaneous photodamage. Consistent with these mechanistic findings, treatment with anti-B7-H3 blocking antibodies attenuated UVR-induced skin injury, suggesting that B7-H3 targeting warrants further investigation as a potential strategy to restore mitochondrial redox homeostasis.

While B7-H3 is well known as an immune checkpoint molecule implicated in tumor immune evasion, accumulating evidence has expanded its functional repertoire to include immune-independent roles such as regulation of metabolism, cell cycle progression, and epithelial-mesenchymal transition^24,25^. Our prior work implicated B7-H3 in the development of CSCC, prompting further investigation into its expression in photoaged skin. We now show that B7-H3 is highly expressed in both the epidermis and dermis of photodamaged human skin and is upregulated by UVR in a dose-dependent manner. The coordinated elevation of B7-H3 in both epithelial and stromal compartments suggests that it may serve as a conserved mediator of photodamage across multiple skin cell types.

Interestingly, B7-H3 upregulation was observed at early stages of UVR exposure but declined with prolonged or high-intensity irradiation in vitro. This temporal pattern may reflect the transition from a reparative to a decompensated state, wherein the cellular damage surpasses the capacity of skin cells to respond (Supplementary Fig. 1F, G). These data support a model in which B7-H3 functions as a stress-induced regulator that amplifies photodamage under sublethal UVR stress but may become suppressed once cellular systems collapse under sustained injury. Excessive ROS production plays a central role in UVR-induced skin injury by activating adenosine monophosphate-activated protein kinase (AMPK) signaling, enforcing cell cycle arrest, and driving MMP-mediated matrix degradation through extracellular signal-regulated kinase (ERK) and nuclear factor kappa-light-chain-enhancer of activated B cells (NF-κB) pathways^26,27^. Prior studies have reported that B7-H3 reduces antioxidant defenses, including SOD1, SOD2, and peroxiredoxin 3, thereby enhancing oxidative stress and supporting hypoxia-inducible factor 1 alpha (HIF-1α)-mediated metabolic reprogramming in cancer^20^. In line with these findings, we demonstrated that B7-H3 promoted early ROS accumulation and suppressed antioxidant enzyme expression following UVR exposure. Notably, this effect was evident only under UVB-induced stress and not in baseline conditions, suggesting that B7-H3 acts as a stress amplifier rather than a constitutive ROS generator. This stress-dependent ROS amplification may contribute to the accumulation of cellular damage that, over repeated exposures, could promote long-term photodamage and increase skin cancer risk, as suggested by previous studies linking oxidative stress to photoaging and carcinogenesis^6,28^. Our findings position B7-H3 as a central link between immune regulation, mitochondrial dysfunction, and redox imbalance in the skin’s response to environmental stress.

TFAM inactivation was shown to be accompanied by elevated production of ROS and reduced cell proliferation^29^. TFAM is one of the most abundant mitochondria-localized DNA-binding proteins, which is related to mtDNA replication, transcription and packaging^11,19^. The nucleoid structure formed by TFAM and mtDNA can participate in the removal of mitochondrial oxidative stress and prevent excessive mitochondrial damage in both sequence-specific and non-specific manners^30^. Germline deletion of TFAM is embryonically lethal due to catastrophic loss of mtDNA, and tissue-specific TFAM deletion leads to impaired OXPHOS across diverse cell types, reproducing features of mitochondrial disease in mice and humans^31,32^. Knockout of TFAM in epidermal-specific flox mice promotes primary skin tumorigenesis by impairing dihydroorotate dehydrogenase (DHODH), a key regulator of ETC integrity^33^. Also, overexpression of TFAM can reverse mitochondrial damage, reduce superoxide accumulation, and improve the activity of ETC^19,34^. In immune cells, TFAM deficiency leads to premature senescence and dysfunction, highlighting its broader role in maintaining mitochondrial and cellular integrity^31^. B7-H3 KO mice exhibited reduced OCR and a modest increase in extracellular acidification rate (ECAR), indicating that B7-H3 is involved in mitochondrial metabolic reprogramming^35^. Our findings suggest that B7-H3 negatively regulates TFAM expression, positioning it as a key upstream modulator whose suppression amplifies UVR-induced mitochondrial dysfunction. This highlights a critical checkpoint in redox regulation and points to potential therapeutic opportunities for TFAM-related oxidative pathologies. However, whether B7-H3 directly represses TFAM transcription or acts through indirect mechanisms remains to be determined and warrants further investigation.

Consistent with prior reports in cancer metabolism, where B7-H3 promotes ROS-dependent HIF-1α stabilization by suppressing NRF2 nuclear translocation and its downstream antioxidants, our data suggest that B7-H3 is associated with impaired NRF2 nuclear translocation in photodamaged skin^20^. In UVB-irradiated skin, B7-H3 knockout enhanced the induction of *Ho-1* and *Nqo1* and restored the UVB-suppressed expression of *Gclc*, whereas *Gpx2* displayed a similar trend toward restoration that did not reach statistical significance. Although these target genes exhibited distinct regulatory patterns, their consistent increase or restoration upon B7-H3 depletion supports that B7-H3 suppresses NRF2 transcriptional activity, thereby diminishing ROS scavenging capacity and promoting oxidative stress^36,37^. Critically, our study also demonstrated that B7-H3 negatively influenced NRF1 expression, a key transcription factor that binds the *TFAM* promoter and governs mitochondrial biogenesis^37,38^. Given that NRF2 activation induces *NRF1* expression, B7-H3-mediated NRF2 suppression likely cascades into impaired NRF1-TFAM signaling, leading to mtDNA instability and mitochondrial dysfunction^12,39,40^. Although our study did not directly validate TFAM as a downstream target of NRF1 in photodamage, the concordant reduction of NRF1, NRF2, and TFAM across in vivo and in vitro models, coupled with functional rescue upon ITGA2 knockdown in B7-H3 OE cells, supports a model in which the B7-H3/ITGA2 interaction is functionally linked to dysregulation of the NRF2/NRF1/TFAM cascade. This disruption contributes to mitochondrial redox failure and photodamage progression.

This study identifies ITGA2 as a binding partner of B7-H3, demonstrating that their interaction exacerbated oxidative stress by inducing mitochondrial dysfunction in UVR-induced skin photodamage. While integrin family members such as ITGA2 have been implicated in redox regulation, their mechanistic roles in cutaneous photodamage remain incompletely understood. Prior work has shown that ITGA2 contributes to aging by modulating ROS generation, mitochondrial dysfunction, and apoptosis^41^. In addition, ITGA2 can promote collagen degradation by enhancing MMP expression via inflammatory signaling^42,43^. Notably, as key mediators of cell adhesion and microenvironment sensing, integrins functionally extend beyond mechanotransduction to influence cellular fate by regulating metabolic reprogramming and mitochondrial homeostasis^44,45^. For example, ITGA2 has been shown to amplify mitochondrial ROS accumulation through collagen-dependent signaling, driving metabolic dysfunction^43^. Our findings revealed that the B7-H3/ITGA2 axis was associated with compromised mitochondrial function and reduced TFAM expression, likely through disruption of NRF2/NRF1-mediated mitochondrial biogenesis signaling. Although direct regulation of NRF2 or TFAM by ITGA2 has not been previously reported, our integrated bioinformatic and functional analysis suggests that the B7-H3/ITGA2 interaction may indirectly modulate NRF2 activity through canonical integrin effectors such as FAK or PI3K signaling. These observations raise the possibility that this interaction could contribute to a self-reinforcing cycle involving oxidative stress, mitochondrial damage, matrix remodeling, and senescence amplification, a concept that has been proposed in the context of photoaging^7,46,47^. However, given that our data are derived from acute UVR exposure models, further long-term studies are needed to directly establish such a cycle in chronic photodamage. This proposed regulatory axis, if validated in future long-term studies, would expand the current understanding of integrins as active participants in photodamage beyond their conventional roles.

Nonetheless, the precise molecular details by which B7-H3/ITGA2 binding regulates the NRF2-TFAM axis remain to be defined. Specifically, it is unclear whether B7-H3 alters ITGA2 phosphorylation, subcellular localization, or affinity for cytosolic adaptor proteins. Furthermore, B7-H3 may cooperatively regulate photodamage by forming complexes with other integrins (e.g., ITGB1), while the specific contribution of ITGA2 requires further validation through conditional knockout or pharmacological inhibition studies. From a broader perspective, the B7-H3/ITGA2 interaction bridges immune regulatory molecules with cell adhesion signaling, highlighting the central role of fundamental cellular processes (adhesion, metabolism) in photodamage pathogenesis. The low basal expression of B7-H3 in normal skin, contrasted with its marked upregulation upon UVR exposure, suggests it may act as an environmental stress sensor that reprograms cellular metabolism via integrin-mediated signaling, ultimately promoting aging-associated phenotypes. This discovery not only expands the biological significance of B7-H3 beyond immunoregulation but also provides a rationale for targeting the B7-H3-integrin interaction in therapeutic strategies against photodamage.

To assess the effects of B7-H3 blockade in skin photodamage, we utilized MJ18, a commercially available and well-characterized function-blocking mAb that specifically recognizes mouse B7-H3 and has been shown to disrupt its interaction with potential receptors in multiple previous studies^48,49^. Subsequently, this antibody has been employed as a validated tool for functional B7-H3 blockade in various disease models, including asthma and cancer^50^. In our study, MJ18 treatment restored TFAM and ETC-related protein expression in the skin and primary fibroblasts of UVR-exposed mice, reducing oxidative stress and mitigating tissue injury. These findings suggest that MJ18 interferes with endogenous B7-H3-driven signaling cascades. Given that MJ18 was originally characterized in studies where it modulated T cell responses, we considered whether its protective effects in our model might be mediated through T cells. To explore this, we examined T cell infiltration in the skin. T cell numbers were low across all groups and did not differ significantly between treatment conditions. While this observation does not exclude the possibility that resident or transient immune cells contribute to the response, it provides no direct support for an immune-mediated mechanism. More importantly, MJ18 retained its efficacy in cultured primary fibroblasts, a system devoid of T cells, demonstrating that the antibody can act directly on non-immune cells. These findings suggest that while MJ18 can modulate T cell function in immune contexts, its activity in our model reflects a direct effect on skin cells. This interpretation is further supported by the consistency between the effects of MJ18 treatment and those observed in B7-H3 KO mice and B7-H3 KD cells, including restored TFAM expression, reduced ROS accumulation, and attenuated photodamage. The concordance of these phenotypes provides functional evidence that MJ18 targets the same B7-H3-driven pathway identified throughout this study. Given the well-documented specificity of MJ18 as a validated B7-H3-blocking antibody in the literature, these convergent data from multiple loss-of-function models collectively support the conclusion that the observed protective effects are attributable to B7-H3 blockade rather than off-target antibody effects.

Nevertheless, we acknowledge that direct biochemical evidence for MJ18 binding to B7-H3 is not provided here. The precise molecular mechanism remains to be fully defined, particularly whether MJ18 specifically blocks the B7-H3/ITGA2 interaction identified in this study or acts through other B7-H3-associated proteins such as IL20RA, MVP, or Fn, which have been shown to mediate diverse functions in tumor biology independent of adaptive immunity^51–53^. In addition, the use of a micro-needle roller for local antibody delivery may introduce confounding factors such as needle-induced inflammation or wound healing responses, which could influence the observed therapeutic effects independently of B7-H3 blockade. Although microneedling is a well-established technique in dermatology for enhancing transdermal drug delivery and clinical studies indicate that its primary role is to facilitate drug penetration rather than to confer direct therapeutic benefit, we cannot completely exclude the possibility that the micro-needling procedure itself contributed to the observed effects. Extending beyond its well-characterized role in tumor biology, our findings suggest a role for B7-H3 in non-tumor pathologies, indicating a broader functional paradigm for B7-H3 beyond oncological contexts. Therefore, further studies with validated target specificity and improved delivery methods are warranted to confirm the therapeutic potential of B7-H3 targeting. Taken together, these data support the feasibility of targeting B7-H3 for photodamage intervention. Our mechanistic exploration using genome-wide B7-H3 KO models has delineated its photoprotective axis, though cell-type-specific functional dissection through conditional knockout (CKO) approaches would add another layer of precision to these findings.

In summary, this study identifies a role not previously reported of B7-H3 in the context of UVR-induced photodamage. B7-H3 is associated with disrupted mitochondrial homeostasis and reduced TFAM-dependent biogenesis, promoting ROS accumulation and oxidative stress, while ITGA2 signaling potentially contributes to this effect. These findings not only expand the non-immune roles of B7-H3 in stress sensing and metabolic reprogramming but also identify the B7-H3-TFAM signaling cascade as a potential therapeutic target for mitigating skin photodamage.

## Methods

### Antibodies

Anti-human 4IgB7-H3 antibody (R&D Systems, USA, AF1027, 1:200 for IHC; Proteintech, China, 14453-1-AP, 1:5000 for western blot); anti-mouse B7-H3 antibody (Santa Cruz, USA, sc-376769, 1:5000 for western blot, 1:200 for IHC); anti-human p21 antibody (Cell Signaling Technology, USA, 2947S, 1:1000 for western blot); anti-mouse p21 antibody (Proteintech, China, 28248-1-AP, 1:1000 for western blot, 1:200 for IHC); anti-human p53 antibody (Cell Signaling Technology, USA, 9282S, 1:1000 for western blot); anti-mouse COL1A1 antibody (Proteintech, China, 66761-1-Ig, 1:100 for IHC); anti-mouse COL3A1 antibody (Proteintech, China, 22734-1-AP, 1:100 for IHC); anti-human TFAM antibody (Proteintech, China, 22586-1-AP, 1:5000 for western blot, 1:100 for IHC, 1:200 for immunofluorescence); anti-mouse TFAM antibody (Santa Cruz, USA, sc-166965, 1:1000 for western blot, 1:100 for IHC); anti-human/mouse NRF1 antibody (Santa Cruz, USA, sc-28379, 1:1000 for western blot, 1:50 for IHC, 1:50 for immunofluorescence); anti-human NRF2 antibody (Santa Cruz, USA, sc-365949, 1:1000 for western blot, 1:50 for IHC, 1:100 for immunofluorescence); anti-mouse NRF2 (Proteintech, China, 16396-1-AP); anti-human/mouse CO1 antibody (Invitrogen, USA, 1D6E1A8, 1:1000 for western blot, 1:50 for IHC); anti-human/mouse ND1 antibody (Proteintech, China, 19703-1-AP, 1:1000 for western blot, 1:100 for IHC); anti-human/mouse ITGA2 antibody (Abcam, USA, ab181548, 1:10000 for western blot, 1:500 for IHC), anti-human TOMM20 antibody (Abcam, USA, ab186735, 1:250 for immunofluorescence), anti-human DNA antibody (AC-30-10, Germany, Progen, 1:200 for immunofluorescence).

### Patients’ information

In our study, 50 patients were enrolled, including 27 males and 23 females, aged 27 to 71 years, with an average age of 42.5 years old. All skin samples were surgically resected from patients who went to Department of Dermatology, the First Affiliated Hospital of Soochow University, Soochow, China for excision of skin lesions, then fixed with neutral buffered formalin and embedded in paraffin. Based on clinical diagnosis and histopathological evaluation by two qualified dermatologists, the samples were classified into four groups: normal skin (*n* = 15, obtained from sun-protected areas such as post-auricular and upper inner arm regions of young to middle-aged donors with no history of significant sun exposure and no clinical signs of photoaging, mean age 32.9 ± 5.6 years); naturally aged skin (*n* = 8, obtained from sun-protected areas of donors presenting with typical senile skin changes including fine wrinkling and laxity attributable to chronological aging, but lacking the characteristic features of photoaging, mean age 57.3 ± 7.9 years); photodamaged skin (*n* = 12, obtained from sun-exposed areas such as face and forearm of donors who, despite regular sunlight exposure, did not exhibit severe clinical signs of photoaging such as deep wrinkles or leathery appearance, mean age 33.8 ± 6.4 years); and photoaged skin (*n* = 15, obtained from severely sun-exposed areas such as face of donors presenting with classic clinical features of photoaging, including deep wrinkles, leathery appearance, and pigmentary irregularities, mean age 51.3 ± 10.1 years). The clinical data of each patient included sex, age, occupation, surgical site and photodamaged severity, which was evaluated by two qualified dermatologists. This study was approved by the clinical research ethics committee of the First Affiliated Hospital of Soochow University (approval number: 2019251).

### The establishment and identification of B7-H3 KO mice

The following mouse lines were used in this study:

CD276 flox mice (full official name: C57BL/6 JGpt-*Cd276*^em1Cflox^/Gpt, abbreviated as *CD276* flox in the manuscript). These mice were generated by GemPharmatech Co., Ltd. (Strain No. T018090; MGI: 2183926) on a C57BL/6JGpt background. As this line was custom-developed by GemPharmatech, detailed information including characterization and genotyping validation, can be found on the GemPharmatech website.

*TGTN* mice (full official name: C57BL/6JGpt-*TgTn*(pb-CAG-iCre)1/Gpt, abbreviated as *TGTN* mice). These mice were generated by GemPharmatech Co., Ltd. (Strain No. T050269) on a C57BL/6JGpt background. As this line was custom-developed by GemPharmatech, detailed information including characterization and genotyping validation, can be found on the GemPharmatech website. The Cre recombinase is transmitted from both paternal and maternal lineages.

WT mice (full official name: C57BL/6JGpt, abbreviated as C57BL/6J or WT in the manuscript). These mice were purchased from GemPharmatech Co., Ltd. (Strain No. N000013) and maintained on the same C57BL/6JGpt background.

The construction strategy for the B7-H3 KO mice is presented in Supplementary Fig. 2A. Customized *CD276* flox mice were mated with *TGTN* mice in a specific pathogen-free (SPF) environment. At 8 weeks of age, the mice were mated in equal proportions to yield the first generation with a null/wtCreT genotype. These were then crossed with wt mice to produce heterozygous offspring without the Cre recombinase enzyme (null/wtCreW). Interbreeding these heterozygotes resulted in homozygous B7-H3 KO mice, which could be further propagated by mating homozygotes.

Genotyping was conducted using PCR on genomic DNA extracted from the tail tips of 4-week-old pups. The PCR amplification targeted specific fragments, and the products were resolved by agarose gel electrophoresis. The presence and size of these fragments were used to determine the genotype. Primers for genotype identification were used to verify B7-H3, with the wt positive band at 281 bp and the flox positive band at 381 bp. Primers used for null genotype identification had a positive band at 402 bp. Mice showing a 402 bp band with null primers and no amplification with B7-H3 primers were confirmed as B7-H3 KO. Sequences of primers:

B7-H3, AGAGCCTGTTACACTGCTGAGACCTGT, ATAGGAGCCCAAATCAGACGGCA.

null, AGAGCCTGTTACACTGCTGAGACCTGT, TCCCTTCCTATAGAATGGGGACAAGGA.

### Animal experimentation and evaluation of photodamage

Six- to eight-week-old female C57BL/6 J mice (Mus musculus) were obtained from the Jiangsu Institute of Clinical Immunology. After shaving the dorsal skin of mice with a blade, hair removal cream was applied again to ensure complete depilation. To model acute UVB-induced skin damage, mice were irradiated using a UVB phototherapy device (SH2-J/SC2-J, Sigma, Shanghai, China). The UVB intensity was calibrated to 1.0 mW/cm^2^. The UVB phototherapy device was placed in a dark room. Mice were individually placed in a custom-made box and were freely moving to expose the shaved dorsal skin. Mice were exposed to a daily UVB dose of 300 mJ/cm^2^ for three consecutive days, a well-established protocol for inducing acute photodamage^54,55^. The irradiation duration was calculated according to the formula: time (s) = dose (mJ/cm^2^) / intensity (mW/cm^2^). For antibody treatment experiments, mice were randomly divided into two groups using a random number table: UVB irradiation + control IgG (UVB + IgG) and UVB irradiation + anti-B7-H3 antibody (UVB + MJ18). To minimize potential confounders, all mice were housed under identical conditions, treatments were performed at the same time of day, and cage locations were balanced across groups. Following the same irradiation protocol (300 mJ/cm^2^ on days 1, 2, and 3), mice received subcutaneous injections of 100 μg of anti-mouse CD276/B7-H3 monoclonal antibody (clone MJ18, catalog number BE0124, BioXcell, USA) or control rat IgG (catalog number BE0290, BioXcell, USA) on days 2, 4, and 6 using a 200 μm micro-needle roller. The experimental timeline is illustrated in Fig. 8A. The injections were carefully performed to ensure consistent delivery of the antibody or control. Twenty-four hours after the final treatment, morphological changes in the dorsal skin of the mice were observed. The mice were euthanized by cervical dislocation, and skin tissue samples of approximately 1.5 cm × 1.5 cm were collected from both the non-depilated and UVB-exposed areas for subsequent experiments.

Photodamage scoring: Images of the dorsal skin of the mice were captured using a camera, and skin lesion severity was assessed according to established photodamage scoring criteria, which ranged from 0 to 2^54^. The scoring was performed by two independent investigators, and the average score was calculated for each mouse. 0: flesh-colored, smooth, and plump skin; 0.5: flesh-colored, slightly rough skin; 1: red patches with moderately rough skin and deepened skin lines; 1.5: red patches with noticeably rough skin, minor flaking, and some wrinkles; 2: a purple-red ulcerative condition with significantly rough and thickened skin, extensive flaking, and increased wrinkles.

Epidermal thickness measurement: H&E-stained skin sections were imaged under a light microscope (Olympus, Japan) at ×200 magnification. For each mouse, three non-overlapping high-power fields were randomly selected and photographed. Epidermal thickness was measured using ImageJ software. After calibrating the scale bar using the image scale, the full epidermal layer (from the top of the stratum corneum to the bottom of the basal layer) was measured at 5–10 randomly selected points per image, and the average value was calculated for that image. The epidermal thickness for each mouse was calculated as the average of three images. To minimize batch-to-batch staining variability and individual differences, data were normalized to the mean epidermal thickness of the WT control group and presented as relative epidermal thickness.

### Preparation of cell suspension and extraction of primary fibroblasts

Epidermis and dermis were separated using Dispase II. The dermal tissues were digested with a solution containing 1 mg/mL collagenase type I and 0.25% trypsin. The digestion mixtures were filtered through 70 μm cell strainers, centrifuged, and the pellets were resuspended to obtain cell suspension and primary dermal fibroblasts.

### Cell culture, UVR exposure, cell lentivirus and siRNA transfection

HSF and HaCaT (National Collection of Authenticated Cell Cultures, Chinese Academy of Sciences) were maintained in our laboratory and used in our study. HSF were cultured in DMEM media (Gibco, USA, 6125152), and HaCaT were cultured in DMEM and RPMI-1640 media (Gibco, USA, 11879020), containing 10% fetal bovine serum (FBS, Biological Industries, 04-001-1ACS) and 1% penicillin–streptomycin (Beyotime, China, C0222) at 37 °C in a humidified atmosphere of 5% CO_2_.

For UVR exposure experiments, cells were seeded in culture dishes and grown to 70-80% confluence prior to exposure. HaCaT cells were exposed to UVB using a UVB phototherapy device (SH2-J/SC2-J, Sigma, Shanghai, China) at indicated doses. HSF cells were exposed to UVA using a UVA light source (SH2A, Sigma, Shanghai, China). The culture medium was replaced with PBS during irradiation to avoid the generation of toxic photoproducts. After irradiation, PBS was removed and fresh culture medium was added, and cells were incubated for indicated time points before further analysis. Control cells were subjected to the same procedure without UVR exposure.

Lentiviral vector carrying human 4IgB7-H3 cDNA was generated by Abm (China), while an empty backbone vector was used as a control. The sequence is provided in Supplementary Data 1. For lentivirus infection, cells were grown to 30% confluence in 6-well plates and were infected with lentiviral particles at a multiplicity of infection (MOI) of 20. The infection efficiency was then confirmed by flow cytometry, qRT-PCR and western blot. For siRNA transfection, the siRNA targeting *TFAM* and *ITGA2* were purchased from GenePharma Co. Ltd. (China), and an empty backbone vector was used as a control. The siRNA sequences are provided in Supplementary Data 1. Cells were seeded into 6-well plates at a density of 1 × 10^5^ cells/well and then transfected with siRNA reagents using Lipofectamine 2000 (Invitrogen, USA, 11668019). Transfection efficiency was determined by qRT-PCR or western blot.

### UVR exposure of cells

Cells were incubated and washed with PBS, then irradiated in PBS through the plastic dish covered with a solar simulator. HSF cells were irradiated with 20, 40, 60 and 80 J/cm^2^ UVA. HaCaT cells were irradiated with 3, 6, 9, 15 and 30 mJ/cm^2^ UVB. The UVB irradiation intensity was calibrated to 1.0 mW/cm^2^. The exposure duration for each dose was calculated using the same formula: time (s) = dose (mJ/cm^2^) / intensity (mW/cm^2^). For UVA irradiation, the intensity was 10 mW/cm^2^, and the durations were calculated accordingly. After irradiation, cultures were given fresh medium.

### Immunohistochemistry

IHC was performed as the following steps. Specimens were paraffin-embedded and were cut into 4 μm sections for IHC detection. Sections with tissues were deparaffinized and rehydrated. After boiling in antigen retrieval with sodium citrate buffer (pH 6.0) for 3 min, the slides were immersed in hydrogen peroxide to quench endogenous peroxidase activity, and were then blocked for nonspecific binding by incubation in normal goat serum. The sections were subsequently incubated with primary antibody overnight at 4 °C and corresponding HRP-labeled secondary antibody. Next, the sections were visualized by staining with 3,3′-diaminobenzidine and counterstaining with hematoxylin. All sections were scanned with a DMetrix imaging system (DMetrix, USA) and measured using scoring criteria for immunostaining based on clinical data and adopting H-score.

### Immunofluorescence

The cells were first washed with phosphate-buffered saline (PBS, Solarbio, China, P1020), followed by fixation utilizing a 4% paraformaldehyde solution for 20 min. Permeabilization was achieved with a 0.1% Triton X-100 (Beyotime, China, P0096) in PBS solution for 15 min, which was then succeeded by sealing with a 3% bovine serum albumin (BSA, Fcmacs, China, FMS-WB021) in PBS solution for 30 min to prevent non-specific binding. The cells were then incubated with the primary antibody at 4 °C overnight. Subsequently, the application of a secondary antibody was conducted at room temperature for 30 min in a dark environment to preserve fluorescence integrity. Nuclear staining was performed with DAPI (SouthernBiotech, USA, 0100-20). Ultimately, the cells were examined under a scanning microscope, and the acquired images were analyzed and quantified employing ImageJ. For the confocal microscopic analysis (Leica, STELLARIS 5), the primary antibodies were visualized with secondary antibodies and XTSA®520 and XTSA®570 fluorescent dyes (Alphaxbio, China, AXT37025031). Hoechst (Solarbio, China, C0031) was used for nuclear counterstaining.

### Proliferation assay

Cell viability was assessed using the Cell Counting Kit-8 (CCK-8) assay (NCM Biotech, China, C6050) according to the manufacturer’s protocol. 1000 cells/well were seeded in 96-well plates and cultured in 100 μl of cell culture medium. After UVR exposure treatment and then cultured for 24, 48 and 72 h, the medium was replaced with an equal volume of conditioned media containing 10% CCK-8 reagent, and the cells were incubated for another 2 h. Cell proliferation was measured at a wavelength of 450 nm.

### Western blot analysis

Protein was extracted using 1% SDS lysis buffer (Beyotime, China, ST626), separated using the Nuclear and Cytoplasmic Protein Extraction Kit (Beyotime, China, P0027), and then separated by SDS-PAGE (Beyotime, China, P0012AC) and transferred to 0.45 µm PVDF membranes (GE Healthcare Life Science, Germany). The membranes were blocked with 5% BSA for 1 h and incubated with primary antibodies at 4 °C overnight. The membranes were incubated with the corresponding HRP-conjugated secondary antibodies (Beyotime, China, A0216/A0208, 1:5000) for 1 h at room temperature on the next day. And antigen-antibody reaction was visualized by ECL reagents (NCM Biotech, China, 10100) using a Chemi DocTM MP Imaging System (Bio-Rad, USA).

### Total RNA isolation and qRT-PCR assays

For analysis of individual genes, 1 μg of total RNA was used for DNA synthesis with SuperMix (Vazyme, Nanjing, China, R323), and qRT-PCR was conducted using a SYBR Green Master Mix (Vazyme, Nanjing, China, Q412) and performed on a CFX96 Touch^TM^ Real-Time PCR system (Bio-Rad, CA, USA). The PCR conditions were as follows: 95 °C for 5 min, and then 40 cycles of amplification for 10 s at 95 °C, 30 s at 60 °C, and next 95 °C for 15 s, 65 °C for 60 s, 95 °C for 15 s. Individual gene expression was normalized to β-actin mRNA. The relative expression of each targeted gene was analyzed using the 2^−ΔΔCt^ method. Sequences of primers:

*m B7-H3*, 5’ GGACCTACGTCCAGGGAACAT 3’, 5’ TGGTCACATTGCCAGTCAAGG 3’.

*m GAPDH*, 5’ TGGATTTGGACGCATTGGTC 3’, 5’ TTTGCACTGGTACGTGTTGAT 3’.

*m MMP-1*, 5’ GTTGGAGCAGGCAGGAAGG 3’, 5’ TAGCAGCCCAGAGAAGCAAC 3’.

*m MMP-9*, 5’ GTGAAGACGCAGAAGGTGGAT 3’, 5’ CGTAGCCCACATAGTCCACCT 3’.

*m HO-1*, 5’ TGCAGGTGATGCTGACAGAGG 3’, 5’ GGGATGAGCTAGTGCTGATCTGG 3’.

*m NQO1*, 5’ CAGCCAATCAGCGTTCGGTA 3’, 5’ CTTCATGGCGTAGTTGAATGATGTC 3’.

*m GCLC*, 5’ CAGTCAAGGACCGGCACAAG 3’, 5’ CAAGAACATCGCCTCCATTCAG 3’.

*m GPX2*, 5’ ACTTCACCCAGCTCAACGAG 3’, 5’ ATGCTCGTTCTGCCCATTCA 3’.

*m TFAM*, 5’ GAGCGTGCTAAAAGCACTGG 3’, 5’ CCACAGGGCTGCAATTTTCC 3’.

*m CO1*, 5’ GACTTGCAACCCTACACGGA 3’, 5’ GATGGCGAAGTGGGCTTTTG 3’.

*m ND1*, 5’ TCCTATCCACGCTTCCGCTA 3’, 5’ GGTGGTATCCCCGCTGTAAA 3’.

*h B7-H3*, 5’ GGACATAGCCCCTCGCCAC 3’, 5’ CTCGGTGTCCCCAAAGCATT 3’.

*h MMP-1*, 5’ AAAATTACACGCCAGATTTGCC 3’, 5’ GGTGTGACATTACTCCAGAGTTG 3’.

*h MMP-3*, 5’ CTGGACTCCGACACTCTGGA 3’, 5’ CAGGAAAGGTTCTGAAGTGACC 3’.

*h MMP-9*, 5’ TGTACCGCTATGGTTACACTCG 3’, 5’ GGCAGGGACAGTTGCTTCT 3’.

*h MMP-10*, 5’ TGCTCTGCCTATCCTCTGAGT 3’, 5’ TCACATCCTTTTCGAGGTTGTAG 3’.

*h p21*, 5’ TGTCCGTCAGAACCCATGC 3’, 5’ AAAGTCGAAGTTCCATCGCTC 3’.

*h p53*, 5’ CAGCACATGACGGAGGTTGT 3’, 5’ TCATCCAAATACTCCACACGC 3’.

*h TERT*, 5’ AAATGCGGCCCCTGTTTCT 3’, 5’ CAGTGCGTCTTGAGGAGCA 3’.

*h TFAM*, 5’ GGCAAGTTGTCCAAAGAAACC 3’, 5’ GCATCTGGGTTCTGAGCTTTA 3’.

*h CO1*, 5’ CTAGCAGGTGTCTCCTCTATCT 3’, 5’ GGCGTTTGGTATTGGGTTATG 3’.

*h ND1*, 5’ GAAGTCACCCTAGCCATCATTC 3’, 5’ GCAGGAGTAATCAGAGGTGTTC 3’.

*h ND6*, 5’ GGGTGGTGGTTGTGGTAAAC 3’, 5’ CCCCGAGCAATCTCAATTAC 3’.

### Analysis of mtRNA and mtDNA copy

We used qPCR to detect the relative copy number of mtDNA and mtRNA^56,57^. In brief, total DNA was extracted from cells, and 2.5 ng of the DNA was used for qPCR analysis. The mitochondrial ND1 gene (*mtND1*) was used to determine mtDNA copy which was normalized to the nuclear gene, *SDHA*. Primer designs were published before and applied in the later study^19,58^.

### Determination of cell cycle, ROS, TUNEL, mitoSOX, GSH-Px, SOD, CAT and MDA content

Flow cytometry analysis: For all flow cytometry experiments, cells were acquired on a Beckman Coulter CytoFLEX flow cytometer. Debris and dead cells were excluded based on forward scatter (FSC) and side scatter (SSC) gating. A minimum of 10,000 viable single cells were analyzed per sample. For each experiment, unstained controls and single-stained compensation controls were included to set appropriate gates and correct for spectral overlap.

Cell cycle analysis: Cells were collected, washed with ice-cold PBS, fixed with 70% cold ethanol, and stored at 4 °C for 24 h. After centrifugation, cells were washed twice with cold PBS and stained with PBS containing RNase A and propidium iodide (PI) in the dark for 30 min (Beyotime, China, C1052). PI fluorescence was detected in the FL3 channel, and cell cycle distribution (G1, S, G2 phases) was analyzed using FlowJo software. Debris and doublets were excluded by gating on FSC-A vs. FSC-H.

TUNEL assay: Apoptosis was detected using an Elabscience kit (E-CK-A420) following the manufacturer’s instructions; FITC-labeled apoptotic cells were detected by flow cytometry. The FITC-positive gate was set based on unstained control cells, and mean fluorescence intensity (MFI) was quantified for each sample.

Intracellular ROS measurement: ROS levels were measured using the DCFH-DA probe (Beyotime, China, S0033S). Cells were incubated with 1 μM DCFH-DA at 37 °C for 20 min, washed, and immediately analyzed by flow cytometry. The DCF fluorescence (FITC channel) was measured, and the geometric MFI was calculated. Unstained cells were used to set the background fluorescence threshold.

Mitochondrial ROS measurement: Mitochondrial superoxide was assessed using the mitoSOX probe (Beyotime, China, S0061S). Cells were incubated with 5 μM mitoSOX at 37 °C for 10 min, washed, and immediately analyzed by flow cytometry. MitoSOX fluorescence was detected in the PE channel, and the geometric mean fluorescence intensity was calculated. Unstained cells served as negative controls to establish baseline fluorescence.

SOD, GSH-Px, CAT activity, and MDA levels were measured with assay kits (Nanjing Jiancheng Bioengineering Institute, China, A001-3-2/A005-1-2/A007-1-1/A003-1-2) according to the manufacturers’ instructions. SOD activity was assayed using the WST-1 method. GSH-Px activity was estimated based on its catalyzation of the reduction reaction to hydrogen peroxide. CAT activity was measured by analyzing the rate at which it caused the decomposition of H_2_O_2_ at 405 nm. MDA content was evaluated according to the thiobarbituric acid (TBA) method, based on the spectrophotometric measurement of the color produced during the reaction to TBA with MDA, which was calculated at 532 nm.

### Mitochondrial morphological analysis

To assess mitochondrial ultrastructure, HaCaT cells were fixed and processed using standard protocols for dehydration and resin embedding. Ultrathin sections were prepared, stained with uranyl acetate and lead citrate, and imaged under a transmission electron microscope. Mitochondrial morphometric parameters, including the length-to-width ratio and area, were quantified from TEM images using ImageJ software. For each experimental group, mitochondria with intact cristae structures were selected from multiple cells. The length-to-width ratio was calculated as the major axis divided by the minor axis, and the area was determined using standardized measurement tools.

### Mitochondrial functional analysis

OCR was measured using the Extracellular OCR Plate Assay Kit (Dojindo, Kumamoto, Japan, E297) with phosphorescence-based detection. Mitochondrial mass was assessed using Mitotracker Red CMXRos (Beyotime, China, C1035). Cells were incubated with 100 nM Mitotracker at 37 °C for 30 min, washed, and analyzed by flow cytometry. Mitochondrial membrane potential was measured using the JC-1 assay kit (Beyotime, China, C2006). All protocols followed manufacturer guidelines with cell count normalization.

### Co-IP of intracellular proteins

Cells were cultured in 10 cm dishes until reaching 80–90% confluency. The cells were lysed using NP-40 lysis buffer (Beyotime, China, P0013F) on ice for 60 min. Lysates were centrifuged and the supernatant was collected. Total protein concentration in the supernatant was quantified using a BCA assay (Beyotime, China, P0010). Protein was transferred to a fresh 1.5 mL tube, and a target-specific antibody was added. The mixture was incubated at 4 °C for 12 h. For CO-IP, BeyoMag Anti-Flag Magnetic Beads (Beyotime, China, P2115) were added directly to the protein-antibody mixture and incubated for 2 h. The beads were washed ten times with ice-cold buffer. Finally, all samples were boiled for 15 min.

### Mass spectrometry

After B7-H3 CO-IP, immunoprecipitated complexes were washed extensively with ice-cold lysis buffer to remove nonspecific binding. Beads were resuspended and centrifuged to collect eluted proteins. Proteins were separated by SDS-PAGE and visualized by Coomassie blue staining. Gel lanes were excised into slices, subjected to in-gel trypsin digestion, and peptides were extracted with 50% acetonitrile/5% formic acid. Samples were analyzed by IP/MS using the Cosmos Wisdom Laboratory (Cosmos Wisdom, Hangzhou). Candidate interacting proteins were filtered by ≥2 unique peptides, <1% false discovery rate (FDR), and exclusion of contaminants/keratins.

### Machine learning

Based on our MS-based interactome profiling in HaCaT cells that identified B7-H3-interacting proteins, we focused on membrane-associated regulators linked to skin aging. To refine these candidates, we integrated the MS-derived membrane protein list with the skin aging transcriptomic dataset GSE7519*2* (young vs. aged skin samples, downloaded from GEO and normalized using R)^23^. Only genes present in both datasets were retained for subsequent analysis. Three machine-learning algorithms were then applied to screen for the most relevant membrane-associated proteins, all implemented in R with a fixed random seed to ensure reproducibility.

SVM-RFE^59^: Using the caret and e1071 packages, a linear-kernel SVM was trained with 10-fold cross-validation. Features were recursively eliminated (10% per step) based on their contribution to the model. The optimal feature subset was determined by the lowest cross-validation error rate.

RF^60^: The randomForest package was used to build a model with 500 decision trees, with sample age as the response variable. Feature importance was measured by the mean decrease in Gini index. Genes with an importance score above the average of all features were selected.

LASSO^61^: L1-regularized logistic regression was performed using the glmnet package. Ten-fold cross-validation was applied to select the optimal regularization parameter λ (lambda.min), and features with non-zero coefficients under this λ were retained.

The final core gene set was defined as the intersection of the features selected by all three algorithms (visualized by a Venn diagram). This integrated analysis identified *ITGA2* as the key membrane-associated interacting partner of B7-H3 in skin photodamage.

### Molecular docking

The amino acid sequences of human B7-H3 (UniProt Q5ZPR3-2) and human ITGA2 (UniProt E9PB77) were retrieved from UniProt. Their three-dimensional structures were predicted using AlphaFold 3.0 server. The predicted models with the highest confidence scores (iptm = 0.52, ptm = 0.60) were downloaded in PDB format. Rigid docking was performed using ZDOCK 3.0.2 with a rotational step size of 6°, generating 2000 initial poses. The top 100 poses by docking score were clustered based on root-mean-square deviation, and the representative pose from the largest cluster was selected as the most probable binding mode. The final complex structure was visualized and analyzed in PyMOL^62^.

### Origin of all cited figures

The elements of Fig. 8a and Supplementary Fig. 2a were created by the authors using Microsoft PowerPoint and Adobe Illustrator. No pre-existing templates, clip art, or previously published elements were used. No generative AI tools were employed in their creation. The graphical abstract was designed using FigDraw Scientific Illustration Platform (www.figdraw.com) and exported as a PNG file at 300 dpi resolution (ID:TIPIOa9194).

### Statistics and reproducibility

The results are presented as the mean ± standard deviation (SD). A minimum of 3 biological replicates was chosen, as well as a minimum of 2 independent experiments, to give statistical significance at data collection. All statistical tests were two-sided. Statistical differences were assessed using Student’s t-tests for two-group comparisons, one-way or two-way ANOVA for multi-group comparisons, followed by Tukey’s HSD or Bonferroni tests as appropriate. The statistical analysis was performed using SPSS 19.0 or GraphPad Prism 9.0 software. Statistical differences of *P* < 0.05 were considered significant.

### Ethical approval

This study was approved by the clinical research ethics committee of the First Affiliated Hospital of Soochow University (approval number: 2019251). Written informed consent was obtained from all participants. All ethical regulations relevant to human research participants were followed. All animal experiments in this work were approved by the ethics committee of Soochow University (approval number: SUDA20250521A01). We have complied with all relevant ethical regulations for animal use.

### Reporting summary

Further information on research design is available in the Nature Portfolio Reporting Summary linked to this article.

## Supplementary information

Supplementary Information

Description of Additional Supplementary Files

Supplementary Data 1, 2

Reporting summary

Transparent Peer Review file

## Data Availability

The mass spectrometry proteomics data have been deposited to the ProteomeXchange Consortium (https://proteomecentral.proteomexchange.org) via the iProX partner repository with the dataset identifier PXD078471^63,64^. The numerical source data for the graphs and charts presented in the main figures are provided in the Supplementary Data 2. The full uncropped and unedited blot images and the flow cytometry gating strategy are available in the Supplementary Information (Supplementary Figs. 8 and 9). All other data are available from the corresponding author upon reasonable request.

## Code availability

The machine learning analyses were performed using publicly available R packages with the parameters described in the Methods. The R scripts used for data processing and model training are available from the corresponding author upon reasonable request.
