# Author Correction: B7-H3 promotes skin photodamage through ITGA2-NRF2-TFAM-mediated mitochondrial oxidative stress

**DOI:** 10.1038/s42003-026-11134-7

**Published:** 2026-10-21

**Authors:** Yuanyuan Jia, Qiange Zhang, Fangying Su, Yuan Zhu, Siji Chen, Jingyi Yang, Qiuyu Mao, Yiwen Zhang, Yuting Yang, Sisi Ding, Cuiping Liu, Xueguang Zhang, Dan Luo, Wei Min, Lei Cao

**Affiliations:** 1 Jiangsu Institute of Clinical Immunology, The First Affiliated Hospital of Soochow University, Suzhou, Jiangsu Province China; 2 Department of Dermatology, JiangSu Province Hospital, The First Affiliated Hospital With Nanjing Medical University, Nanjing, Jiangsu Province China; 3 Jiangsu Key Laboratory of Clinical Immunology, Soochow University, Suzhou, Jiangsu Province China; 4 Jiangsu Key Laboratory of Gastrointestinal Tumor Immunology, The First Affiliated Hospital of Soochow University, Suzhou, Jiangsu Province China; 5 Department of Dermatology, People’s Hospital of Guangxi Zhuang Autonomous Region, Nanning, Guangxi Zhuang Autonomous Region China; 6 Department of Dermatology, First People’s Hospital of Changshu City, Changshu Hospital Affiliated of Soochow University, Changshu, Jiangsu Province China; 7 NanTong Tong Zhou Hospital of Chinese Traditional Medicine, Nantong, Jiangsu China; 8 Department of Dermatology, Kunshan First People’s Hospital, Suzhou, Jiangsu Province China; 9 Department of Dermatology, Minhang Hospital, Fudan University, Shanghai, China

**Keywords:** Skin diseases, Cell biology

Correction to: *Communications Biology* https://doi.org/10.1038/s42003-026-10387-6, published online 28 May 2026

In the version of the article initially published, the “WT, COL3A1” image in Fig. 3i was a duplicate of the “KO, COL1A1” image due to an error in figure assembly. Fig. 3i has now been updated to include the correct “WT, COL3A1” image in the HTML and PDF versions of the article

