# Supplementary material for: B7-H3 promotes skin photodamage through ITGA2-NRF2-TFAM-mediated mitochondrial oxidative stress
Source: Commun Biol. 2026 May 28;9:1134. doi: 10.1038/s42003-026-10387-6 (PMC13500712; doi:10.1038/s42003-026-10387-6)
Supplement: Supplementary file 1 [file 42003_2026_10387_MOESM1_ESM.pdf]

Supplementary Information

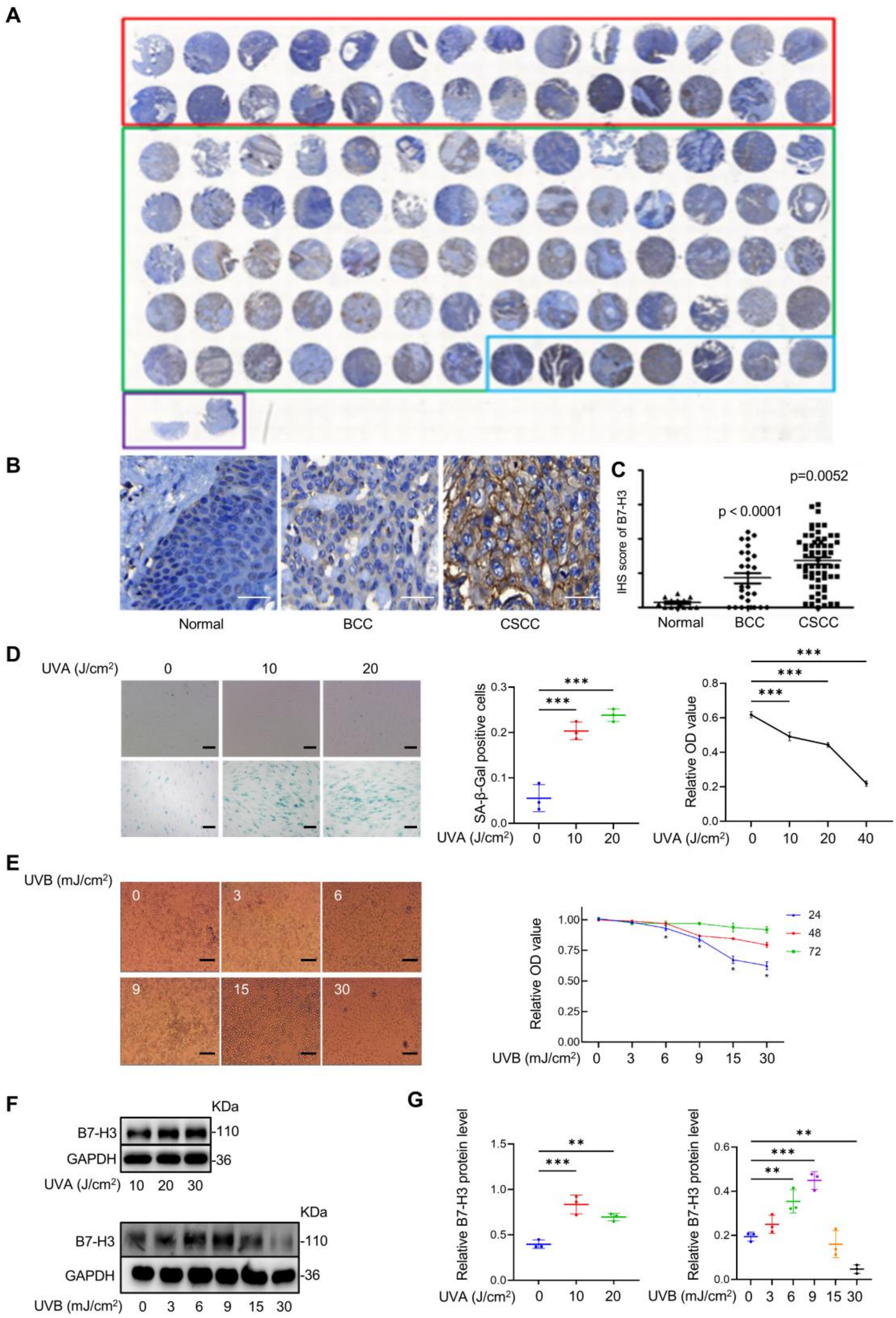

Supplementary Figure 1. B7-H3 is upregulated in both chronic photodamaged human skin and acute UVR-exposed cells. Irradiation conditions: For *in vitro* experiments, HSF cells were irradiated once with UVA at indicated doses (0, 10, 20, 40 J/cm^2) and harvested 24 h post-irradiation (D, F). HaCaT cells were irradiated once with UVB at indicated doses (0, 3, 6, 9, 15, 30 mJ/cm^2) and harvested at indicated

time points (0, 24, 48 h) for viability assessment (E), or at 24 h post-irradiation for protein expression analysis (F). (A) IHC staining of human BCC (red border), CSCC (green border), melanoma (blue border), and normal skin tissues (purple border). Scale bars: 100 μm as indicated in the figure. (B) B7-H3 was highly expressed in CSCC and BCC tissues and low in normal tissues. (C) IHC score showed that B7-H3 expression was significantly higher in CSCC and BCC. (D) Cell morphology and β-galactosidase activity of UVA-irradiated HSF cells. Scale bars: 40 μm as indicated in the figure. (E) Cell morphology and cell viability of UVB-irradiated HaCaT cells at indicated time points. Scale bars: 100 μm as indicated in the figure. (F) Western blot analysis of B7-H3 in UVA-irradiated HSF cells and UVB-irradiated HaCaT cells, with corresponding quantification graphs. n = 3 independent experiments. * represents $P < 0.05$; ** represents $P < 0.01$; and *** represents $P < 0.001$. Error bars represent mean ± SD.

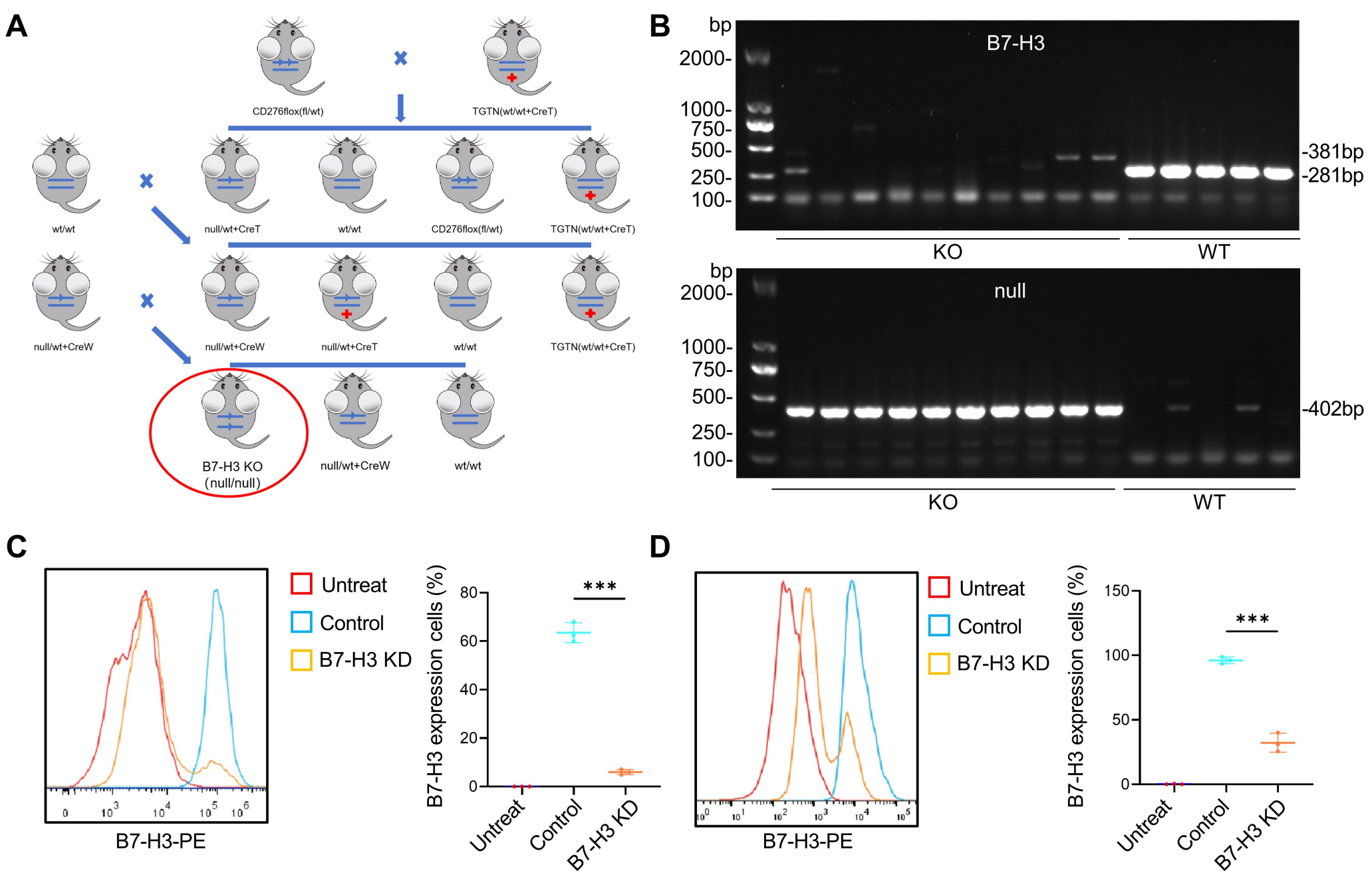

Supplementary Figure 2. Validation of B7-H3 deficiency models. (A) Schematic diagram of B7-H3 KO mice establishment. (B) Genotyping of KO mice, which exhibited negative bands for B7-H3 and positive bands for null. (C, D) FACS analysis of B7-H3 in B7-H3 knockdown HSF (C) and HaCaT (D) cells. n = 3 independent experiments. * represents $P < 0.05$; ** represents $P < 0.01$; and *** represents $P < 0.001$. Error bars represent mean ± SD.

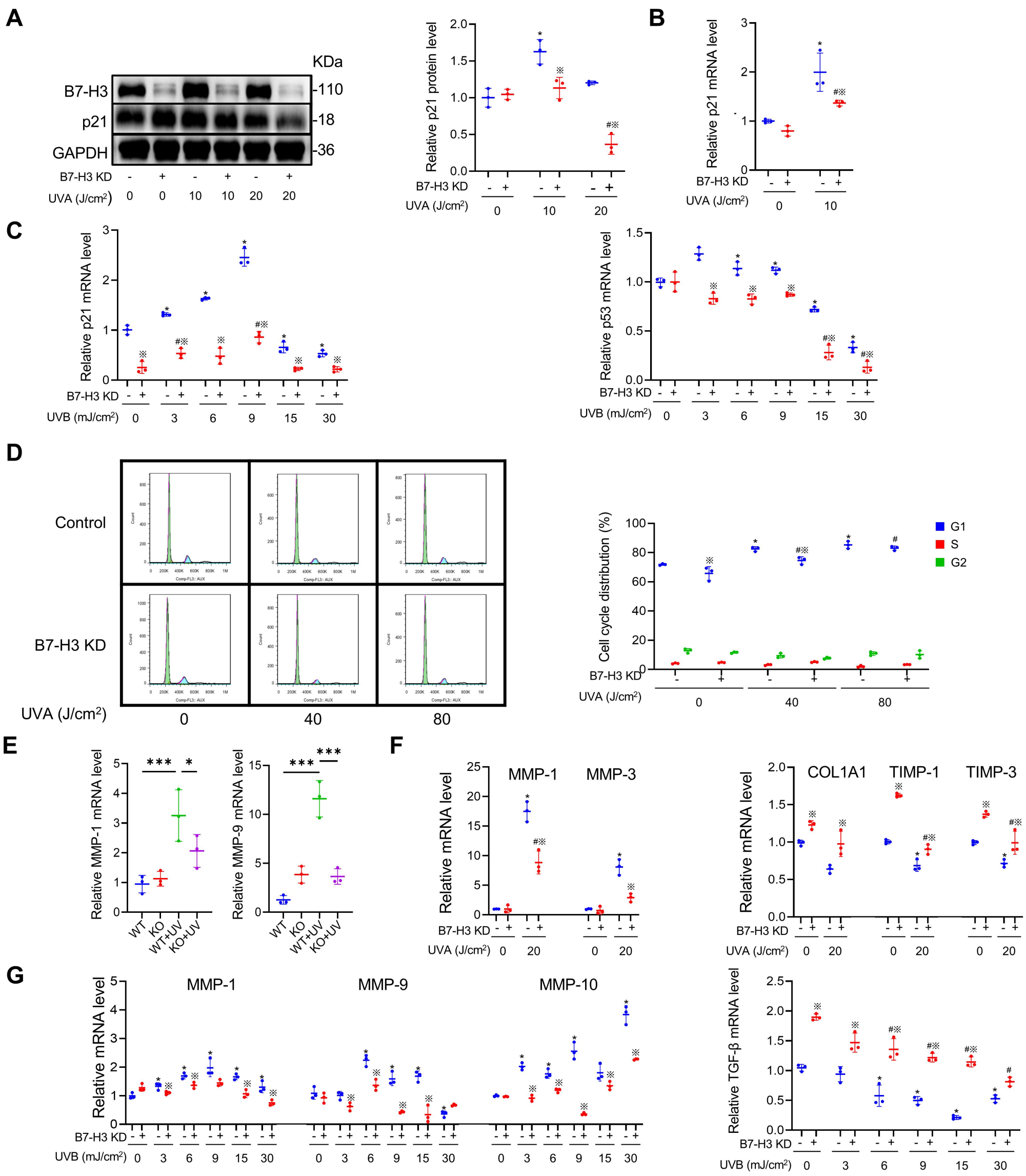

Supplementary Figure 3. B7-H3 mediates skin photodamage. Irradiation conditions: For *in vivo* experiments, mice were exposed to UVB irradiation (300 mJ/cm^2) daily for three consecutive days and skin samples were collected 24 h after the final exposure (E). For *in vitro* experiments, HSF cells were irradiated once with UVA at indicated doses (0, 10 or 20 J/cm^2 in A, B, F; 0, 40, or 80 J/cm^2 in D) and harvested 24 h post-irradiation. HaCaT cells were irradiated once with UVB at indicated doses (0, 3, 6, 9, 15, and 30 mJ/cm^2 in C, G) and harvested 24 h post-irradiation. (A, B) Protein and mRNA levels of p21 in UVA-irradiated HSF cells. (C) Protein and mRNA levels of *p21* and *p53* in UVB-irradiated HaCaT cells. (D) Cell cycle distribution analysis in UVA-irradiated HSF cells. (E) mRNA levels of collagen synthesis and degradation-related genes in UVB-irradiated mouse skin

tissues. (F) mRNA levels of collagen synthesis and degradation-related genes in UVA-irradiated HSF cells. (G) mRNA levels of collagen synthesis and degradation-related genes in UVB-irradiated HaCaT cells. n = 3 female mice or independent experiments. E: * represents $P < 0.05$; ** represents $P < 0.01$; *** represents $P < 0.001$. A-D, F, G: * $P < 0.05$ (Control + UVR vs Control); # $P < 0.05$ (B7-H3 KD + UVR vs B7-H3 KD); ※ $P < 0.05$ (B7-H3 KD + UVR vs Control + UVR, at the same dose). Error bars represent mean ± SD.

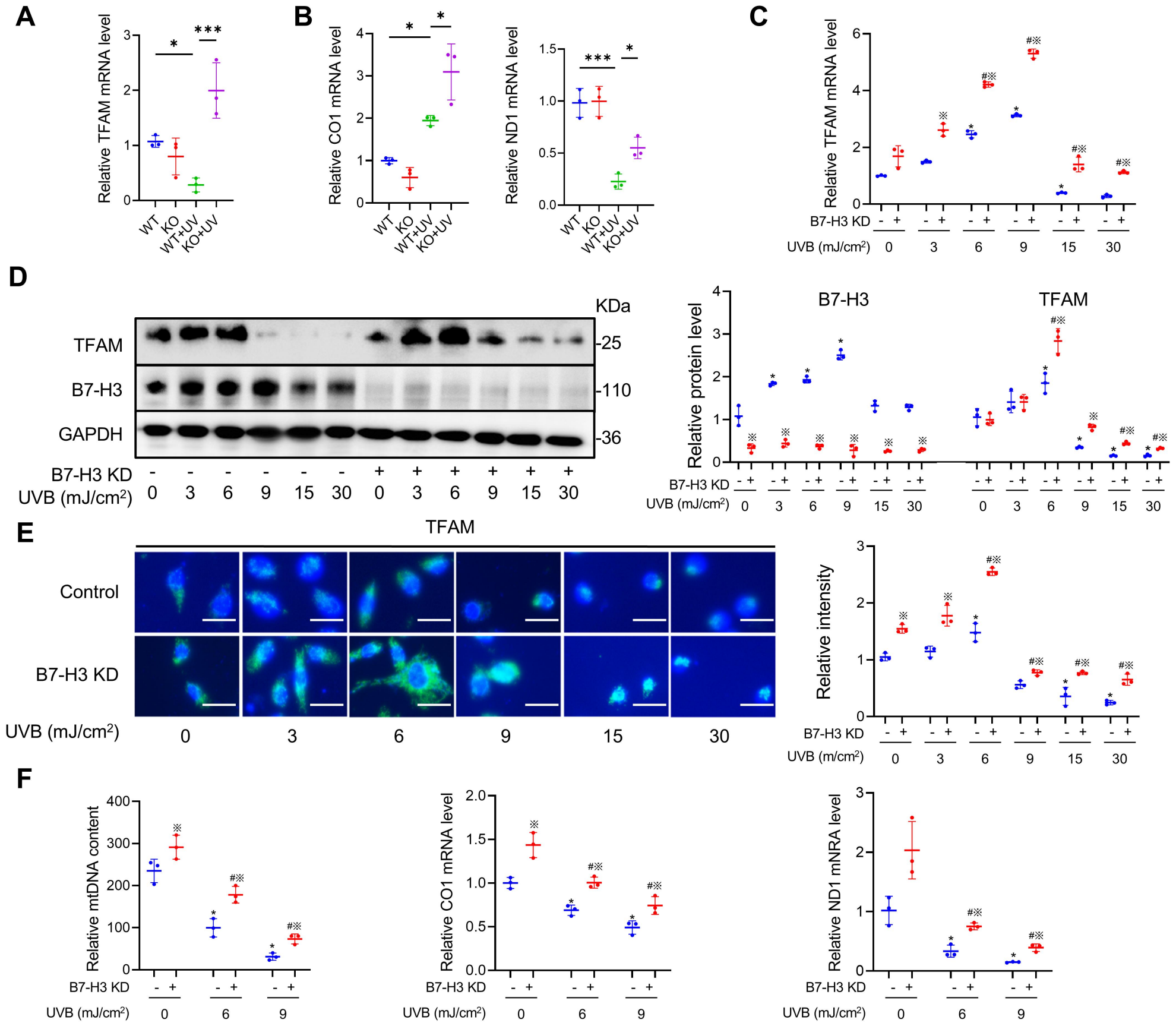

Supplementary Figure 4. TFAM inhibition and mitochondrial dysfunction in skin photodamage. Irradiation conditions: For *in vivo* experiments, mice were exposed to UVB irradiation (300 mJ/cm^2) daily for three consecutive days and skin samples were collected 24 h after the final exposure (A, B). For *in vitro* experiments, HaCaT cells were irradiated once with UVB at indicated doses (0, 3, 6, 9, 15, and 30 mJ/cm^2 in C-E; 0, 6, and 9 mJ/cm^2 in F) and harvested 24 h post-irradiation. (A, B) mRNA levels of *TFAM* (A) and mtDNA-encoded complex subunits (B) in skin tissues of mice. (C-E) TFAM analysis in HaCaT cells by qRT-PCR (C), western blot (D), and immunofluorescence staining (E). Scale bars: 20 µm as indicated in the figure. (F) Relative mtDNA content and mRNA levels of mtDNA-encoded complex subunits in HaCaT cells. n = 3 female mice or independent experiments. A, B: * represents $P < 0.05$; *** represents $P < 0.001$. C-F: * $P < 0.05$ (Control + UVR vs Control); # $P < 0.05$ (B7-H3 KD + UVR vs B7-H3 KD); ※ $P < 0.05$ (B7-H3 KD + UVR vs Control + UVR, at the same dose). Error bars represent mean ± SD.

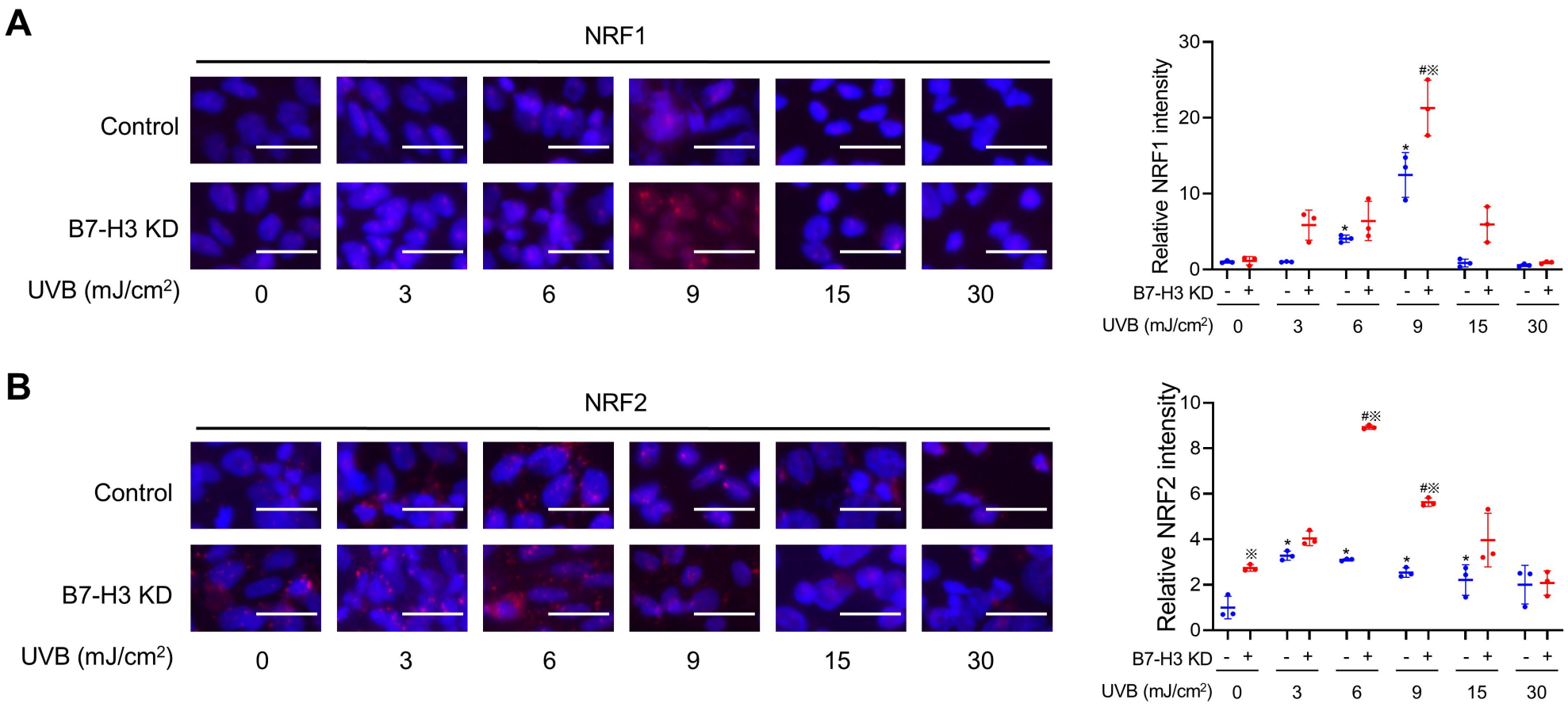

Supplementary Figure 5. NRF1 and NRF2 levels in photodamaged HaCaT cells. Irradiation conditions: HaCaT cells were irradiated once with UVB at indicated doses (0, 3, 6, 9, 15, and 30 mJ/cm^2) and harvested 24 h post-irradiation. (A, B) Immunofluorescence detection of NRF1 (A) and NRF2 (B) expression levels in UVB-irradiated HaCaT cells. Scale bars: 50 μm as indicated in the figure. n = 3 independent experiments.. * $P < 0.05$ (Control + UVR vs Control); # $P < 0.05$ (B7-H3 KD + UVR vs B7-H3 KD); ※ $P < 0.05$ (B7-H3 KD + UVR vs Control + UVR, at the same dose). Error bars represent mean ± SD.

A

B7-H3-interacting integrins
ITGA2
ITGA3
ITGAV
ITGA6
ITGB1
ITGB4

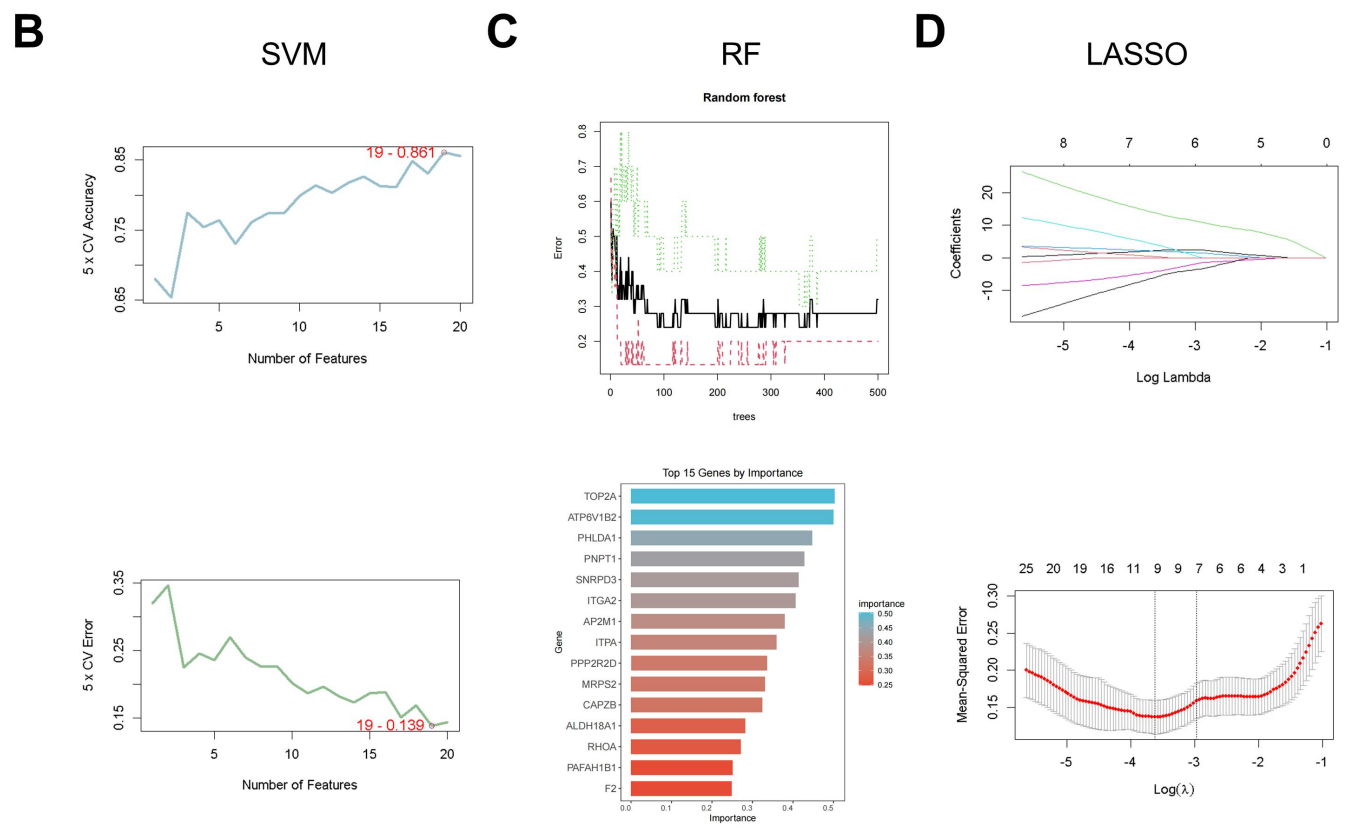

E

Feature Importance Comparison of ITGA2 and ITGB1

	ITGA2	ITGB1
SVM rank	4	16
RF importance	0.82991944165986	0.118410262655787
LASSO coef	6.60849144354408	-0.207557603

Supplementary Figure 6. Multi-omics prioritization identifies ITGA2 as the aging-associated molecule interacting with B7-H3. (A) IP/MS revealed potential interactions between B7-H3 and multiple integrin family members. (B-D) Schematic workflows of machine learning feature selection. (E) Integrative analysis of machine learning-derived feature rankings identified ITGA2 as the top aging-related integrin, with ITGB1 ranking lower in priority.

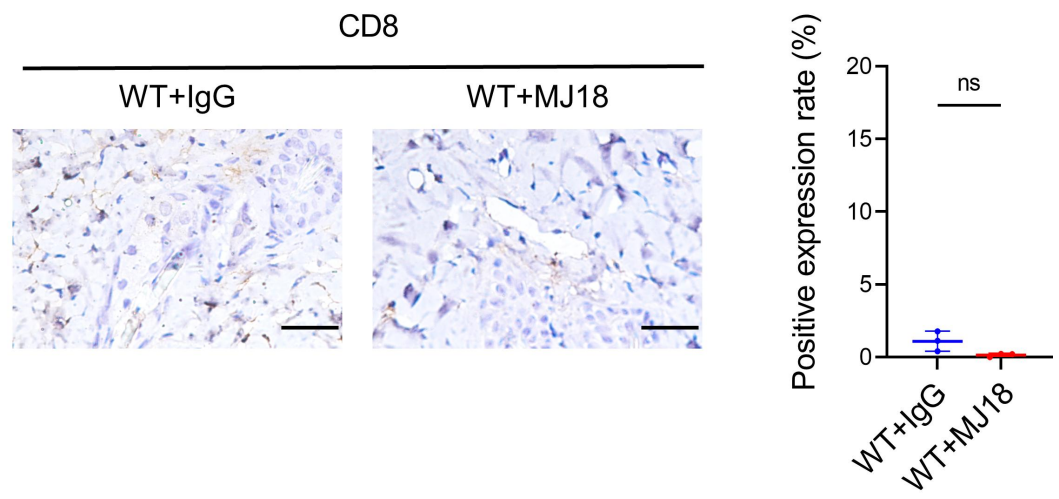

Supplementary Figure 7. Effects of B7-H3 blockade in CD8$^+$ cells. IHC analysis of CD8 of photodamaged skin tissues. Scale bars: 50 μm as indicated in the figure. *n* = 3 female mice. Error bars represent mean ± SD.

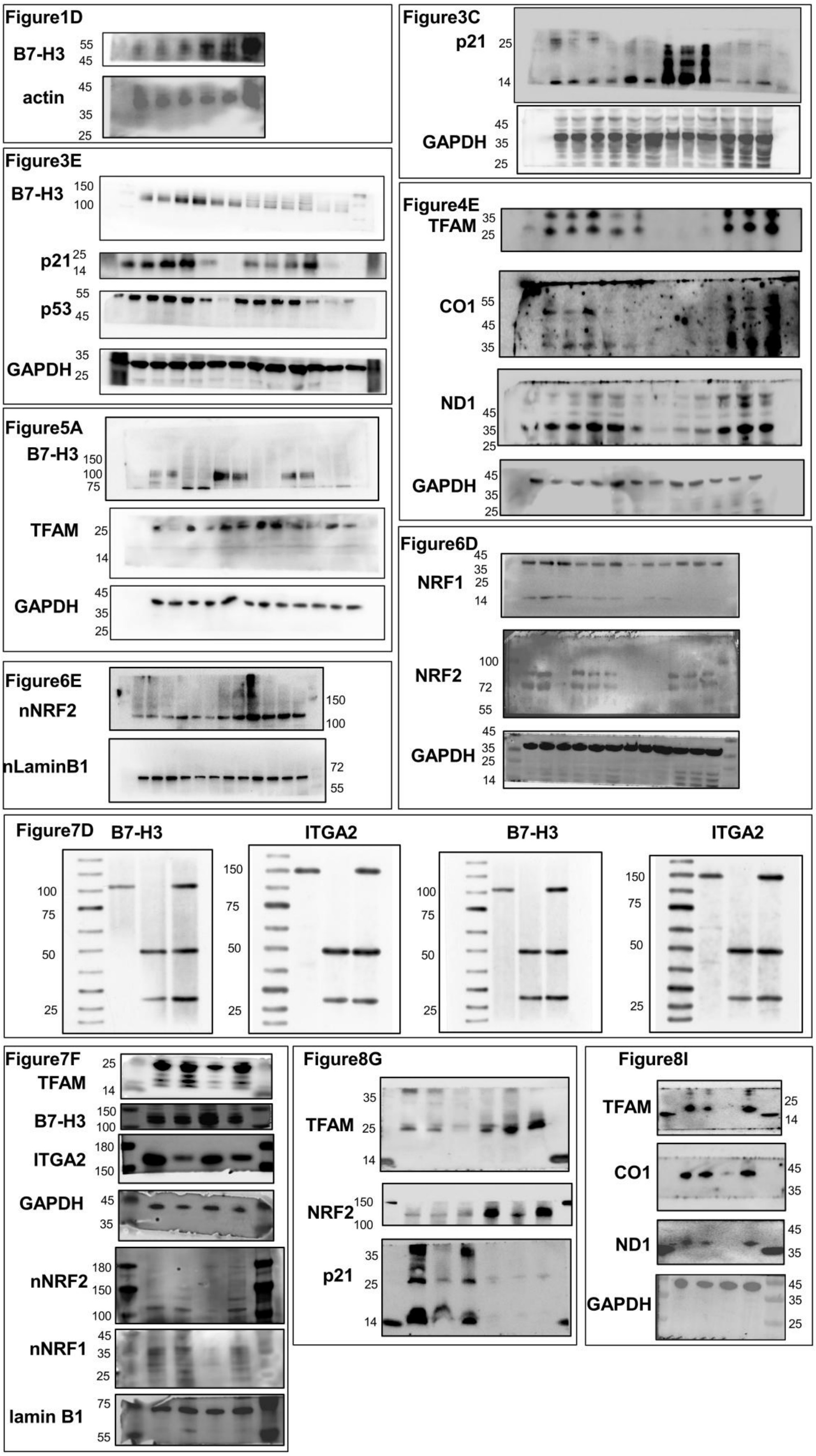

Supplementary Figure 8. Full uncropped and unedited blot images.

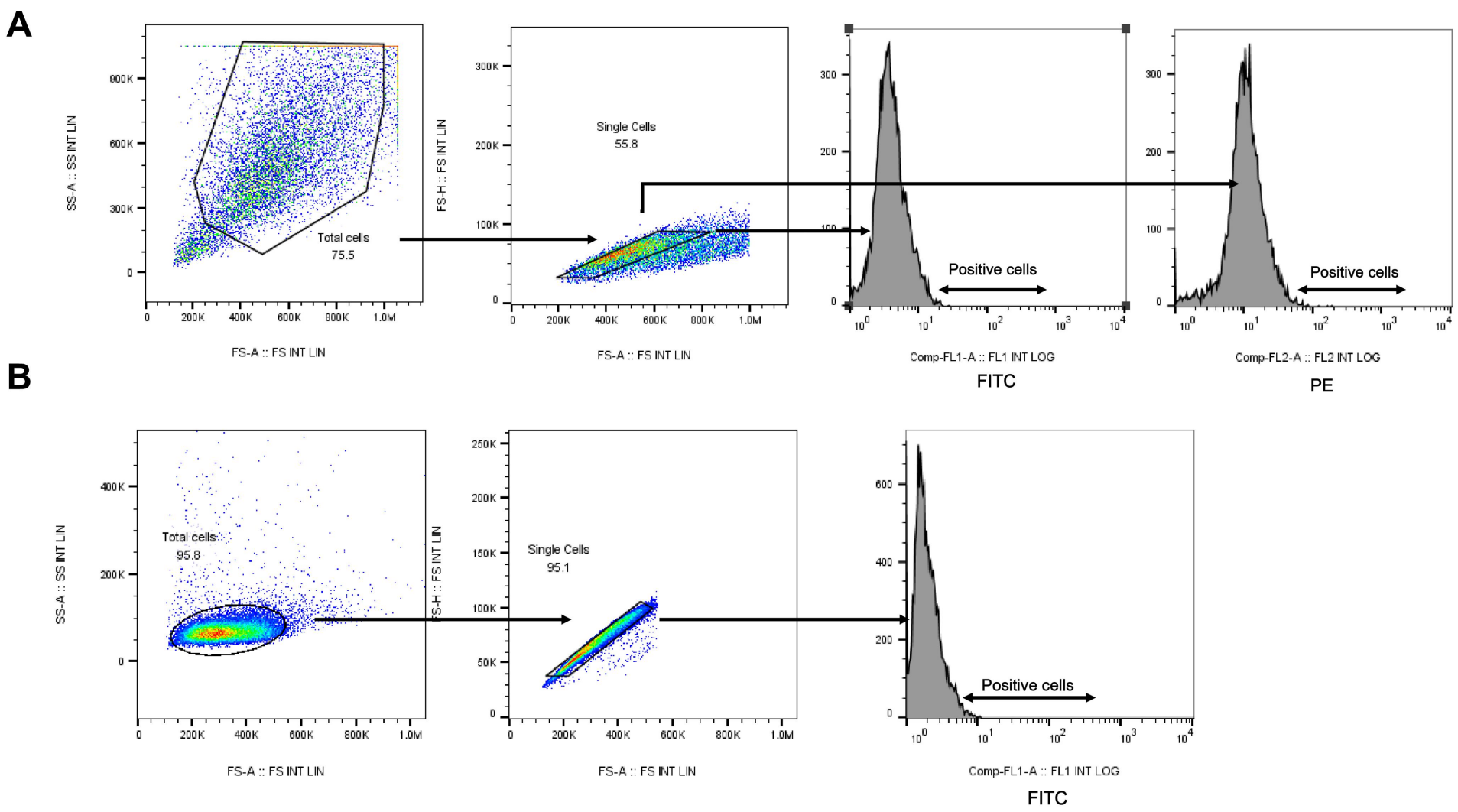

Supplementary Figure 9. The flow cytometry gating strategy. (A) Gating strategy for cultured cells. (B) Gating strategy for single-cell suspensions from mouse skin tissue.
