# Supplementary material for: B7-H3 promotes skin photodamage through ITGA2-NRF2-TFAM-mediated mitochondrial oxidative stress
Source: Commun Biol. 2026 May 28;9:1134. doi: 10.1038/s42003-026-10387-6 (PMC13500712; doi:10.1038/s42003-026-10387-6)
Supplement: Supplementary file 2 [file 42003_2026_10387_MOESM4_ESM.pdf]

nature portfolio

Corresponding author(s): Lei Cao

Last updated by author(s): May 4, 2026

Reporting Summary

Nature Portfolio wishes to improve the reproducibility of the work that we publish. This form provides structure for consistency and transparency in reporting. For further information on Nature Portfolio policies, see our Editorial Policies and the Editorial Policy Checklist.

Statistics

For all statistical analyses, confirm that the following items are present in the figure legend, table legend, main text, or Methods section.

n/a	Confirmed	
☐	☒	The exact sample size (*n*) for each experimental group/condition, given as a discrete number and unit of measurement
☐	☒	A statement on whether measurements were taken from distinct samples or whether the same sample was measured repeatedly
☐	☒	The statistical test(s) used AND whether they are one- or two-sided<br>*Only common tests should be described solely by name; describe more complex techniques in the Methods section.*
☒	☐	A description of all covariates tested
☐	☒	A description of any assumptions or corrections, such as tests of normality and adjustment for multiple comparisons
☐	☒	A full description of the statistical parameters including central tendency (e.g. means) or other basic estimates (e.g. regression coefficient) AND variation (e.g. standard deviation) or associated estimates of uncertainty (e.g. confidence intervals)
☒	☐	For null hypothesis testing, the test statistic (e.g. *F*, *t*, *r*) with confidence intervals, effect sizes, degrees of freedom and *P* value noted<br>*Give P values as exact values whenever suitable.*
☒	☐	For Bayesian analysis, information on the choice of priors and Markov chain Monte Carlo settings
☒	☐	For hierarchical and complex designs, identification of the appropriate level for tests and full reporting of outcomes
☒	☐	Estimates of effect sizes (e.g. Cohen's *d*, Pearson's *r*), indicating how they were calculated

Our web collection on statistics for biologists contains articles on many of the points above.

Software and code

Policy information about availability of computer code

Data collection	DMetrix imaging system (DMetrix, USA), Leica, Chemi DocTM MP Imaging System (Bio-Rad, USA), CFX96 TouchTM Real-Time PCR system (Bio-Rad, CA, USA), Olympus
Data analysis	Graphpad Prism, Image J, SPSS, Adobe Illustrator, AlphaFold, PyMOL

For manuscripts utilizing custom algorithms or software that are central to the research but not yet described in published literature, software must be made available to editors and reviewers. We strongly encourage code deposition in a community repository (e.g. GitHub). See the Nature Portfolio guidelines for submitting code & software for further information.

Data

Policy information about availability of data

All manuscripts must include a data availability statement. This statement should provide the following information, where applicable:

- Accession codes, unique identifiers, or web links for publicly available datasets
- A description of any restrictions on data availability
- For clinical datasets or third party data, please ensure that the statement adheres to our policy

The mass spectrometry data generated in this study have been deposited in Figshare under https://doi.org/10.6084/m9.figshare.3214684663. The numerical source data for the graphs and charts presented in the main figures are provided in the Supplementary Data 2. The full uncropped and unedited blot images and the flow

cytometry gating strategy are available in the Supplementary Information (Supplementary Figures 8 and 9). All other data are available from the corresponding author upon reasonable request.

Research involving human participants, their data, or biological material

Policy information about studies with human participants or human data. See also policy information about sex, gender (identity/presentation), and sexual orientation and race, ethnicity and racism.

Reporting on sex and gender	In our study, 50 patients were enrolled, including 27 males and 23 females. Statistical comparison showed no significant differences in sex between groups.
Reporting on race, ethnicity, or other socially relevant groupings	N/A
Population characteristics	50 patients were enrolled, including 27 males and 23 females, aging from 27 to 71 years old, with an average age of 42.5 years old. All skin samples were surgically resected from patients who went to Department of Dermatology, the First Affiliated Hospital of Soochow University, Soochow, China.
Recruitment	Participants were recruited from the Department of Dermatology, the First Affiliated Hospital of Soochow University, Soochow, China. The clinical data of each patient included sex, age, occupation, surgical site and photodamaged severity, which was evaluated by two qualified dermatologists.
Ethics oversight	This study was approved by the clinical research ethics committee of the First Affiliated Hospital of Soochow University (approval number: 2019251).

Note that full information on the approval of the study protocol must also be provided in the manuscript.

Field-specific reporting

Please select the one below that is the best fit for your research. If you are not sure, read the appropriate sections before making your selection.

☒ Life sciences ☐ Behavioural & social sciences ☐ Ecological, evolutionary & environmental sciences

For a reference copy of the document with all sections, see nature.com/documents/nr-reporting-summary-flat.pdf

Life sciences study design

All studies must disclose on these points even when the disclosure is negative.

Sample size	Sample sizes in animal experiments, qRT-PCR, WB, immunofluorescence recording were chosen based on published literatures in this field. For immunostaining, experiments were conducted on tissues from at least three different mice, with multiple slices per mouse being sampled and then averaged.
Data exclusions	No data or samples were excluded from the analysis. All collected samples met the inclusion criteria and were analyzed.
Replication	All findings were independently confirmed in multiple biological replicates. Consistent results were observed across patient samples and technical replicates.
Randomization	Mice were randomly assigned to model induction and treatment for in vivo experiments.
Blinding	Blinding was not applicable, as group identity was apparent during sample handling and analysis.

Reporting for specific materials, systems and methods

We require information from authors about some types of materials, experimental systems and methods used in many studies. Here, indicate whether each material, system or method listed is relevant to your study. If you are not sure if a list item applies to your research, read the appropriate section before selecting a response.

Materials & experimental systems

n/a	Involved in the study
☐	☒ Antibodies
☐	☒ Eukaryotic cell lines
☒	☐ Palaeontology and archaeology
☐	☒ Animals and other organisms
☒	☐ Clinical data
☒	☐ Dual use research of concern
☒	☐ Plants

Methods

n/a	Involved in the study
☒	☐ ChIP-seq
☐	☒ Flow cytometry
☒	☐ MRI-based neuroimaging

Antibodies

Antibodies used

Anti-human 4IgB7-H3 antibody (R&D Systems, USA, AF1027, 1:200 for IHC; Proteintech, China, 14453-1-AP, 1:5000 for western blot); anti-mouse B7-H3 antibody (Santa Cruz, USA, sc-376769, 1:5000 for western blot, 1:200 for IHC); anti-human p21 antibody (Cell Signaling Technology, USA, 2947S, 1:1000 for western blot); anti-mouse p21 antibody (Proteintech, China, 28248-1-AP, 1:1000 for western blot, 1:200 for IHC); anti-human p53 antibody (Cell Signaling Technology, USA, 9282S, 1:1000 for western blot); anti-mouse COL1A1 antibody (Proteintech, China, 66761-1-Ig, 1:100 for IHC); anti-mouse COL3A1 antibody (Proteintech, China, 22734-1-AP, 1:100 for IHC); anti-human TFAM antibody (Proteintech, China, 22586-1-AP, 1:5000 for western blot, 1:100 for IHC, 1:200 for immunofluorescence); anti-mouse TFAM antibody (Santa Cruz, USA, sc-166965, 1:1000 for western blot, 1:100 for IHC); anti-human/mouse NRF1 antibody (Santa Cruz, USA, sc-28379, 1:1000 for western blot, 1:50 for IHC, 1:50 for immunofluorescence); anti-human NRF2 antibody (Santa Cruz, USA, sc-365949, 1:1000 for western blot, 1:50 for IHC, 1:100 for immunofluorescence); anti-mouse NRF2 (Proteintech, China, 16396-1-AP); anti-human/mouse CO1 antibody (Invitrogen, USA, 1D6E1A8, 1:1000 for western blot, 1:50 for IHC); anti-human/mouse ND1 antibody (Proteintech, China, 19703-1-AP, 1:1000 for western blot, 1:100 for IHC); anti-human/mouse ITGA2 antibody (Abcam, USA, ab181548, 1:10000 for western blot, 1:500 for IHC), anti-human TOMM20 antibody (Abcam, USA, ab186735, 1:250 for immunofluorescence), anti-human DNA antibody (AC-30-10, Germany, Progen, 1:200 for immunofluorescence).

Validation

All the primary antibodies are validated by the manufacturer and used in published literatures. The manufacturer names and catalogue numbers are provided in the methods of manuscript.

Eukaryotic cell lines

Policy information about cell lines and Sex and Gender in Research

Cell line source(s)

HSF and HaCaT (National Collection of Authenticated Cell Cultures, Chinese Academy of Sciences) were maintained in our laboratory and used in our study.

Authentication

N/A

Mycoplasma contamination

Cells were not tested for mycoplasma contamination.

Commonly misidentified lines (See ICLAC register)

N/A

Animals and other research organisms

Policy information about studies involving animals; ARRIVE guidelines recommended for reporting animal research, and Sex and Gender in Research

Laboratory animals

Six-eight-weeks-old C57BL/6J mice were obtained from the Jiangsu Institute of Clinical Immunology.

Wild animals

N/A

Reporting on sex

Female mice were used for the main experiment.

Field-collected samples

N/A

Ethics oversight

All animal experiments in this work were approved by the ethics committee of Soochow University (approval number: SUDA20250521A01), and carried out following the currently prescribed guidelines.

Note that full information on the approval of the study protocol must also be provided in the manuscript.

Plants

Seed stocks	N/A
Novel plant genotypes	N/A
Authentication	N/A

Flow Cytometry

Plots

Confirm that:

☒ The axis labels state the marker and fluorochrome used (e.g. CD4-FITC).

☒ The axis scales are clearly visible. Include numbers along axes only for bottom left plot of group (a 'group' is an analysis of identical markers).

☒ All plots are contour plots with outliers or pseudocolor plots.

☒ A numerical value for number of cells or percentage (with statistics) is provided.

Methodology

Sample preparation	Harvested and digested cells were washed twice with PBS. The cell pellets were resuspended and incubated with fluorescent reagents or fluorescent-labeled antibodies at room temperature. After incubation, the cells were washed again, then immediately subjected to flow cytometric analysis with all procedures protected from light.
Instrument	Flow cytometer
Software	Flowjo
Cell population abundance	Approximately 10,000 cells were analyzed per sample
Gating strategy	A FSC-A/SSC-A scatter plot was used for gating to exclude cell debris and aggregates, and the main cell population was delineated. FSC-A/FSC-W parameters were applied to further screen and eliminate doublet interference, ensuring the analyzed objects were single cells. Based on the fluorescence channels corresponding to different detection indicators, positive thresholds were set with reference to isotype controls for quantitative analysis of target signals. (Supplementary Figure 9)

☒ Tick this box to confirm that a figure exemplifying the gating strategy is provided in the Supplementary Information.
