# Supplementary material for: B7-H3 promotes skin photodamage through ITGA2-NRF2-TFAM-mediated mitochondrial oxidative stress
Source: Commun Biol. 2026 May 28;9:1134. doi: 10.1038/s42003-026-10387-6 (PMC13500712; doi:10.1038/s42003-026-10387-6)
Supplement: Supplementary file 3 [file 42003_2026_10387_MOESM5_ESM.pdf]

nature portfolio

Peer Review File

B7-H3 Promotes Skin Photodamage Through ITGA2-NRF2-TFAM-Mediated Mitochondrial Oxidative Stress

Corresponding Author: Dr Lei Cao

This file contains all reviewer reports in order by version, followed by all author rebuttals in order by version.

Version 0:

Reviewer comments:

Reviewer #1

(Remarks to the Author)
This manuscript reports that B7-H3 is induced by UVR in human skin and experimental models and proposes that B7-H3 promotes photodamage by engaging ITGA2, suppressing NRF2 nuclear translocation and the NRF1–TFAM axis, impairing mitochondrial homeostasis, and increasing ROS. The authors further suggest therapeutic benefit using an anti–B7-H3 blocking antibody in a UVB mouse model. The topic is timely and potentially impactful, as it connects an immune checkpoint–associated molecule with mitochondrial redox regulation in cutaneous photodamage. However, several issues related to experimental design and interpretation should be addressed to strengthen causal inferences and translational relevance.

1. Methods
• Please provide clear criteria for classifying the human clinical samples as normal, naturally aged, and sun-exposed (photodamaged and photoaged) skin. In addition, please explicitly define what constitutes acute versus chronic UVR exposure in the mouse models (e.g., UV source, dose per exposure, and sampling time points).
• Anti–B7-H3 antibody (MJ18) delivery and controls: Because MJ18 (or IgG) was administered using a 200 μm micro-needle roller in the UVB model, please include an appropriate needle-roller-only (no antibody) and/or sham roller control to account for potential confounding effects from microneedle-induced injury, inflammation, and wound-healing responses. If additional controls cannot be added, please explicitly discuss this limitation and its implications for interpretation.
• Please describe in detail how each scoring system was calculated in the human ex vivo and mouse models.
2) “Reduction of B7-H3 mitigates UVR-induced ROS accumulation”
- The authors state that “skin lesion severity was markedly reduced in KO mice compared to WT controls” (Fig. 2B, C). Please clarify what constitutes “lesion severity”.
- Please revise the wording describing antioxidant enzyme changes. Instead of stating “imbalanced levels of SOD/CAT/GSH-Px,” please specify the exact findings.
3) “Decline of B7-H3 alleviates UVR-induced photoaging”
• The experiments described (UVA/UVB doses with short-term responses) largely reflect acute stress responses, rather than long-term photoaging or “chronic accumulation” of photoaging markers (line 145). Please adjust the terminology accordingly.
• For the TERT finding, please state clearly whether the change was measured at mRNA levels.
• ECM conclusions appear primarily based on mRNA. This is insufficient to support strong claims about ECM degradation/collagen suppression without protein/activity validation. Please add supporting evidence or temper the conclusion.
4) “B7-H3 negatively regulates TFAM and leads to mitochondrial dysfunction”
• There appears to be a wording inconsistency: you wrote “mechanisms by which B7-H3 deletion promotes ROS accumulation,” whereas the results indicate B7-H3 deletion reduces ROS. Please correct.
• The explanation that “low-dose UVR did not decrease TFAM due to compensatory repair” is speculative without DNA repair markers.
5) “NRF1 and NRF2 levels are negatively correlated with B7-H3 expression”
• NRF2 nuclear localization is useful, but nuclear NRF2 does not necessarily equate to transcriptional output. Please include at least 1–2 canonical NRF2 target genes (e.g., HO-1, NQO1, GCLC) to support functional suppression. Please explicitly discuss this limitation.
• Clarify NRF1 measurement. Avoid using “translocation” unless this is directly demonstrated.
7) Therapeutic efficacy of anti–B7-H3 mAb (MJ18)

- Please provide mechanistic validation demonstrating that MJ18 effectively engages and/or blocks B7-H3 in your system.
8) Discussion
• The Discussion contains statements implying direct causality (e.g., “cooperative inhibition” via B7-H3–ITGA2; “B7-H3 as a transcriptional repressor of TFAM”) where the evidence more strongly supports association and functional dependence (co-IP, rescue experiments, and downstream phenotypes) rather than direct transcriptional control. Please tighten the language to distinguish what is shown from what is inferred, presenting the latter as a working model with specific future study.
• Please be cautious with claims about chronic photoaging/photocarcinogenesis if the in vivo work is based on acute UV injury.
• The MJ18 section would benefit from more cautious framing given unresolved target specificity and possible procedural influences of local delivery; positioning the antibody data as supporting feasibility (rather than definitive therapeutic validation) would strengthen credibility.
9) Figure legends
• Define UV conditions and timepoints in each legend (UVA vs UVB, dose, exposure schedule, harvest time).
• Use consistent terminology (UVR with explicit UVA/UVB specification, and consistent use of “photodamage/photoaging/acute photodamage” with definitions).
• Provide necessary assay details where needed: ROS probe (DCFH-DA/DHE/MitoSOX), flow cytometry details (basic gating description), and how “photodamage score” and “epidermal thickening score” were calculated (or clearly point to Methods).
10) Clarity, terminology, and typos
• Replace “malignant endpoints of chronic photodamage” with “UV-associated non-melanoma skin cancers commonly linked to chronic sun exposure.”
• Replace “direct B7-H3-interacting partner” with “B7-H3-associated interactor supported by IP/MS and co-IP.”
• Correct the typo: “B7-H3 deletion promotes ROS accumulation” → “reduces ROS accumulation.”
• Ensure consistent NRF1/NRF2 capitalization throughout.

Reviewer #3

(Remarks to the Author)
In this manuscript, the authors investigate the role of the immune checkpoint protien B7-H3 in skin cell responses to UV radiation. The provide strong, quantitaive and thorough data that disruption of B7-H3 alters antioxidant signaling and protects mitochondria against photodamage. They also provide some translational potential of their work through the use of a blocking monoclonal antibody that they inject into mouse skin following UV exposure. Given the importance of UV exposure and its impact on photodamage and photoaging, this work should have a wide interest to the field.

I have only a few minor concerns regarding a lack of information about some of the methodology:
1) In Figure 1B, how did the authors differentiate normal and naturally aged skin? If this was based on subject age, it would be useful to provide information somewhere about the age ranges of the subjects in these two groups.

2) The authors mention using a UVB phototherapy device and a solar simulator in the methods section but do not provide any information about the model/manufacturer. This information is important should other scientists wish to replicate or extend the findings.

3) In various places in the text (such as lines 132 and 138), it is not particularly clear from how the text is written which group/treatment is more affected by the treatment. For example, on lines 131-132, the authors state "Consistent with this, both in vivo and in vitro skin photodamage models, elevated ROS levels were observed" but do not state which group (WT or KO) has higher ROS levels. Obviously the reader can look at the figure to determine this, but the text would read better if the results were more clearly stated.

Version 1:

Reviewer comments:

Reviewer #1

(Remarks to the Author)
The authors have satisfactorily addressed the reviewers’ comments and questions. The manuscript has been substantially improved, and the revised version can be accepted for publication.

Reviewer #3

(Remarks to the Author)
The authors have adequately addressed my concerns.

Open Access This Peer Review File is licensed under a Creative Commons Attribution 4.0 International License, which permits use, sharing, adaptation, distribution and reproduction in any medium or format, as long as you give appropriate credit to the original author(s) and the source, provide a link to the Creative Commons license, and indicate if changes were made.
In cases where reviewers are anonymous, credit should be given to 'Anonymous Referee' and the source.
The images or other third party material in this Peer Review File are included in the article's Creative Commons license, unless indicated otherwise in a credit line to the material. If material is not included in the article's Creative Commons license and your intended use is not permitted by statutory regulation or exceeds the permitted use, you will need to obtain permission directly from the copyright holder.
To view a copy of this license, visit https://creativecommons.org/licenses/by/4.0/

Dear reviewers,

We are deeply grateful for your time, expertise, and the exceptionally thoughtful comments provided on our manuscript. Your insights have been truly invaluable, guiding us to substantially improve the clarity, rigor, and overall quality of our work. We have carefully addressed every point raised and have revised the manuscript accordingly, with all changes made in direct response to your constructive feedback.

Below we provide our point-by-point responses. To facilitate review, we have uploaded two versions of the manuscript: a clean version and a version with all changes highlighted in red. We remain grateful for the opportunity to improve our work through your valuable input.

Reviewer #1 (Remarks to the Author):

1. Methods

- **Please provide clear criteria for classifying the human clinical samples as normal, naturally aged, and sun-exposed (photodamaged and photoaged) skin. In addition, please explicitly define what constitutes acute versus chronic UVR exposure in the mouse models (e.g., UV source, dose per exposure, and sampling time points).**

We thank the reviewer for this valuable suggestion. We have now provided clear criteria for the classification of human skin samples and explicitly defined the UVR exposure protocols in the mouse models in the Methods section.

(1)Classification of Human Skin Samples:

As suggested, we have now clearly defined the four groups of human skin samples (normal, naturally aged, photodamaged and photoaged) based on clinical diagnosis and histopathological evaluation. The detailed classification criteria, including donor age ranges and clinical characteristics for each group, have been added to the revised Methods section. (Page 18, Line 2)

"Based on clinical diagnosis and histopathological evaluation by two qualified dermatologists, the samples were classified into four groups: normal skin (n = 15, obtained from sun-protected areas such as post-auricular and upper inner arm regions of young to middle-aged donors with no history of significant sun exposure and no clinical signs of photoaging, mean age 32.9 ± 5.6 years); naturally aged skin (n = 8, obtained from sun-protected areas of donors presenting with typical senile skin changes including fine wrinkling and laxity attributable to chronological aging, but lacking the characteristic features of photoaging, mean age 57.3 ± 7.9 years); photodamaged skin (n =12, obtained from sun-exposed areas such as face and forearm of donors who, despite regular sunlight exposure, did not exhibit severe clinical signs of photoaging such as deep wrinkles or leathery appearance, mean age 33.8 ± 6.4 years); and photoaged skin (n = 15, obtained from severely sun-exposed areas such as face of donors presenting with classic clinical features of photoaging, including deep wrinkles, leathery appearance, and pigmentary irregularities, mean age 51.3 ± 10.1 years)."

(2)Definition of UVR Exposure in Mouse Models:

We appreciate the opportunity to clarify our experimental design. In this study,

we established two distinct models: an acute UVB irradiation model to investigate initial photodamage responses, and a separate antibody treatment model with a modified timeline to evaluate therapeutic efficacy. The UV source, irradiation parameters, exposure schedules, and sampling time points for both models are now explicitly defined in the Methods section, with the antibody treatment timeline also illustrated in Figure 8A. (Page19, Line 10)

"After shaving the dorsal skin of mice with a blade, hair removal cream was applied again to ensure complete depilation. To model acute UVB-induced skin damage, mice were irradiated using a UVB phototherapy device (SH2 A, Sigma, Shanghai, China). The UVB phototherapy device was placed in a dark room, and the mice were exposed to a single dose of 0 or 300 mJ/cm^2 of UVB for three consecutive days, a well-established protocol for inducing acute photodamage[54,55]. For antibody treatment experiments, mice were randomly divided into two groups: UVB irradiation + control IgG (UVB + IgG) and UVB irradiation + anti-B7-H3 antibody (UVB + MJ18). Following the same irradiation protocol (300 mJ/cm^2 on days 1, 2, and 3), mice received subcutaneous injections of 100 μg of anti-mouse CD276/B7-H3 monoclonal antibody (clone MJ18, catalog number BE0124, BioXcell, USA) or control rat IgG (catalog number BE0290, BioXcell, USA) on days 2, 4, and 6 using a 200 μm micro-needle roller. The experimental timeline is illustrated in Figure 8A. The injections were carefully performed to ensure consistent delivery of the antibody or control. Twenty-four hours after the final treatment, morphological changes in the dorsal skin of the mice were observed."

We believe these additions and clarifications have adequately addressed the reviewer's concern.

- **Anti–B7-H3 antibody (MJ18) delivery and controls: Because MJ18 (or IgG) was administered using a 200 μm micro-needle roller in the UVB model, please include an appropriate needle-roller-only (no antibody) and/or sham roller control to account for potential confounding effects from microneedle-induced injury, inflammation, and wound-healing responses. If additional controls cannot be added, please explicitly discuss this limitation and its implications for interpretation.**

We appreciate the reviewer's concern regarding the lack of a micro-needle-only control group in our antibody treatment experiment. We have addressed this limitation in the revised Discussion and would like to provide additional context to support our interpretation.

First, microneedling is a well-established technique in dermatology for enhancing transdermal drug delivery. As documented in the literature, microneedles penetrate the stratum corneum to form temporary micro-channels, enabling efficient delivery of therapeutic agents to the dermis. This technology has been widely applied in treating various skin conditions, including photoaging. Importantly, clinical studies have demonstrated that while microneedles facilitate drug penetration, the therapeutic effects are primarily attributable to the delivered agents rather than the microneedling procedure itself. For example, a split-face clinical trial showed that microneedle

patches containing active ingredients (acetyl hexapeptide-8 or EGF) achieved significantly greater wrinkle improvement compared to microneedle patches alone.

Second, although we did not include a separate micro-needle-only control in this specific experiment, our preliminary studies (unpublished data) using the same UVB photodamage model compared mice treated with UVB + micro-needle + saline against UVB-only controls. These pilot experiments showed no significant difference in photodamage severity between the two groups, suggesting that micro-needle treatment alone does not appreciably alleviate UVB-induced skin damage in our model.

Third, we included an IgG control group that underwent identical micro-needle administration, allowing us to control for non-specific effects of the injection procedure. The significant difference between MJ18-treated and IgG-treated groups supports that the observed effects are B7-H3-specific rather than procedural artifacts.
Nevertheless, we acknowledge that micro-needle delivery may introduce confounding factors such as needle-induced inflammation or wound healing responses. We have explicitly acknowledged this limitation in the revised Discussion and have added a sentence to contextualize microneedling as a standard transdermal delivery approach (Page 16, Line 29):

"In addition, the use of a micro-needle roller for local antibody delivery may introduce confounding factors such as needle-induced inflammation or wound healing responses, which could influence the observed therapeutic effects independently of B7-H3 blockade. Although microneedling is a well-established technique in dermatology for enhancing transdermal drug delivery and clinical studies indicate that its primary role is to facilitate drug penetration rather than to confer direct therapeutic benefit, we cannot completely exclude the possibility that the micro-needling procedure itself contributed to the observed effects."

- **Please describe in detail how each scoring system was calculated in the human ex vivo and mouse models.**

We thank the reviewer for requesting clarification on the scoring systems. We have now provided detailed descriptions of how each scoring system was calculated in both the human ex vivo and mouse models in the revised Methods section.
(1)Human skin sample classification:

As described in our response to the previous comment, the classification of human skin samples into normal, naturally aged, photodamaged, and photoaged groups was based on clinical diagnosis and histopathological evaluation by two qualified dermatologists. The detailed criteria for each group, including donor age, sampling sites, and clinical characteristics, have been added to the Methods section.
(2)Mouse skin photodamage scoring :

The photodamage scoring for macroscopic assessment of mouse dorsal skin has been described in the Methods section. To ensure objectivity, the scoring was performed by two independent investigators, and the average score was calculated for each mouse. This clarification has now been added to the revised Methods section. (Page 19, Line 27)

“Photodamage scoring: Images of the dorsal skin of the mice were captured using a camera, and skin lesion severity was assessed according to established photodamage scoring criteria, which ranged from 0 to 2[54]. The scoring was performed by two independent investigators, and the average score was calculated for each mouse. 0: flesh-colored, smooth, and plump skin; 0.5: flesh-colored, slightly rough skin; 1: red patches with moderately rough skin and deepened skin lines; 1.5: red patches with noticeably rough skin, minor flaking, and some wrinkles; 2: a purple-red ulcerative condition with significantly rough and thickened skin, extensive flaking, and increased wrinkles.”

2.“Reduction of B7-H3 mitigates UVR-induced ROS accumulation”

- **The authors state that “skin lesion severity was markedly reduced in KO mice compared to WT controls” (Fig. 2B, C). Please clarify what constitutes “lesion severity”.**

We thank the reviewer for pointing out the lack of clarity in our description of skin lesion severity. We have revised the text to explicitly define what this encompasses. In the revised manuscript, we now specify that skin lesion severity refers to both macroscopic observations of typical acute photodamage (including erythema, scaling, and dryness) and histological assessment of epidermal thickening, both of which were significantly reduced in KO mice compared to WT controls following UVR exposure. The photodamage scoring and epidermal thickness measurements used for assessment has been detailed in the Methods section as described in our previous response. The revised sentence is as follows (Page 5, Line 18):

“Following UVR exposure, the severity of acute photodamage, including erythema, scaling, dryness, and epidermal thickening, was markedly reduced in KO mice compared to WT controls, as assessed by both macroscopic scoring and histological analysis (Fig. 2B, C). ”

- **Please revise the wording describing antioxidant enzyme changes. Instead of stating “imbalanced levels of SOD/CAT/GSH-Px,” please specify the exact findings.**

Regarding the description of antioxidant enzyme changes, we agree that the original wording “imbalanced levels” was vague and have revised it to clearly state the specific findings. The original sentence has been replaced with (Page 5, Line 28):

“Consistent with the elevated ROS levels, UVR induced significant alterations in antioxidant enzyme activities and oxidative damage markers. *In vivo*, UVB-exposed WT mouse skin exhibited a compensatory increase in superoxide dismutase (SOD) activity, alongside a significant reduction in glutathione peroxidase (GSH-Px) activity and elevated malondialdehyde (MDA) levels, indicating an imbalance in antioxidant defense and subsequent lipid peroxidation. Compared to UVB-exposed WT mice, B7-H3 KO mice displayed further enhanced SOD activity, restored GSH-Px activity, and markedly reduced MDA accumulation (Fig. 2G). In UVB-irradiated HaCaT and UVA-irradiated HSF cells, B7-H3 knockdown significantly attenuated the suppression

of SOD, catalase (CAT), and GSH-Px activities and reduced MDA accumulation compared to control cells (Fig. 2H, I). ”

3.“Decline of B7-H3 alleviates UVR-induced photoaging”

- **The experiments described (UVA/UVB doses with short-term responses) largely reflect acute stress responses, rather than long-term photoaging or “chronic accumulation” of photoaging markers (line 145). Please adjust the terminology accordingly.**

We are grateful to the reviewer for pointing out the vagueness in our description. Since our experiments were conducted using acute UVR exposure models, the observed changes reflect early molecular events associated with photoaging rather than chronic accumulation of photoaging phenotypes. We have revised the terminology throughout this section to accurately reflect this distinction. Specifically, we have:

1.Changed the section title from Decline of B7-H3 alleviates UVR-induced photoaging to Decline of B7-H3 attenuates UVR-induced early stress responses associated with photoaging.

2.Revised the introductory sentence to clarify that we are examining early stress responses that are associated with the development of photoaging rather than chronic phenotypes.

3.Reworded the concluding sentences to accurately reflect that our findings identify B7-H3 as a regulator of acute UVR-induced cellular changes that are associated with photoaging development.

The full revised paragraph is as follows (Page 6, Line 11):

“**Decline of B7-H3 attenuates UVR-induced early stress responses associated with photoaging**

In addition to amplifying UVR-induced ROS generation—a hallmark of acute photodamage, we further identified that B7-H3 critically drives early stress responses that are associated with the subsequent development of photoaging.……Together, these findings identify B7-H3 as a key regulator of multiple cellular changes induced by acute UVR. These changes are associated with the development of photoaging, suggesting that targeting B7-H3 may mitigate these early stress responses.”

- **For the TERT finding, please state clearly whether the change was measured at mRNA levels.**

We thank the reviewer for pointing out this omission. We have now revised the text to explicitly state that the TERT change was measured at the mRNA level. Revised sentence (Page 6, Line 15): “B7-H3 inhibition protected against UVA-induced reduction in telomerase reverse transcriptase (TERT) mRNA levels in fibroblasts (Fig. 3A).”

- **ECM conclusions appear primarily based on mRNA. This is insufficient to support strong claims about ECM degradation/collagen suppression without protein/activity validation. Please add supporting evidence or temper the**

conclusion.

We thank the reviewer for this insightful comment. We agree that conclusions regarding ECM remodeling and collagen regulation should be supported by evidence at the protein level. In the original manuscript, our findings on ECM-related gene expression were based solely on mRNA data (Supplementary Fig. 3E-G). To address this concern, we have now performed IHC staining to examine collagen protein expression *in vivo*.

As shown in Fig. 3I and 3J, UVB irradiation significantly reduced the protein levels of COL1A1 and COL3A1 in murine skin, whereas B7-H3 knockout substantially rescued their expression. These new data provide direct protein-level evidence that B7-H3 contributes to collagen suppression upon UVR exposure. Accordingly, we have revised the corresponding section in the Results to present both the mRNA and protein findings, and we have tempered the conclusion to more accurately reflect the strength of the evidence. The revised text now reads (Page 7, Line 1):

"Consistently, IHC analysis revealed that UVB-induced downregulation of collagen I (COL1A1) and collagen III (COL3A1) in murine skin was significantly rescued upon B7-H3 knockout (Fig. 3I, J), further supporting a role for B7-H3 in promoting ECM degradation and suppressing collagen synthesis at the protein level. "

4."B7-H3 negatively regulates TFAM and leads to mitochondrial dysfunction"

- **There appears to be a wording inconsistency: you wrote "mechanisms by which B7-H3 deletion promotes ROS accumulation," whereas the results indicate B7-H3 deletion reduces ROS. Please correct.**

We thank the reviewer for catching this critical wording error. We have corrected this wording inconsistency. The revised sentence now reads (Page 7, Line 14): "To elucidate the molecular mechanisms by which B7-H3 deletion attenuates ROS accumulation, we analyzed both mRNA and protein expression profiles of TFAM."

- **The explanation that "low-dose UVR did not decrease TFAM due to compensatory repair" is speculative without DNA repair markers.**

We thank the reviewer for this critical observation. The original speculation about "compensatory DNA repair responses" was inappropriate given that we did not directly assess DNA repair markers. We have removed this speculation entirely from the text. Additionally, upon re-examining our data, we have revised the description to accurately reflect the dose-dependent effects of UVB on TFAM expression. The corrected sentence now reads (Page 7, Line 21):

"In vitro, low-dose UVB (3 and 6 mJ/cm^2) did not significantly decrease TFAM levels in HaCaT cells. However, UVB irradiation at 9 mJ/cm^2 markedly suppressed both TFAM mRNA and protein expression (Supplementary Fig. 4C-F)."

5."NRF1 and NRF2 levels are negatively correlated with B7-H3 expression"

- **NRF2 nuclear localization is useful, but nuclear NRF2 does not necessarily equate to transcriptional output. Please include at least 1–2 canonical NRF2**

target genes (e.g., HO-1, NQO1, GCLC) to support functional suppression. Please explicitly discuss this limitation.

We thank the reviewer for this constructive comment. We agree that NRF2 nuclear translocation alone does not fully demonstrate its transcriptional activity. In response, we have examined the expression of canonical NRF2 target genes in UVB irradiated murine skin samples.

We performed qRT-PCR to assess *HO-1, NQO1, GCLC*, and *GPX2*. As shown in Fig. 6F, *HO-1* and *NQO1* were induced upon UVB exposure, and this induction was further enhanced in B7-H3 KO mice. In contrast, *GCLC* and *GPX2* showed decreased expression following UVB irradiation. For *GCLC*, this reduction was significantly rescued in B7-H3 KO mice; for *GPX2*, a similar trend toward restoration was observed, though it did not reach statistical significance. Despite the distinct regulatory patterns among these target genes, the consistent finding that B7-H3 depletion either increased or restored their expression collectively indicates that B7-H3 suppresses NRF2 dependent antioxidant gene expression. Together with the observed inhibition of NRF2 nuclear translocation, these data provide functional evidence that B7-H3 impairs NRF2 transcriptional activity under UVR induced oxidative stress.

We have incorporated these new data into the manuscript accordingly. In the Results section, we have supplemented the original paragraph with the expression patterns of these four target genes. The Discussion section has been revised to integrate this functional validation, supporting the conclusion that B7-H3 suppresses NRF2-related transcriptional activity. The Methods section has been updated with the qRT-PCR details, and the Figure 6 legend has been expanded to include panels F.

The revised text is as follows:

Results (Page 9, Line 26):

"To further assess whether the reduced NRF2 nuclear translocation translates into impaired transcriptional activity, we examined the mRNA levels of canonical NRF2 target genes in UVB-irradiated murine skin samples. We observed two distinct patterns of regulation (Fig. 6F). *HO-1* (heme oxygenase-1) and *NQO1* (NAD(P)H: quinone oxidoreductase 1), which are classic rapid-response NRF2 targets, were induced upon UVB exposure; this induction was further enhanced in B7-H3 KO mice. In contrast, *GCLC* (glutamate-cysteine ligase catalytic subunit) and *GPX2* (glutathione peroxidase 2), which encode key enzymes for glutathione synthesis and utilization, showed decreased expression following UVB irradiation. For *GCLC*, this reduction was significantly rescued in B7-H3 KO mice. For *GPX2*, a similar trend toward restoration was observed, though it did not reach statistical significance. Despite the distinct regulatory patterns among these target genes, a difference that likely reflects differential promoter sensitivity, feedback adaptation, or the balance between stress induction and metabolic exhaustion, the consistent finding that B7-H3 depletion either increased or restored their expression collectively indicates that B7-H3 suppresses NRF2-dependent antioxidant gene expression. Together with the observed inhibition of NRF2 nuclear translocation, these data provide functional evidence that B7-H3 impairs NRF2 transcriptional activity under UVR-induced

oxidative stress."
Discussion (Page 13, Line 29):

"In UVB-irradiated murine skin, B7-H3 knockout enhanced the induction of *HO-1* and *NQO1* and restored the UVB-suppressed expression of *GCLC*, whereas *GPX2* displayed a similar trend toward restoration that did not reach statistical significance. Despite these target genes exhibited distinct regulatory patterns, their consistent increase or restoration upon B7-H3 depletion supports that B7-H3 suppresses NRF2 transcriptional activity, thereby diminishing ROS scavenging capacity and promoting oxidative stress[35,36]."

We believe these additions and revisions fully address the reviewer's concern. We appreciate the valuable feedback, which has helped strengthen our mechanistic evidence.

6.Clarify NRF1 measurement. Avoid using "translocation" unless this is directly demonstrated.

We thank the reviewer for this helpful clarification. We acknowledge that the term "nuclear translocation" should not be used for NRF1, as our data only demonstrate increased nuclear protein levels rather than active translocation. We have revised the text accordingly (Page 10, Line 30): "Specifically, depletion of ITGA2 enhanced nuclear protein levels of both NRF1 and NRF2, as well as increased total TFAM protein expression (Fig. 7E, F)."

7.Therapeutic efficacy of anti–B7-H3 mAb (MJ18)

- **Please provide mechanistic validation demonstrating that MJ18 effectively engages and/or blocks B7-H3 in your system.**

We thank the reviewer for raising this important point regarding direct evidence for MJ18 engagement with B7-H3. We fully appreciate that such validation would strengthen the conclusions.

MJ18 is a commercially available function-blocking mAb against mouse B7-H3, and its specific binding and blocking activity have been well characterized in multiple independent studies. Nagashima et al. originally generated and validated MJ18 as a neutralizing antibody that specifically recognizes B7-H3 and inhibits its function in T cell responses (Nagashima et al., Int Immunol, 2008). Subsequently, this antibody has been widely used as a validated tool for functional B7-H3 blockade in various murine disease models, including asthma and cancer (Yamato et al., J Immunol, 2017; Cheng et al., JCI Insight, 2024). These established reports provide a strong literature-based foundation for interpreting MJ18 as a specific B7-H3-blocking agent in our experimental system.

Building on this, multiple lines of evidence from our own data collectively suggest that MJ18 acts through B7-H3-related pathways in skin photodamage. The effects of MJ18 treatment closely parallel those observed in B7-H3 KO mice and B7-H3 KD cells across multiple functional readouts, including restored TFAM expression, reduced ROS accumulation, and attenuated photodamage. The consistency of these phenotypes across three independent loss-of-function models

(genetic knockout, siRNA knockdown, and antibody blockade) provides functional evidence that targeting B7-H3 yields reproducible protective effects. Furthermore, MJ18 retained its efficacy in cultured primary fibroblasts, a system devoid of T cells, demonstrating that the antibody can act directly on non-immune cells. This is particularly relevant given that MJ18 was originally characterized in immune contexts, and our data extend its known activity to direct effects on skin cells. T cell infiltration in the skin was minimal and did not differ significantly between treatment groups, which, while not conclusive, offers no direct support for an immune-mediated mechanism.

We acknowledge that direct biochemical evidence for MJ18 binding to B7-H3 is not provided in this study, and the precise molecular mechanism, particularly whether MJ18 blocks the B7-H3/ITGA2 interaction identified here, remains to be fully defined. We have incorporated these limitations into the revised Discussion while presenting the functional data as supportive evidence for the feasibility of B7-H3 targeting. We hope this combination of historical context and functional validation provides a reasonable basis for interpreting the antibody data within the scope of this study.

The relevant portion of the revised Discussion now reads (Page 15, Line 26):

"To assess the effects of B7-H3 blockade in skin photodamage, we utilized MJ18, a commercially available and well-characterized function-blocking mAb that specifically recognizes mouse B7-H3 and has been shown to disrupt its interaction with potential receptors in multiple previous studies[48,49]. Subsequently, this antibody has been employed as a validated tool for functional B7-H3 blockade in various disease models, including asthma and cancer[50]. In our study, MJ18 treatment restored TFAM and ETC-related protein expression in the skin and primary fibroblasts of UVR-exposed mice, reducing oxidative stress and mitigating tissue injury. These findings suggest that MJ18 interferes with endogenous B7-H3-driven signaling cascades. Given that MJ18 was originally characterized in studies where it modulated T cell responses, we considered whether its protective effects in our model might be mediated through T cells. However, several observations argue against this possibility. To explore this, we examined T cell infiltration in the skin. T cell numbers were low across all groups and did not differ significantly between treatment conditions (data not shown). While this observation does not exclude the possibility that resident or transient immune cells contribute to the response, it provides no direct support for an immune-mediated mechanism. More importantly, MJ18 retained its efficacy in cultured primary fibroblasts, a system devoid of T cells, demonstrating that the antibody can act directly on non-immune cells. These findings suggest that while MJ18 can modulate T cell function in immune contexts, its activity in our model reflects a direct effect on skin cells. This interpretation is further supported by the consistency between the effects of MJ18 treatment and those observed in B7-H3 KO mice and B7-H3 KD cells, including restored TFAM expression, reduced ROS accumulation, and attenuated photodamage. The concordance of these phenotypes provides functional evidence that MJ18 targets the same B7-H3-driven pathway identified throughout this study. Given the well-documented specificity of MJ18 as a validated B7-H3-blocking antibody in the literature, these convergent data from

multiple loss-of-function models collectively support the conclusion that the observed protective effects are attributable to B7-H3 blockade rather than off-target antibody effects.

Nevertheless, we acknowledge that direct biochemical evidence for MJ18 binding to B7-H3 is not provided here. The precise molecular mechanism remains to be fully defined, particularly whether MJ18 specifically blocks the B7-H3/ITGA2 interaction identified in this study or acts through other B7-H3-associated proteins such as IL20RA, MVP, or Fn, which have been shown to mediate diverse functions in tumor biology independent of adaptive immunity[51-53]."

We hope that this revised text provides a reasonable basis for interpreting the antibody data within the scope of this study. We have also ensured that the language appropriately reflects the strength of the evidence, positioning MJ18 treatment as supporting the feasibility of targeting B7-H3 rather than as definitive mechanistic validation.

8.Discussion

- **The Discussion contains statements implying direct causality (e.g., "cooperative inhibition" via B7-H3–ITGA2; "B7-H3 as a transcriptional repressor of TFAM") where the evidence more strongly supports association and functional dependence (co-IP, rescue experiments, and downstream phenotypes) rather than direct transcriptional control. Please tighten the language to distinguish what is shown from what is inferred, presenting the latter as a working model with specific future study.**

We thank the reviewer for this important observation regarding the language in the Discussion section. We have carefully revised the entire Discussion to clearly distinguish between experimentally supported findings and inferred mechanistic models. In particular, we have removed or rephrased statements implying direct causality, such as "transcriptional repressor of TFAM" and "cooperative inhibition via B7-H3–ITGA2 interaction", and instead framed inferred mechanisms as working models using phrases like "support a model in which" and "suggesting that". We have also added qualifying language (is associated with, contributes to) when describing relationships that lack direct molecular evidence, and explicitly noted that certain mechanistic details, particularly whether B7-H3 directly regulates TFAM transcription or acts indirectly, require further validation.

A representative example of these revisions is the change from "Our findings position B7-H3 as a transcriptional repressor of TFAM" to "Our findings suggest that B7-H3 negatively regulates TFAM expression, positioning it as a key upstream modulator whose suppression amplifies UVR-induced mitochondrial dysfunction".

Another representative example is the change from "This interaction potentially establishes a self-reinforcing vicious cycle of oxidative stress → mitochondrial damage → matrix remodeling → senescence amplification. This proposed regulatory axis expands the current understanding of integrins as active participants in photodamage beyond their conventional roles" to "These observations raise the possibility that this interaction could contribute to a self-reinforcing cycle involving

oxidative stress, mitochondrial damage, matrix remodeling, and senescence amplification, a concept that has been proposed in the context of photoaging[7,46,47]. However, given that our data are derived from acute UVR exposure models, further long-term studies are needed to directly establish such a cycle in chronic photodamage." (Page 15, Line 3).

All such changes have been incorporated throughout the Discussion section. We believe these revisions appropriately reflect the strength of our evidence while presenting the proposed mechanistic model as a framework for future investigation.

- **Please be cautious with claims about chronic photoaging/photocarcinogenesis if the in vivo work is based on acute UV injury.**

We are deeply grateful for this insightful guidance. The reviewer rightly points out that our original language in the Discussion overstated causal relationships. We have carefully revised the Discussion to ensure that any references to long-term processes are either supported by citations to prior literature or explicitly framed as speculative extensions of our acute findings. Specifically, we have modified the following sentence:

Original: This stress-dependent ROS amplification may serve as a key driver of long-term photodamage, potentially laying the foundation for malignant transformation.

Revised: This stress-dependent ROS amplification may contribute to the accumulation of cellular damage that, over repeated exposures, could promote long-term photodamage and increase skin cancer risk, as suggested by previous studies linking oxidative stress to photoaging and carcinogenesis. (Page 12, Line 28).

All other references to chronic processes in the Discussion are supported by citations to the literature rather than claims derived directly from our acute model data. We believe these revisions appropriately address the reviewer's concern.

- **The MJ18 section would benefit from more cautious framing given unresolved target specificity and possible procedural influences of local delivery; positioning the antibody data as supporting feasibility (rather than definitive therapeutic validation) would strengthen credibility.**

We appreciate the reviewer's guidance on this point. Following the suggestion, we have carefully revised the MJ18 discussion to adopt a more cautious tone. We have adjusted the opening wording, incorporated multiple lines of functional evidence to support that MJ18 acts through B7-H3 related pathways, explicitly acknowledged the unresolved questions regarding target specificity, and included a discussion of the potential confounding effects of micro-needle delivery. Throughout the revised paragraph, we have used more cautious language and positioned the data as supporting the feasibility of B7-H3 targeting rather than as definitive therapeutic validation. The revised paragraph now reads (Page 15, Line 26):

"To assess the effects of B7-H3 blockade in skin photodamage, we utilized MJ18, a commercially available and well-characterized function-blocking mAb that

specifically recognizes mouse B7-H3 and has been shown to disrupt its interaction with potential receptors in multiple previous studies[48,49]. Subsequently, this antibody has been employed as a validated tool for functional B7-H3 blockade in various disease models, including asthma and cancer[50]. In our study, MJ18 treatment restored TFAM and ETC-related protein expression in the skin and primary fibroblasts of UVR-exposed mice, reducing oxidative stress and mitigating tissue injury. These findings suggest that MJ18 interferes with endogenous B7-H3-driven signaling cascades. Given that MJ18 was originally characterized in studies where it modulated T cell responses, we considered whether its protective effects in our model might be mediated through T cells. However, several observations argue against this possibility. To explore this, we examined T cell infiltration in the skin. T cell numbers were low across all groups and did not differ significantly between treatment conditions (data not shown). While this observation does not exclude the possibility that resident or transient immune cells contribute to the response, it provides no direct support for an immune-mediated mechanism. More importantly, MJ18 retained its efficacy in cultured primary fibroblasts, a system devoid of T cells, demonstrating that the antibody can act directly on non-immune cells. These findings suggest that while MJ18 can modulate T cell function in immune contexts, its activity in our model reflects a direct effect on skin cells. This interpretation is further supported by the consistency between the effects of MJ18 treatment and those observed in B7-H3 KO mice and B7-H3 KD cells, including restored TFAM expression, reduced ROS accumulation, and attenuated photodamage. The concordance of these phenotypes provides functional evidence that MJ18 targets the same B7-H3-driven pathway identified throughout this study. Given the well-documented specificity of MJ18 as a validated B7-H3-blocking antibody in the literature, these convergent data from multiple loss-of-function models collectively support the conclusion that the observed protective effects are attributable to B7-H3 blockade rather than off-target antibody effects.

Nevertheless, we acknowledge that direct biochemical evidence for MJ18 binding to B7-H3 is not provided here. The precise molecular mechanism remains to be fully defined, particularly whether MJ18 specifically blocks the B7-H3/ITGA2 interaction identified in this study or acts through other B7-H3-associated proteins such as IL20RA, MVP, or Fn, which have been shown to mediate diverse functions in tumor biology independent of adaptive immunity[51-53]. In addition, the use of a micro-needle roller for local antibody delivery may introduce confounding factors such as needle-induced inflammation or wound healing responses, which could influence the observed therapeutic effects independently of B7-H3 blockade. Although microneedling is a well-established technique in dermatology for enhancing transdermal drug delivery and clinical studies indicate that its primary role is to facilitate drug penetration rather than to confer direct therapeutic benefit, we cannot completely exclude the possibility that the micro-needling procedure itself contributed to the observed effects. Extending beyond its well-characterized role in tumor biology, our findings suggest a role for B7-H3 in non-tumor pathologies, indicating a broader functional paradigm for B7-H3 beyond oncological contexts. Therefore, further

studies with validated target specificity and improved delivery methods are warranted to confirm the therapeutic potential of B7-H3 targeting. Taken together, these data support the feasibility of targeting B7-H3 for photodamage intervention. Our mechanistic exploration using genome-wide B7-H3 KO models has delineated its photoprotective axis, though cell-type-specific functional dissection through conditional knockout (CKO) approaches would add another layer of precision to these findings. ”

We hope this revised version addresses the reviewer's concern.

9.Figure legends

- **Define UV conditions and timepoints in each legend (UVA vs UVB, dose, exposure schedule, harvest time).**

We have revised all figure legends to include detailed irradiation conditions as requested. Each legend now explicitly specifies UVA or UVB, dose per exposure, exposure schedule, and sampling time points for all *in vivo* and *in vitro* experiments.

- **Use consistent terminology (UVR with explicit UVA/UVB specification, and consistent use of "photodamage/photoaging/acute photodamage" with definitions).**

In response to the reviewer's comments, we have carefully revised all figure legends to include detailed irradiation conditions as requested. Each legend now explicitly specifies the UV source (UVA or UVB), dose per exposure, exposure schedule, and sampling time points for all in vivo and in vitro experiments. We have also ensured consistent terminology throughout the figure legends: "UVR" is used as a general term with explicit specification of UVA or UVB where applicable; "acute photodamage" is used consistently for short-term UVR exposure experiments; and "photoaging-associated molecular changes" has been adopted in Figure 3 to accurately reflect that these data represent early markers rather than chronic phenotypes, while "photodamaged skin" is used uniformly for human tissue samples. These revisions have improved the clarity and consistency of the figure legends, and we appreciate the reviewer's guidance on this matter.

- **Provide necessary assay details where needed: ROS probe (DCFH-DA/DHE/MitoSOX), flow cytometry details (basic gating description), and how "photodamage score" and "epidermal thickening score" were calculated (or clearly point to Methods).**

We have revised the Methods section to include detailed descriptions of flow cytometry protocols and scoring systems as requested. For ROS detection, we now specify the probes used (DCFH-DA for intracellular ROS, mitoSOX for mitochondrial ROS) and their corresponding detection channels. Debris and doublets were excluded based on FSC/SSC and FSC-A/FSC-H gating, and unstained samples were used as negative controls to establish background fluorescence thresholds. Quantification was performed using geometric mean fluorescence intensity. For TUNEL apoptosis assays, FITC-labeled apoptotic cells were detected by flow

cytometry with the gate set based on unstained controls. The photodamage scoring system (0-2 scale with descriptive criteria) and epidermal thickness measurement protocol (ImageJ-based quantification with randomization and normalization) are documented in the Animal experimentation subsection. These additions ensure that all necessary experimental details are available for reproducibility.

The relevant portions of the revised Methods section are as follows:

Flow cytometry analysis: For all flow cytometry experiments, cells were acquired on a Beckman Coulter CytoFLEX flow cytometer. Debris and dead cells were excluded based on forward scatter (FSC) and side scatter (SSC) gating. A minimum of 10,000 viable single cells were analyzed per sample. For each experiment, unstained controls and single-stained compensation controls were included to set appropriate gates and correct for spectral overlap. (Supplementary File, Page 5, Line 17).

Cell cycle analysis: Cells were collected, washed with ice-cold PBS, fixed with 70% cold ethanol, and stored at 4 °C for 24 h. After centrifugation, cells were washed twice with cold PBS and stained with PBS containing RNase A and propidium iodide (PI) in the dark for 30 min (Beyotime, Suzhou, China, C1052). PI fluorescence was detected in the FL3 channel, and cell cycle distribution (G1, S, G2 phases) was analyzed using FlowJo software. Debris and doublets were excluded by gating on FSC-A vs. FSC-H. (Supplementary File, Page 5, Line 23).

TUNEL assay: Apoptosis was detected using an Elabscience kit (E-CK-A420) following the manufacturer's instructions; FITC-labeled apoptotic cells were detected by flow cytometry. The FITC-positive gate was set based on unstained control cells, and mean fluorescence intensity (MFI) was quantified for each sample. (Supplementary File, Page 5, Line 30).

Intracellular ROS measurement: ROS levels were measured using the DCFH-DA probe (Beyotime, S0033S). Cells were incubated with 1 μM DCFH-DA at 37 °C for 20 min, washed, and immediately analyzed by flow cytometry. The DCF fluorescence (FITC channel) was measured, and the geometric MFI was calculated. Unstained cells were used to set the background fluorescence threshold. (Supplementary File, Page 6, Line 4).

Mitochondrial ROS measurement: Mitochondrial superoxide was assessed using the mitoSOX probe (Beyotime, S0061S). Cells were incubated with 5 μM mitoSOX at 37 °C for 10 min, washed, and immediately analyzed by flow cytometry. MitoSOX fluorescence was detected in the PE channel, and the geometric mean fluorescence intensity was calculated. Unstained cells served as negative controls to establish baseline fluorescence. (Supplementary File, Page 6, Line 9).

Photodamage scoring: Images of the dorsal skin of the mice were captured using a camera, and skin lesion severity was assessed according to established photodamage scoring criteria, which ranged from 0 to 2. The scoring was performed by two independent investigators, and the average score was calculated for each mouse. 0: flesh-colored, smooth, and plump skin; 0.5: flesh-colored, slightly rough skin; 1: red patches with moderately rough skin and deepened skin lines; 1.5: red patches with noticeably rough skin, minor flaking, and some wrinkles; 2: a purple-red ulcerative condition with significantly rough and thickened skin, extensive flaking, and

increased wrinkles. (Page 19, Line 27).

Epidermal thickness measurement: H&E-stained skin sections were imaged under a light microscope (Olympus, Japan) at 200× magnification. For each mouse, three non-overlapping high-power fields were randomly selected and photographed. Epidermal thickness was measured using ImageJ software. After calibrating the scale bar using the image scale, the full epidermal layer (from the top of the stratum corneum to the bottom of the basal layer) was measured at 5-10 randomly selected points per image, and the average value was calculated for that image. The epidermal thickness for each mouse was calculated as the average of three images. To minimize batch-to-batch staining variability and individual differences, data were normalized to the mean epidermal thickness of the WT control group and presented as relative epidermal thickness. (Page 20, Line 5).

10.Clarity, terminology, and typos

We thank the reviewer for their careful reading and helpful suggestions. We have thoroughly reviewed the entire manuscript to address issues related to clarity, terminology consistency, and typographical errors. Grammatical errors have been corrected throughout. Terminology has been standardized, with consistent use of "UVR" as a general term while specifying UVA or UVB where applicable. We have also ensured proper formatting of mathematical symbols and units. All figure legends have been revised to include detailed irradiation conditions and to ensure consistent terminology throughout. These revisions have improved the overall clarity and professionalism of the manuscript.

- **Replace "malignant endpoints of chronic photodamage" with "UV-associated non-melanoma skin cancers commonly linked to chronic sun exposure."**

We appreciate the reviewer's suggestion for more precise terminology. "Malignant endpoints of chronic photodamage" has been replaced with "UV-associated non-melanoma skin cancers commonly linked to chronic sun exposure" in the revised manuscript. (Page 4, Line 26).

- **Replace "direct B7-H3-interacting partner" with "B7-H3-associated interactor supported by IP/MS and co-IP."**

We thank the reviewer for this important clarification regarding the description of protein interactions. The phrase "direct B7-H3-interacting partner" has been revised to "B7-H3-associated interactor supported by IP/MS and Co-IP" to more accurately reflect our experimental evidence. (Page 10, Line 26).

- **Correct the typo: "B7-H3 deletion promotes ROS accumulation" → "reduces ROS accumulation."**

We are grateful to the reviewer for catching this typo. The error B7-H3 deletion promotes ROS accumulation has been corrected to B7-H3 deletion reduces ROS accumulation throughout the manuscript.

- **Ensure consistent NRF1/NRF2 capitalization throughout.**

We appreciate the reviewer's attention to detail. We have carefully reviewed the entire manuscript and confirmed that NRF1 and NRF2 are consistently capitalized throughout the text, figures, and figure legends. No inconsistencies were found.

Reviewer #3 (Remarks to the Author):

1) In Figure 1B, how did the authors differentiate normal and naturally aged skin? If this was based on subject age, it would be useful to provide information somewhere about the age ranges of the subjects in these two groups.

We thank the reviewer for this suggestion. In Figure 1B, the differentiation between normal and naturally aged skin was based on both donor age and clinical characteristics, as evaluated by two qualified dermatologists. We have now provided detailed age information for each group in the revised Methods section (Page18, Line 2).

Based on clinical diagnosis and histopathological evaluation by two qualified dermatologists, the samples were classified into four groups: normal skin (n = 15, obtained from sun-protected areas such as post-auricular and upper inner arm regions of young to middle-aged donors with no history of significant sun exposure and no clinical signs of photoaging, mean age 32.9 ± 5.6 years); naturally aged skin (n = 8, obtained from sun-protected areas of donors presenting with typical senile skin changes including fine wrinkling and laxity attributable to chronological aging, but lacking the characteristic features of photoaging, mean age 57.3 ± 7.9 years); photodamaged skin (n =12, obtained from sun-exposed areas such as face and forearm of donors who, despite regular sunlight exposure, did not exhibit severe clinical signs of photoaging such as deep wrinkles or leathery appearance, mean age 33.8 ± 6.4 years); and photoaged skin (n = 15, obtained from severely sun-exposed areas such as face of donors presenting with classic clinical features of photoaging, including deep wrinkles, leathery appearance, and pigmentary irregularities, mean age 51.3 ± 10.1 years).

2) The authors mention using a UVB phototherapy device and a solar simulator in the methods section but do not provide any information about the model/manufacturer. This information is important should other scientists wish to replicate or extend the findings.

We appreciate the reviewer's comment regarding the UVR device information. Following this suggestion, we have supplemented the animal irradiation protocols in the revised Methods section and the cell irradiation details in the Supplementary Materials with specific model and manufacturer information. Specifically, for animal experiments and HaCaT cells, we used a UVB phototherapy device (SH2 A, Sigma, Shanghai, China). For HSF cells, we used a UVA light source (SH2-J/SC2-J, Sigma, Shanghai, China). The irradiation doses and exposure conditions for each cell model have been clearly described. The revised content has been incorporated in the Animal experimentation and evaluation of photodamage subsection of Methods and the Cell

culture, UVR exposure section in the Supplementary Materials. (Page 19, Line 11).

"To model acute UVB-induced skin damage, mice were irradiated using a UVB phototherapy device (SH2 A, Sigma, Shanghai, China).

For UVR exposure experiments, cells were seeded in culture dishes and grown to 70-80% confluence prior to exposure. HaCaT cells were exposed to UVB using an SS-04B narrowband UVB phototherapy device (SH2 A, Sigma, Shanghai, China) at indicated doses. HSF cells were exposed to UVA using a UVA light source (SH2-J/SC2-J, Sigma, Shanghai, China). The culture medium was replaced with phosphate-buffered saline (PBS) during irradiation to avoid the generation of toxic photoproducts. After irradiation, PBS was removed and fresh culture medium was added, and cells were incubated for indicated time points before further analysis. Control cells were subjected to the same procedure without UVR exposure.

We believe these additions will facilitate other researchers in replicating our experimental conditions.

3) In various places in the text (such as lines 132 and 138), it is not particularly clear from how the text is written which group/treatment is more affected by the treatment. For example, on lines 131-132, the authors state "Consistent with this, both in vivo and in vitro skin photodamage models, elevated ROS levels were observed" but do not state which group (WT or KO) has higher ROS levels. Obviously the reader can look at the figure to determine this, but the text would read better if the results were more clearly stated.

We thank the reviewer for pointing out this lack of clarity in our wording. This was indeed an oversight in our original description. We have revised the text to explicitly state which groups exhibit elevated ROS levels. As shown in Figure 2D-F, while UVR exposure increased ROS levels in all groups, B7-H3 KO mice (or B7-H3 KD cells) exhibited significantly lower ROS accumulation compared to WT mice (or control cells) following UVR exposure. This indicates that B7-H3 deletion attenuates UVR-induced oxidative stress (Page 5, Line 24).

"Consistent with this, UVR significantly increased ROS levels in both *in vivo* and *in vitro* photodamage models compared to non-irradiated controls. Importantly, this UVR-induced ROS accumulation was markedly attenuated in B7-H3 KO mice, as well as in B7-H3 KD HaCaT and HSF cells, when compared to their respective WT or control groups (Fig. 2D-F). Consistent with the elevated ROS levels, UVR induced significant alterations in antioxidant enzyme activities and oxidative damage markers. *In vivo*, UVB-exposed WT mouse skin exhibited a compensatory increase in superoxide dismutase (SOD) activity, alongside a significant reduction in glutathione peroxidase (GSH-Px) activity and elevated malondialdehyde (MDA) levels, indicating an imbalance in antioxidant defense and subsequent lipid peroxidation. Compared to UVB-exposed WT mice, B7-H3 KO mice displayed further enhanced SOD activity, restored GSH-Px activity, and markedly reduced MDA accumulation (Fig. 2G). In UVB-irradiated HaCaT and UVA-irradiated HSF cells, knockdown of B7-H3 significantly attenuated the suppression of SOD, catalase (CAT), and GSH-Px activities and reduced MDA accumulation compared to control cells (Fig. 2H, I). "

We believe this revision clearly communicates the comparative findings and appreciate the reviewer's help in improving the clarity of our manuscript.

Thank you again for your time and consideration. We look forward to your positive response.

Lei Cao
caolei123@suda.edu.cn
Jiangsu Institute of Clinical Immunology
The First Affiliated Hospital of Soochow University
178 Ganjiang East Road, Suzhou, Jiangsu Province, China
