# Supplementary material for: B7-H3 promotes skin photodamage through ITGA2-NRF2-TFAM-mediated mitochondrial oxidative stress
Source: Commun Biol. 2026 May 28;9:1134. doi: 10.1038/s42003-026-10387-6 (PMC13500712; doi:10.1038/s42003-026-10387-6)
Supplement: Supplementary file 4 [file 42003_2026_10387_MOESM2_ESM.docx]

**Description of Additional Supplementary Files**

**File name:** Supplementary Data 1

**Description:** Nucleic acid reagent sequences, including plasmid reference sequence (gbk format) and siRNA sequences.

**File name:** Supplementary Data 2

**Description:** Numerical source data (Excel file) for the graphs presented in the figures.
